# Bioinspired Self-Assembly-Reinforced Ion Transport and Interface Regulation Enables Sustainable Metal-Ion Batteries for Wearable Electronics

**DOI:** 10.1007/s40820-026-02071-5

**Published:** 2026-01-26

**Authors:** Kang Ma, Ran Zeng, Shuang Chen, Yu Zhang, Jiqian Wang, Xuzhi Hu, Yinzhu Jiang, Hai Xu, Hongge Pan, Deqing Mei, Ehud Gazit, Kai Tao

**Affiliations:** 1 State Key Laboratory of Fluid Power and Mechatronic Systems, Zhejiang Key Laboratory of Advanced Equipment Manufacturing and Measurement Technology, School of Mechanical Engineering, Zhejiang University, Hangzhou, 310058 People’s Republic of China; 2 Zhejiang-Israel Joint Laboratory of Self-Assembling Functional Materials, ZJU-Hangzhou Global Scientific and Technological Innovation Center, Hangzhou, 311215 People’s Republic of China; 3 Zhejiang-Ireland Joint Laboratory of Bio-Organic Dielectrics & Devices, Zhejiang University, Hangzhou, 310058 People’s Republic of China; 4 State Key Laboratory of Silicon and Advanced Semiconductor Materials, School of Materials Science and Engineering, Zhejiang University, Hangzhou, 310058 People’s Republic of China; 5 Department of Biological and Energy Chemical Engineering, China University of Petroleum (East China), 66 Changjiang West Road, Qingdao, 266580 People’s Republic of China; 6 State Key Laboratory of Solid Lubrication, Lanzhou Institute of Chemical Physics, Chinese Academy of Sciences, No.18, Tianshui Middle Road, Lanzhou, 730000 People’s Republic of China; 7 Institute of Science and Technology for New Energy, Xi’an Technological University, Xi’an, 710021 People’s Republic of China; 8 The Shmunis School of Biomedicine and Cancer Research, George S. Wise Faculty of Life Sciences, Tel Aviv University, 6997801 Tel Aviv, Israel; 9 Department of Materials Science and Engineering, Faculty of Engineering, The Iby and Aladar Fleischman, Tel Aviv University, 6997801 Tel Aviv, Israel

**Keywords:** Self-assembly, Synergistic ion transport and interphase regulation, Metal-ion batteries, Bionic structures, Wearable electronics

## Abstract

The rapid growth of wearable electronics demands power sources that are not only flexible and durable but also inherently safe. Conventional lithium-ion batteries pose safety risks due to toxic and flammable electrolytes. Aqueous metal-ion batteries offer a promising alternative, yet their application remains limited by poor mechanical compliance, leading to interfacial instability and electrolyte leakage. Here, we report a bionic self-assembly strategy for aqueous zinc-ion batteries using a lipopeptide electrolyte additive named C_16_K, enabling bulk self-assembly into supramolecular nanohelices to accelerate ion transport and interfacial organization into a dynamic bilayer for interphase regulation. This dual-function synergistically suppresses the formation of Zn dendrites or side reactions, enabling stable Zn plating/stripping. This achieves an ultralong cycling stability and ultrahigh cumulative plating capacity along with a high coulombic efficiency. Therefore, the synergistic reinforcement endows the pouch cell to deliver a high initial capacity, allowing to power electronics in a safe manner. In a following manner, a scorpion tail-inspired bionic flexible battery structure is designed to deliver sustainable energy outputs across various mechanical states using the reinforced systems, effectively powering the wearable multimodal sensors. Our results present a self-assembly strategy using a lipopeptide additive to synergistically reinforce the ions transport and interfacial stability, coordination with a bionic structural design, potentially offering a bioinspired routine for high-performance flexible batteries for wearable electronics.

**Supplementary Information:**

The online version contains supplementary material available at 10.1007/s40820-026-02071-5.

## Highlights

- Bionic self-assembly strategy forms bulk supramolecular nanohelices and interfacial dynamic bilayers to enhance ion transport and interfacial stability.
- Reinforced asymmetric cells exhibit a high coulombic efficiency of 99.66% over 2400 cycles, while the corresponding full cells retain 86% of their initial capacity after 1000 cycles, demonstrating outstanding electrochemical stability and durability.
- Scorpion tail-inspired flexible battery design provides stable energy output under various mechanical states and powers wearable sensors.

**Supplementary Information:**

The online version contains supplementary material available at 10.1007/s40820-026-02071-5.

## Graphical abstract

**Figure:**
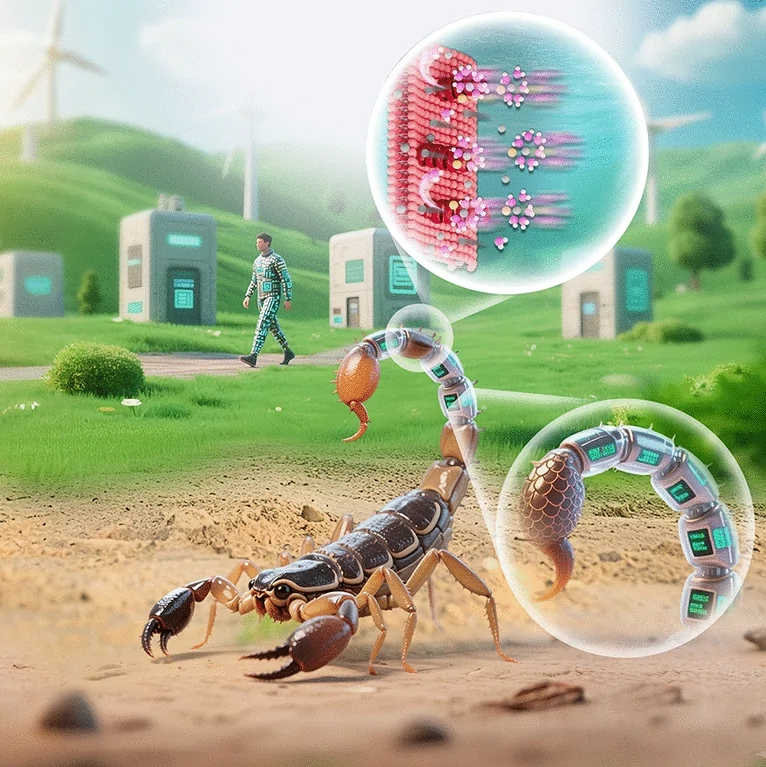

## Introduction

The integration of multimodal sensing systems for bioelectronics and bio-machine interface has gained increasing attention in wearable electronics [1] and has been showing promising potentials for applications in monitoring physiological signals such as movement status, body temperatures, sweat metabolites, and heart rates [2–5]. Given that the functionality of the sensors and the transmission of data within these systems are dependent on a constant and stable power supply, the seamless integration of power sources with multimodal sensing systems is inevitable for developing advanced wearable technologies [6]. In this regard, traditionally rigid batteries are no longer appropriate for bendable, stretchable, and fordable applications [7]. Although supercapacitors offer high power densities and rapid charging capabilities, their reliance on non-Faradaic energy storage modes leads to severe self-discharge [8]. On the other hand, state-of-the-art lithium-ion batteries (LIBs) have been extensively studied to be employed as flexible power sources due to the high energy densities (250–400 Wh kg^−1^); their reliance on flammable and toxic organic electrolytes presents tough challenges to the sustainability and safety for stable and long-term power needs. Additionally, the limited availability of lithium resources, coupled with the harsh manufacturing conditions, results in high costs and hinders the widespread adoption of LIBs [9].

Therefore, the development of aqueous flexible batteries that provide stable power output, extended operational lifetimes, and adaptability to complex geometries has become a key issue for the advancement of wearable electronics. Regarding this, aqueous zinc-ion batteries (ZIBs) show great potential for applications in wearable electronics as flexible power systems, owing to their low cost, high safety, and durable sustainability [10]. Metallic Zn provides a high volumetric capacity (5855 mAh cm^−3^, approximately three times that of LIBs with 2060 mAh cm^−3^) and possesses a favorable electrode redox potential (-0.762 V *vs.* standard hydrogen electrode, SHE) [11]. However, the ZIBs suffer from irreversible issues including the formation of uncontrolled Zn dendrites during cycling and severe side reactions such as self-corrosion and hydrogen evolution reaction (HER) at the anode/electrolyte interface, along with the asynergy between ions transport in the bulk solution and ions flux at the interface, which lead to short circuits and ultimately an attenuated calendar life of the batteries [12]. Many efforts have been put forth to address these challenges, with representative electrolyte additives offering an effective way to modulate electrolyte properties by optimizing metal-ion solvation structures and generating protective layers [13, 14]. Nevertheless, in most cases the amphiphilic nature urges the additives to self-assemble into supramolecular architectures in the electrolytes, which usually impede the metal ions transport due to capturing and encapsulation. This can aggravate the asynergy between ions transport and ions flux [15]. In addition, the limited deformation capability of zinc foil and cathode current collector (*e.g.,* Ti foil or carbon cloth) may lead to the potential leakage of electrolytes and separator–electrode separation under deformation [16, 17]. Collectively, these issues may severely compromise the performances of aqueous wearable metal-ion batteries [17].

After thousands of years evolution, nature represents the pinnacle of engineering, where bionic structure–function coupling offers innovative design paradigms for highly efficient systems [7, 18–21]. Specifically, the helical ion channels are evolved to accelerate the transport of entities through the cells [22, 23]. While the ultrathin, self-adaptive, and dynamic bilayers (such as phospholipid membranes) can high-efficiently and selectively capture and release targets [21, 24]. Inspired from these principles, the synergistic improvement of bulk ion transport and interfacial stability is simultaneously achieved through the dual self-assembly into helical structures and self-adaptive bilayers of a minimalistic lipopeptide composed of an aliphatic chain and an amino acid (palmitoyl-lysine, C_16_K) [25, 26]. Specifically, the C_16_K molecules self-assemble into supramolecular nanohelices in the solutions, mimicking the biological ion channels to accelerate the metal-ion transport. While the simplistic lipopeptide dynamically forms ultrathin bilayers at the interface, akin to cellular membranes, thus homogenizing the ion flux and electric field distributions. In contrast to the commonly accepted concept of asynergy, this synergistic effect enables uniform metal-ion plating/stripping and high efficiency to address the formation of dendrites or side reactions, thus notably reinforcing the CE of the aqueous metal-ion batteries. This allows the pouch cell to deliver a high initial capacity to power electronic devices in a safe and eco-friendly manner. Further drawing inspiration from the rigid-flexible coupling of a scorpion’s tail [27], the wearable metal-ion batteries of bionic conformations are engineered using the reinforced metal-ion batteries, thereby providing exceptional mechanical adaptability once encountering diverse deformations for integrated multimodal sensing applications. This represents a substantial step toward high-performance and sustainable secondary batteries for wearable electronics.

## Experimental Section

### Materials

The palmitic acid with purity of ≥ 95% was obtained from Alfa Aesar (China) Chemical Co., Ltd. Fmoc-Lys(Boc)-OH, 1-hydroxybenzotriazole anhydrous (HOBt), O-(1H-benzotriazol-1-yl)-N,N,N’,N’-tetramethyluronium hexafluorophosphate (HBTU), N,N-diisopropylethylamine (DIEA), and Rink amide MBHA resin were purchased from GL Biochem (Shanghai) Ltd. Triisopropylsilane (TIS), hexafluoroisopropanol (HFIP), dichloromethane (DCM), trifluoroacetic acid (TFA), and N,N-dimethylformamide (DMF), all of peptide synthesis grade, were supplied from BMJ Technology (Beijing) Co. Ltd. Chloroform, methanol, petroleum ether, acetonitrile, and N-methyl-2-pyrrolidone (NMP) were purchased from Sigma-Aldrich (Shanghai) Trading Co., Ltd and used without further purification. Zinc foils with thickness of 100 μm or 20 μm were purchased from Tianjin EVS Chemical Technology Co., Ltd. Specifically, zinc foils with thickness of 17 μm were prepared via repeating rolling of commercial Zn foils. Glass fiber separators (GF/D) were purchased from Cytiva Bio-technology (Hangzhou) Co., Ltd. Zinc sulfate heptahydrate (ZnSO_4_·7H_2_O, AR) and sodium sulfate (Na_2_SO_4_, AR) were purchased from Sinopharm Chemical Reagent Co., Ltd. Copper, titanium foils, coin-type cells (CR2025), and Super P were purchased from Guangdong Canrd New Energy Technology Co., Ltd. Ultrapure water (18.2 MΩ cm) was processed using a Milli-Q purification system.

### Lipopeptide Synthesis and Analysis

Lipopeptide synthesis was performed on a CEM Liberty Lite microwave peptide synthesizer, which facilitated deprotection, coupling, and cleavage reactions via microwave-assisted Fmoc solid-phase peptide synthesis [28]. The synthesis was initiated on Rink amide MBHA resin to ensure C-terminal amidation. Resin deprotection was carried out with 20% piperidine and 0.1 M HOBt in DMF, followed by coupling of Fmoc-Lys(Boc)-OH. Palmitic acid was subsequently conjugated to the peptide chain using HBTU/HOBt/DIEA for carboxyl activation and amide bond formation. Upon completion of synthesis, the lipopeptide was cleaved from the resin and the Boc-protecting group on lysine was removed using a TFA/TIS/H_2_O (a ratio of 95:2.5:2.5) cleavage mixture. The cleavage solution and DCM washings were collected, filtered into a round-bottom flask, and purged with nitrogen to remove residual volatile components. The treated raw product was subsequently dissolved into chloroform and extracted by mixing with water using a separation funnel. After collection of the product from evaporation of chloroform, it was further dissolved in methanol and then extracted by adding petroleum ether (boiling point around 60 °C). After being extracted by petroleum ether a few times, the peptide was collected by evaporating the methanol phase. The final collected solid product was lyophilized for 2 days and then subjected to reversed-phase HPLC (RP-HPLC) purification and mass spectrometry (MS) analysis to verify the purity [28, 29]. The lyophilized lipopeptide powders were then dissolved in 2.0 M ZnSO_4_ aqueous solution to prepare the electrolytes with different concentrations.

The RP-HPLC analysis was conducted using a Waters 2695 Alliance system at 25 ± 2 °C. Prior to analysis, the lipopeptide aqueous solution was filtered through a 0.4-μm filter and subsequently injected into a Fortis Xi C18 reversed-phase column (250 × 4.6 mm^2^, 5 um particle size). Chromatographic separation was achieved using gradient elution with two mobile phases: solvent A (0.1% TFA in water) and solvent B (0.1% TFA in acetonitrile). The elution gradient was programmed as follows: 0–0.1 min, 36% A; 0.1–25.0 min, 36% → 11% A; 25.0–25.1 min, 11% → 0% A [28]. The flow rate was set to 1.0 mL min^−1^, and UV detection was performed at 220 nm. For MS analysis, a Bruker Biflex III matrix-assisted laser desorption/ionization time-of-flight (MALDI-TOF) mass spectrometer equipped with a 337-nm nitrogen laser was employed. α-Cyano-4-hydroxycinnamic acid was used as the matrix. The lipopeptide was dissolved in a 1:1 (*v/v*) acetonitrile/water mixture containing 1% TFA. A 0.8-μL aliquot of the sample–matrix mixture was spotted onto a metal sample plate and left to air-dry at ambient temperature. Mass spectra were acquired in the positive linear mode under an acceleration voltage of 20 kV, with external calibration performed using a standard material. To ensure reliable data acquisition, 120 laser pulses were accumulated and averaged, with laser power adjusted to remain just above the ionization threshold.

### Preparation of Cathode Materials and Electrode Fabrications

MnO_2_ was synthesized via a hydrothermal method. 0.474 g of KMnO_4_ and 2.718 g of MnSO_4_ were each dissolved in 30 mL of deionized water. The solutions were rapidly mixed and stirred for 30 min, resulting in a purple-brown color. The mixed solution was then transferred to a Teflon-lined autoclave and heated at 140 °C for 12 h. After cooling to room temperature, the obtained precipitate was washed for several times with deionized water, followed by drying at 80 °C for 12 h. The obtained MnO_2_ was ground into a fine powder using an agate mortar. To prepare (NH_4_)_2_V_4_O_9_, 1.0 g of NH_4_VO_3_ was dissolved in 50 mL of deionized water and stirred for 10 min to obtain a light-yellow solution. Then, 0.45 g of C_2_H_2_O_4_·2H_2_O was added to the solution and stirred for an additional 20 min to yield a reddish-orange mixture. The mixture was then transferred to a PTFE-lined reaction vessel (100 mL) and subjected to hydrothermal treatment at 200 °C for 20 h. The product was collected by centrifugation, washed for several times with deionized water and anhydrous ethanol, and then dried in a vacuum oven at 60 °C for 24 h.

The surface of Zn foils was polished to remove native oxide layers. The polished Zn foils were then punched into disks or cut into rectangles for use as Zn electrodes. Unless otherwise specified, the thickness of the Zn foil was 100 μm. A commercial Cu foil with a thickness of 80 µm was used as the current collector and was subjected to the same treatment as the zinc electrode described above. The MnO_2_/(NH_4_)_2_V_4_O_9_ cathode used in the coin cells was prepared by mixing MnO_2_/(NH_4_)_2_V_4_O_9_, super P, and polyvinylidene fluoride (PVDF) with a mass ratio of 7:2:1 in NMP and casted onto the Ti foils (10 μm). The electrodes were dried at 60 °C for 12 h in vacuum. The mass loading of the active material was approximately 1.8 mg cm^−2^. To achieve higher mass loading while keeping environmental sustainability, MnO_2_ cathode for pouch cells were fabricated by dispersing MnO_2_, Super P, and a water-based composite binder (carboxymethylcellulose and styrene-butadiene rubber at a ratio of 2: 3) in a mass ratio of 80: 15: 5 in water. The mixture was homogenized using an ultrasonic mixer to form a stable dispersion and then was coated onto a graphite paper (100 μm). The electrodes were vacuum-dried at 60 °C for 12 h, achieving the active material loading of around 10.0 mg cm^−2^.

### Critical Micelle Concentration Determinations

Pyrene Fluorescence Probe Method: Due to the extremely low aqueous solubility of pyrene, a stock solution was first prepared in methanol at a concentration of 0.01 mM. A 100-µL aliquot of stock solution was added to the test tube. After complete evaporation of methanol, 10 mL of the lipopeptide solution with 2.0 M ZnSO_4_ was introduced. To achieve uniform dispersion of pyrene within the lipopeptide solution, the mixture was subjected to ultrasonication for 30 min. The resulting solution contained pyrene at a final concentration of 1.0 × 10^–4^ mM. The prepared solution was then transferred to a quartz cuvette and analyzed using a Hitachi F-4500 fluorescence spectrophotometer (Japan) with an excitation wavelength of 334 nm. Emission spectra were recorded over the range of 350–450 nm. The CMC of the lipopeptide was determined by plotting the fluorescence intensity ratio (*I*_*1*_/*I*_*3*_) versus concentrations, where *I*_*1*_ and *I*_*3*_ represent the intensities at 373 and 384 nm, respectively. The CMC was identified as the inflection point of the* I*_*1*_/*I*_*3*_ versus concentration curve. Surface Tension Method: The surface tension measurements were taken using a Krüss K11 tensiometer based on the du Noüy ring method [28]. A platinum-iridium alloy ring was immersed in the test lipopeptide solution with 2.0 M ZnSO_4_ and gradually withdrawn, during which the force exerted on the ring arose from both gravitational and surface tension forces. The tensiometer automatically corrected the surface tension values using the Harkins and Jordan equation to ensure accuracy. As the lipopeptide concentration increased, the surface tension of the solution progressively decreased until the CMC was reached. Further increases in concentration led to negligible changes in surface tension, thus enabling the determination of the CMC from the inflection point of the surface tension versus concentration curve. Conductivity Method: The C_16_K solutions with 2.0 M ZnSO_4_ in a range of concentrations were prepared, and a 1.0-M KCl solution was used as a standard reference. The impedance of both the lipopeptide solutions and the reference solution was measured using an Ivium electrochemical workstation. Given that conductivity is inversely related to resistivity, the conductivity of each lipopeptide solution was calculated by comparing its impedance to that of the standard, whose conductivity was obtained from established literature values. To determine the CMC, the conductivity was plotted as a function of lipopeptide concentrations. Two distinct linear regions were observed, and the intersection point of the two fitted lines was taken as the CMC.

### Spectroscopic Ellipsometry and Neutron Reflection

The SE measurements were taken using a Jobin–Yvon UVISEL spectroscopic ellipsometer over a typical wavelength range of 300–600 nm. A liquid cell specially constructed for facilitating the SE measurement at the solid–liquid interface with an incident light at 70° with respect to the sample surface was used. The experimental data were analyzed to give the thickness (τ) and the optical constant (the refractive index, n) of the layer involved, using the software called DeltaPsi2 developed by Jobin–Yvon. The amount of the lipopeptide adsorbed is calculated using Eq. (1) proposed by De Feitjer et al*.* [28].

1 $$\Gamma =\frac{\tau (n-{n}_{b})}{a}$$

where n is the refractive index of the layer with thickness τ, ${n}_{b}$ is the refractive index of the aqueous surfactant solution, and $a=\frac{d{n}_{b}}{dc}$, indicating the change in the refractive index of the C_16_K solution with increasing concentration. Its value is close to 0.18 cm^3^ g^−1^ for a variety of surfactants [29].

The NR measurements were taken at the SURF reflectometer, Rutherford Appleton Laboratory (Oxford, UK,), utilizing neutron beams with wavelengths ranging from 0.5 to 6.5 Å. Samples were prepared by clamping a stainless steel trough against the polished face of a silicon block (< 111 > orientation; 6 cm × 5 cm × 1.2 cm). The sample cell was mounted on a computer-controlled goniometer stage and filled with approximately 2 mL of the test solution, including ordinary lipopeptide in H_2_O with 2.0 M ZnSO_4_, deuterated lipopeptide in D_2_O with 2.0 M ZnSO_4_, and deuterated lipopeptide in H_2_O with 2.0 M ZnSO_4_. The neutron beam entered through the narrow edge of the silicon block, reflected from the solid–liquid interface, and exited from the opposite side. Horizontal and vertical slits were used to define the beam profile, resulting in an illuminated area of about 4 cm × 3 cm on the interface. Reflectivity measurements were taken at three incidence angles (0.35°, 0.8°, and 1.8°), covering a wave vector (κ) range from 0.012 to 0.5 Å^−1^. Below the critical angle, reflectivity was expected to be unity and used for normalization. A constant background signal, typically around 2.0 × 10^–6^ in both D_2_O and H_2_O (with 2.0 M ZnSO_4_), was subtracted based on the average reflectivity between 0.3 and 0.5 Å^−1^. The obtained reflectivity profiles were analyzed using a model-fitting approach based on the optical matrix formula. The fitting procedure was initiated with an assumed interfacial structural model, followed by the calculation of the theoretical reflectivity, which was then compared with the experimentally measured data. The procedure was iterated until a good fit was obtained.

### Microscopy and Spectroscopy Characterizations

For AFM measurements, 10 µL of a 2.0-M ZnSO_4_ solution containing the lipopeptide was dispensed onto the freshly cleaved mica surface and incubated for 3 min to enable the adsorption of C_16_K molecules into a stable structure at the mica interface. Excess solution was subsequently removed by gently tilting the mica and applying a nitrogen gas stream to avoid disruption of the assembled structures. AFM images were obtained using a Bruker Dimension Icon system. For TEM measurements, the lipopeptide solutions with 2.0 M ZnSO_4_ of various concentrations were prepared and allowed to self-assemble for 24 h. Ten microliters of each sample was applied onto carbon-coated copper grids and left for 2 min to promote adhesion of the self-assembled structures. Excess solution was removed with filter paper. Subsequently, 10 µL of the uranyl acetate solution at 2.0 wt% was added to the grid to enhance image contrast. After adhesion for another 2 min and drying with filter paper, the grids were observed using a JEM-2100F transmission electron microscope operated at an accelerating voltage of 120 kV to examine the self-assembly at various concentrations. Surface morphologies were further characterized by SEM equipped with EDS (Hitachi SU8600, Japan). Additional morphological and topographic analyses were conducted using CLSM (Olympus LEXT OLS4100, Japan) and optical microscopy (YUESCOPE YM10R, China). Surface roughness measurements were obtained by white-light interferometry (Zygo NewView 8200, USA).

The XRD patterns were recorded using a Shimadzu XRD-6000 diffractometer equipped with a CuK_α_ radiation source (λ = 1.5416 Å). FTIR measurements were taken in attenuated total reflectance (ATR) mode using a Thermo Scientific Nicolet iS50 spectrometer, with a scan range of 4000 to 400 cm^−1^. XPS analysis was conducted using a Thermo Scientific ESCALAB 250Xi instrument with an Al K_α_ X-ray source (1486.6 eV). Contact angle measurements of the electrodes were taken using a video-based contact angle goniometer (OCA 20, Dataphysics, GER). ^1^H-NMR spectra were obtained on a Bruker 600 MHz spectrometer using deuterated water (D_2_O) for field frequency locking. Raman spectra were acquired using an XploRA Plus confocal Raman microscope (Horiba, Japan) with a range of 4000–50 cm^−1^. For zeta potential measurements, the Zn powder (1 mg mL^−1^) was dispersed in various electrolyte solutions (2.0 M ZnSO_4_ aqueous solutions) and allowed to equilibrate for 2 h at room temperature. The zeta potentials of the resulting dispersions were determined using a Zetasizer Nano-ZS instrument (Malvern, UK).

### Scorpion Tail-mimic Flexible Battery Fabrications

A multilayered stack composed of Zn anode, separator, cathode, and insulating layer was processed into a long strip with multiple perpendicular branches. Each branch was subsequently wrapped around the central trunk, forming densely-stacked, metamere-like energy storage units. The unwrapped flexible sections interconnected the metamere-like stacks, ensuring structural integrity while imparting remarkable flexibility to the overall device (Fig. S1a). Finally, the system was encapsulated with polydimethylsiloxane (PDMS) via thermal curing to prevent electrolyte leakage and ensure robust electrode–separator contact under various deformation conditions, allowing the flexible battery to be completed (Fig. S1b). All wearable device experiments were strictly conducted in accordance with the ethical requirements approved by the Ethics Committees of Zhejiang University and Westlake University (AP#22-038-3-GCC-3, approval date: 01 December 2022, expiration date: 30 November 2025).

### Electrochemical Measurements

The electrochemical performance of the Zn anodes was evaluated using 2025-type coin cells where Whatman GF/D glass fiber film functioned as the separator. For pouch cells, cathodes (7.9 cm × 5.5 cm), Zn anodes (8.2 cm × 5.7 cm, thickness: 80 μm), and separators (8.5 cm × 6.0 cm) were assembled. The electrolyte consisted of either 2.0 M ZnSO_4_ or 2.0 M ZnSO_4_ containing C_16_K was used in Zn||Zn, Zn||Cu, Zn||MnO_2_, and Zn||(NH_4_)_2_V_4_O_9_ cells. Electrochemical measurements, including cyclic voltammetry (CV), linear sweep voltammetry (LSV), potentiodynamic scanning (Tafel), chronoamperometry (CA), and electrochemical impedance spectroscopy (EIS) measurements, were taken on the CHI660E electrochemical workstation. For Zn||Cu cells, CV curves were recorded at a scan rate of 2 mV s^–1^ within a potential range of -0.3 to 0.6 V. Zn||MnO_2_ full cells were tested at 0.5 mV s^−1^ between 0.8 and 1.8 V, while Zn||(NH_4_)_2_V_4_O_9_ full cells were scanned at 0.5 mV s^−1^ within 0.2–1.6 V. Hydrogen evolution behavior was evaluated using LSV at 5 mV s^−1^ with Ti foil as the working electrode, Zn foil as the counter electrode, and Ag/AgCl as the reference electrode. Tafel plots were recorded at a scan rate of 0.01 V s^−1^ with Zn foil as the working electrode, Ti foil as the counter electrode, and Ag/AgCl as the reference electrode. CA measurements were taken at a fixed overpotential of -200 mV. The EIS measurements were taken over a frequency range of 0.1–10^6^ Hz. Unless otherwise stated, all electrochemical experiments were conducted at 25 °C.

Measurements of Zn^2+^ transfer number. The Zn^2+^ transfer number was determined using the potentiostatic polarization method, as described by Eq. (2):

2 $${t}_{{\mathrm{Zn}}^{2+}}=\frac{{I}_{\mathrm{SS}}(\Delta V-{I}_{0}{R}_{0})}{{I}_{0}(\Delta V-{I}_{\mathrm{SS}}{R}_{\mathrm{SS}})}$$

where ${I}_{\mathrm{SS}}$ and ${I}_{0}$ represent the steady-state and initial currents, respectively, and ${R}_{\mathrm{SS}}$ and ${R}_{0}$ denote the charge-transfer resistances at steady-state and initial conditions. The applied overpotential (ΔV) was fixed at 20 mV.

Measurements of Zn^2+^ desolvation and transport behavior. The Zn deposition process is governed by the efficient desolvation of Zn^2+^. The activation energy (*E*_*a*_) was calculated using the Arrhenius equation by analyzing the EIS data of the symmetrical cells at different temperatures. The relationship is expressed by Eq. (3):

3 $$\frac{1}{{R}_{ct}}=Aexp \left(\frac{-{E}_{a}}{RT}\right)$$

where ${R}_{ct}$ is the charge-transfer resistance, A is the frequency factor, R is the gas constant, and T is the absolute temperature.

Measurements of exchange current density. The Zn plating/stripping kinetics is governed by the exchange current density (${i}_{0}$), which is determined using Eq. (4):

4 $$i={i}_{0}\frac{2F}{RT}\eta$$

where i represents the (dis)charge current density, F is Faraday constant, R is the gas constant, T is the absolute temperature, and η denotes the total overpotential.

Measurements of Zn^2+^ conductivity. The ionic conductivity (σ) of Zn^2+^ in different electrolytes was measured using Cu electrodes and is calculated according to Eq. (5):

5 $$\sigma =\frac{L}{RA}$$

where σ represents the ionic conductivity, L is the distance between two electrodes, R is the bulk resistance, and A is the electrode area.

Measurements of EDL capacitance. The electric double-layer capacitance was calculated from CV tests using Eq. (6):

6 $$C=\frac{{i}_{c}}{v}$$

where C is the capacitance, ${i}_{c}$ is the double-layer current, and v is the scan rate. The linear fit of ${i}_{c}$ versus v was used to extract the capacitance from the slope.

The double-layer current ${i}_{c}$ was defined as half of the current difference between the forward and reverse scans at 0 V, which is calculated using Eq. (7):

7 $${i}_{c}=\frac{{i}_{0}^{v+}-{i}_{0}^{v-}}{2}$$

where ${i}_{0}^{v+}$ and ${i}_{0}^{v-}$ represent the forward and reverse scan currents at 0 V, respectively.

Measurements of the Zn depth of discharge (DoD_Zn_). The DOD_Zn_ for a Zn metal anode using Zn foil is calculated using Eq. (8):

8 $${DoD}_{Zn}=\frac{y}{{C}_{Zn, volume}\cdot x \times {10}^{-4}}\times 100\%$$

where x (μm) is the thickness of the Zn foil, y (mAh cm^−2^) represents the Zn areal capacity applied in electrochemical testing, and ${C}_{Zn, volume}$ is the theoretical volumetric capacity of Zn (mAh cm^−3^).

Measurements of the galvanostatic intermittent titration technique (GITT). The cell was discharged/charged for 10 min at 0.1 A g^−1^ and then relaxed for 40 min to reach the voltage equilibrium. The ion diffusion coefficient is calculated by Eq. (9):

9 $${D}_{\mathrm{GITT}}=\frac{4}{\pi \tau }{\left(\frac{{n}_{\mathrm{m}}{V}_{\mathrm{m}}}{S}\right)}^{2}{\left(\frac{\Delta {E}_{s}}{\Delta {E}_{\tau }}\right)}^{2}$$

where τ, ${n}_{\mathrm{m}}$, ${V}_{\mathrm{m}}$, and S present the constant current pulse duration, the amount of active material, molar volume, and the electrode–electrolyte interface, respectively. $\Delta {E}_{s}$ is the steady-state voltage change under the current pulse. $\Delta {E}_{\tau }$ is voltage change under the constant current pulse after the eliminating of *i*R drop.

### Computational Calculations and Simulations

Ab initio calculations. The first-principles calculations based on density functional theory (DFT) were conducted using the Vienna Ab initio Simulation Package (VASP) [30]. The exchange–correlation energy was described using the Perdew–Burke–Ernzerhof (PBE) functional within the generalized gradient approximation (GGA) [31]. The electronic structure was described using projected augmented wave (PAW) potentials [31], with valence electrons treated within a plane-wave basis set at a cutoff energy of 450 eV. Partial occupancies of the Kohn–Sham orbitals were accounted for using Gaussian smearing with a width of 0.05 eV. The electronic self-consistency criterion was set to an energy convergence threshold of 10^–5^ eV, while geometric optimization was considered converged when the force on each atom was below 0.02 eV Å^−1^. A vacuum layer of 18 Å was introduced perpendicular to the surface to eliminate periodic interactions. Van der Waals interactions were incorporated using the DFT + D3 method with Grimme’s empirical correction [32]. The adsorption energy (*E*_ads_) is determined according to Eq. (10):

10 $$E_{{{\mathrm{ads}}}} = E_{{{\mathrm{total}}}} - E_{{{\mathrm{substrate}}}} - E_{{{\mathrm{adsorbate}}}}$$

where *E*_total_, *E*_substrate_, and *E*_adsorbate_ represent the energy of the entire system after adsorption, the energy of surface model before adsorption, and the energy of adsorbed molecules, respectively.

Quantum chemistry (QC) calculations. All calculations were conducted using Gaussian 16 (Revision C.01). The hybrid PBE0 functional combined with the D3 version of Grimme’s dispersion correction with Becke–Johnson damping (DFT-D3BJ) was employed [32]. Geometry optimizations and frequency calculations were performed using the 6–311 + G(d,p) basis set. To account for solvation effects, the Solvation Model Density (SMD) implicit solvation model [33] was applied throughout. The interaction energy between A and B is computed as Eq. (11):

11 $$E_{{{\mathrm{bind}}}} = E_{{{\mathrm{complex}}}} - \, \left( {E_{{{\mathrm{partA}}}} + E_{{{\mathrm{partB}}}} } \right)$$

where *E*_complex_ denotes the total energy of the complex, while *E*_partA_ and *E*_partB_ correspond to the energies of the isolated species A and B, respectively.

CGMD simulations. All CGMD simulations were conducted using the GROMACS 4.6.7 package with the GROMOS 96 force field [34]. Atomic parameters, such as bond lengths, angles, and dihedral angles, were derived using the Automated Topology Builder (ATB) Version 3.0. Partial charges on the atoms were calculated with DFT, utilizing the B3LYP functional in Gaussian 16 and the 6–31 + G(d,p) basis set. Two distinct simulation models were constructed to investigate the migration behavior of Zn^2+^ in C_16_K self-assembled bilayer and the self-assembly of C_16_K into nanohelices in the electrolyte. The first model involved the placement of two test molecules symmetrically within a 5 nm × 5 nm × 10 nm simulation box, followed by the addition of ZnSO_4_ molecules (8 Zn^2+^ and 8 SO_4_^2−^) and 2811 water molecules. The second model, which represented a larger system, incorporated 200 C_16_K molecules, 50 Zn^2+^ and 50 SO_4_^2−^ ions, and 28,393 water molecules within a 15 nm × 15 nm × 15 nm box. Both simulations were performed under the NPT ensemble, maintaining a temperature of 298.15 K and a pressure of 1 bar. The first simulation ran for 2 ns, while the second was extended to 100 ns. A time step of 2 fs was employed, and prior to MD simulations, energy minimization was performed using the steepest descent method until the maximum force reached below 1 kJ mol^−1^ nm^−1^, with a step size of 0.001 ps. Initial velocities were assigned following the Maxwell–Boltzmann distribution at 300 K. The SPC/E model was used to represent water. Periodic boundary conditions were applied in all directions. The equations of motion were integrated with the leapfrog algorithm. For the NPT simulations, pressure was maintained isotropically at 1 bar using the Berendsen method, while the temperature was controlled using the V-rescale thermostat. Electrostatic interactions were calculated using the Particle-Mesh Ewald (PME) method [35] with fourth-order interpolation and a grid spacing of 1.2 Å. A Lennard–Jones cutoff radius of 1.0 Å was used for van der Waals interactions.

Finite element simulations. Finite element simulations were conducted using COMSOL Multiphysics 6.2, employing the “Cubic current distribution” physics interface to model both the current density and ion concentration fields. The ion concentration dynamics were governed by Fick’s first law of diffusion, while ion migration followed the Nernst–Planck equation. The simulation models simplified the geometry of the system, featuring two electrodes of 6 μm in length and separated by 4 μm. To represent the zinc anode surface morphology, three semi-circular protrusions with a radius of 4 μm were placed on the anode surface. The electrolyte was modeled as the primary medium for ion transport. Boundary conditions were applied to the anode and cathode potentials, with the anode held at a zero-potential boundary and the cathode potential defined by the polarization voltage measured experimentally. The initial concentration of Zn^2+^ was set at 2 M, and the diffusion coefficients for Zn^2+^ in the electrolyte were assigned values of 1.0 × 10^–17^ and 3.0 × 10^–16^ m^2^ s^−1^. The exchange current density for the battery was set to 1 mA cm^−2^, and the system was maintained at a temperature of 298 K.

## Results and Discussion

### Bionic Bulk Helical and Interfacial Bilayered Self-Assembly

**Fig. 1:**
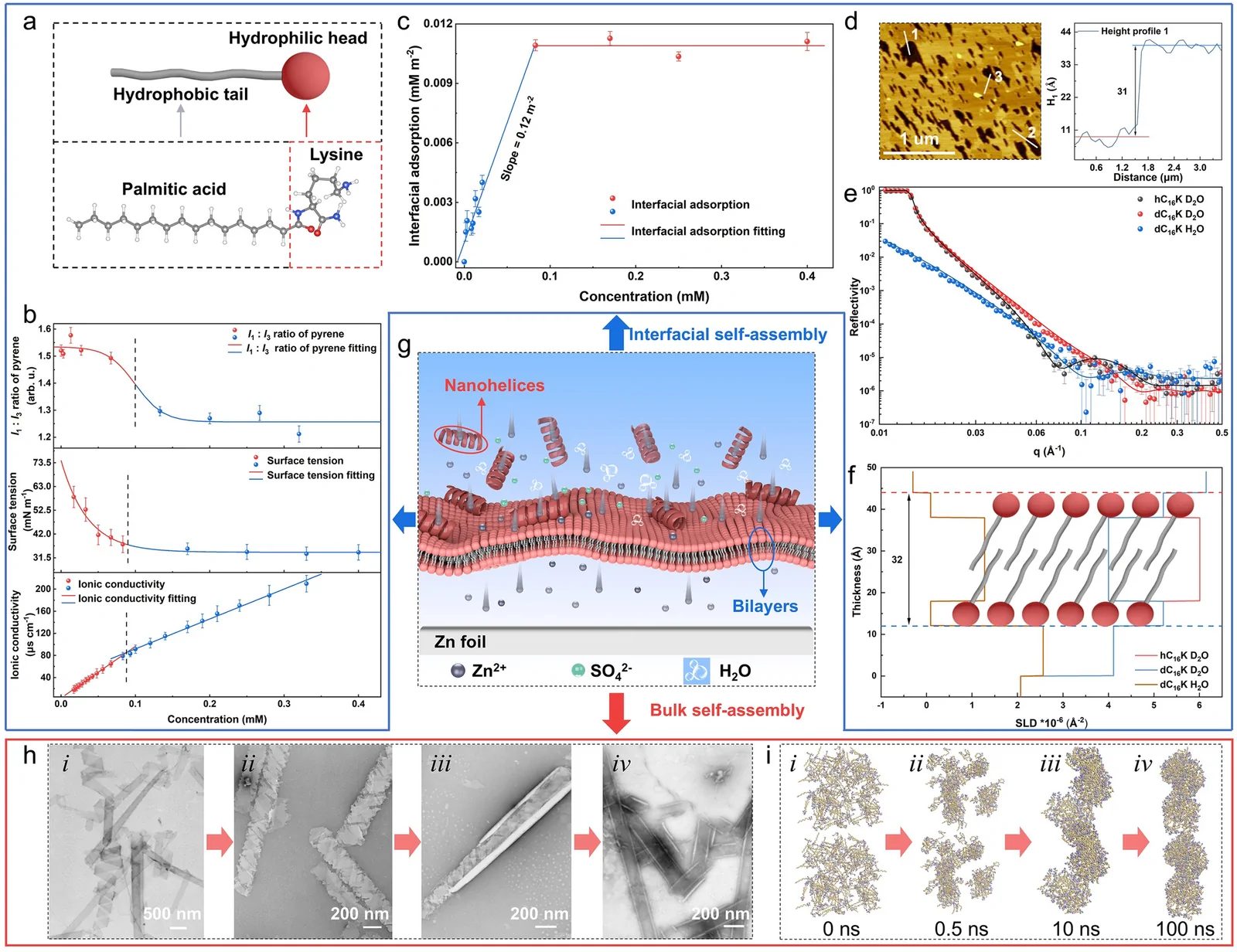
Bionic bulkily nanohelical and interfacially bilayered self-assembly of the minimalistic lipopeptide. **a** Molecular structure of the designed C_16_K lipopeptide with a palmitic acid as the hydrophobic tail and a positively charged K residue as the hydrophilic head. **b** CMC determination of the C_16_K lipopeptide using three strategies. From top to bottom: pyrene probe fluorescence spectroscopy, surface tension-concentration curve, and concentration-dependent ionic conductivity evolution. **c** Adsorption isotherm of the C_16_K lipopeptide after adsorption for 30 min at the solid–liquid interface. **d** AFM characterization of the topological morphology of the self-assembled membrane at 0.10 mM of C_16_K. The right panel shows the corresponding height profile along Line #1 in the left image. **e** NR curves of the interfacially self-assembled bilayer of C_16_K. **f** Schematic cartoon showing the configuration of the self-assembled bilayer achieved from the NR fittings. **g** Schematic cartoon showing the bionic nanohelical and bilayered self-assembly of the minimalistic lipopeptide. **h** TEM images showing the concentration-dependent bulk self-assembly evolution of C_16_K. **i** CGMD simulations showing the bulk self-assembly kinetics of C_16_K into nanohelices

In order to achieve synergistic reinforcement in the metal-ion batteries, a bioinspired lipopeptide was designed. Due to the intrinsic amphiphilic nature, lipopeptides can self-assemble into supramolecular architectures in the solution and adsorb on the interface to form dynamic cellular membrane-like bilayers [25]. In this regard, a minimalistic lipopeptide C_16_K, composed of a palmitic acid as the hydrophobic tail to trigger the self-assembly and a positively charged lysine (K) as the hydrophilic head exhibiting a strong affinity toward negatively charged metallic complexes, was synthesized through the Fmoc solid-phase peptide synthesis technology (Figs. 1a, S2, and S3) [28].

The Fourier transform infrared spectroscopy (FTIR) and energy-dispersive X-ray spectra (EDS) characterizations confirmed the adsorption of the minimalistic lipopeptide molecules on metallic foil, showing the characteristic peaks and elemental compositions of peptidic entities, respectively (Figs. S4 and S5, Table S1). This could be further verified by high-resolution X-ray photoelectron spectroscopy (XPS) measurements, showing a distinct N 1*s* peak and a noticeable positive shift of the electron binding energies of the metallic atoms upon introducing C_16_K (Fig. S6) [36]. In order to in-detail investigate the self-assembly behaviors, the critical micelle concentration (CMC) of the lipopeptide was firstly determined to be 0.09–0.10 mM using the pyrene probe fluorescence, surface tension, and specific conductivity methodologies, respectively (Fig. 1b) [37]. This demonstrates that a concentration of no less than 0.10 mM was required to synergistically achieve bulk and interfacial self-assembly. Therefore, in the later investigations, 0.10 mM was assumed. Notably, the smooth evolution curves of the *I*_*1*_/*I*_*3*_ ratio (from 1.53 to 1.26) and surface tension (from 73.4 to 34.0 mN m^−1^) suggested that C_16_K presented characteristic self-assembly behaviors of amphiphilic building blocks, organizing into supramolecular architectures with distinct hydrophobic/hydrophilic sections in the solution and an extensive membrane at the interface [28].

Subsequently, the dynamic interfacial behaviors of the minimalistic lipopeptide were characterized by spectroscopic ellipsometry (SE) (Fig. S7), revealing a typical Langmuir adsorption with the deposition amount evolved from a linearly rapid rising (with a calculated slope of 0.12 m^−2^) to a stable equilibrium plateau when the concentration reached and exceeded the CMC in the adsorption isotherm profile (Fig. 1c) [29]. At this point, the maximum adsorption weight of C_16_K was measured to be 4.22 mg cm^–2^, with a calculated average thickness of approximately 3.0 ± 0.1 nm. Furthermore, the atomic force microscopy (AFM) characterizations visually verified the adsorbed membrane, showing the amplified occupation area with increasing the concentrations (Fig. S8). Upon surpassing the CMC, a uniform and densely packed membrane with the measured average thickness of 3.3 ± 0.2 nm dominated the views, consistent with the SE findings (Figs. 1d and S9). Furthermore, the neutron scattering (NR) measurements demonstrated that the membrane exhibited a sandwich-like structure (Fig. 1e) [38], composed of two K-residue layers at two ends (exterior) and an interdigitated palmitic alkyl chain phase in the middle (interior) (Fig. 1f; Table S2), with an apparent total thickness of 3.2 ± 0.1 nm, consistent with the previously obtained results and suggestive of a bilayered alignment. Therefore, it can be concluded that the minimalistic lipopeptide self-assembled into cellular membrane-like bilayers (Fig. 1g) at the liquid–solid interface, offering ultrathin, self-adaptive, and dynamic solid electrolyte interface to impede dendrites growth or HER side effects [24].

Similar to state-of-the-art amphiphilic building blocks, C_16_K molecules could self-assemble into supramolecular architectures when the concentration reached or exceeded CMC. Specifically, nanohelical structures were achieved at the concentration of 0.10 mM under the transmission electron microscopy (TEM) observations (Fig. 1h, *i*). With concentration increasing, the nanohelices further ripened to denser (0.20 mM) and coalesced (0.30 mM) nanohelices (Fig. 1h, *ii* and *iii*) and finally evolved into nanotubes with a statistical outer diameter of 266 ± 23 nm and inner diameter of 191 ± 32 nm at 1.0 mM (Figs. 1h, *iv* and S10). Plausibly, the subtle force equilibrium among hydrophobic interactions, electrostatic repulsive interactions, and hydrogen bonding led to the aggregated strands to twist [39]. Furthermore, the coarse-grained molecular dynamics (CGMD) simulations investigated that at the initial beginning of self-assembly, the hydrophobic interactions drove the aggregation of the alkyl chains. While the hydrophilic K headgroups oriented outwards, interacting with the surrounding solvent molecules and forming oligomeric clusters (Fig. 1i, *ii*). Subsequently, these aggregates elongated and started twisting to reduce the torsions, forming the periodic helical conformations (Fig. 1i, *iii*). Over time, the stable nanostructures emerged, culminating in the well-defined nanohelices to mimic the natural ion channels (Fig. 1i, *iv*). Based on the combinational theoretical and experimental findings, the self-assembly procedure of the designed lipopeptide could be depicted, where the metalphilic K residues were distributed outside the hydrophobic scaffolds, thus establishing ion-channel like configurations (Figs. 1g and S11). This laid the basis of enhancing metal-ion transport in the electrolyte [40].

### Synergistic Enhancement of Metal ion Transport and Interfacial Protection

Combining the bulk nanohelical and interfacial bilayered organizations, it was intriguing to study the electrochemical performances of the aqueous metal-ion batteries at the presence of C_16_K. The Raman spectroscopy characterizations demonstrated that the relative proportions of the solvent-separated ion pair (SSIP, [Zn^2+^(H_2_O)_6_·SO_4_^2−^]) at 984 cm^−1^ and the contact ion pair (CIP, [Zn^2+^(H_2_O)_5_·OSO_3_^2−^]) at 994 cm^−1^ remained unchanged at the presence (46.2% *vs*. 53.8%) or in the absence (45.0% *vs*. 55.0%) of C_16_K (Fig. S12) [41]. In addition to the consistent *v*_(O–H)_ vibrations (Fig. S13) and *v*_(_SO_4_^2−^_)_ vibrations (Fig. S14) in the FTIR spectra along with the negligible chemical shift in the ^1^H-NMR spectra (Fig. S15), it could be speculated that C_16_K self-assembly had a minimal impact on the solvation of Zn^2+^ or the hydrogen bonding networks, suggesting that the electrochemical mechanisms underlying the ZIBs did not change upon introducing the lipopeptide.

**Fig. 2:**
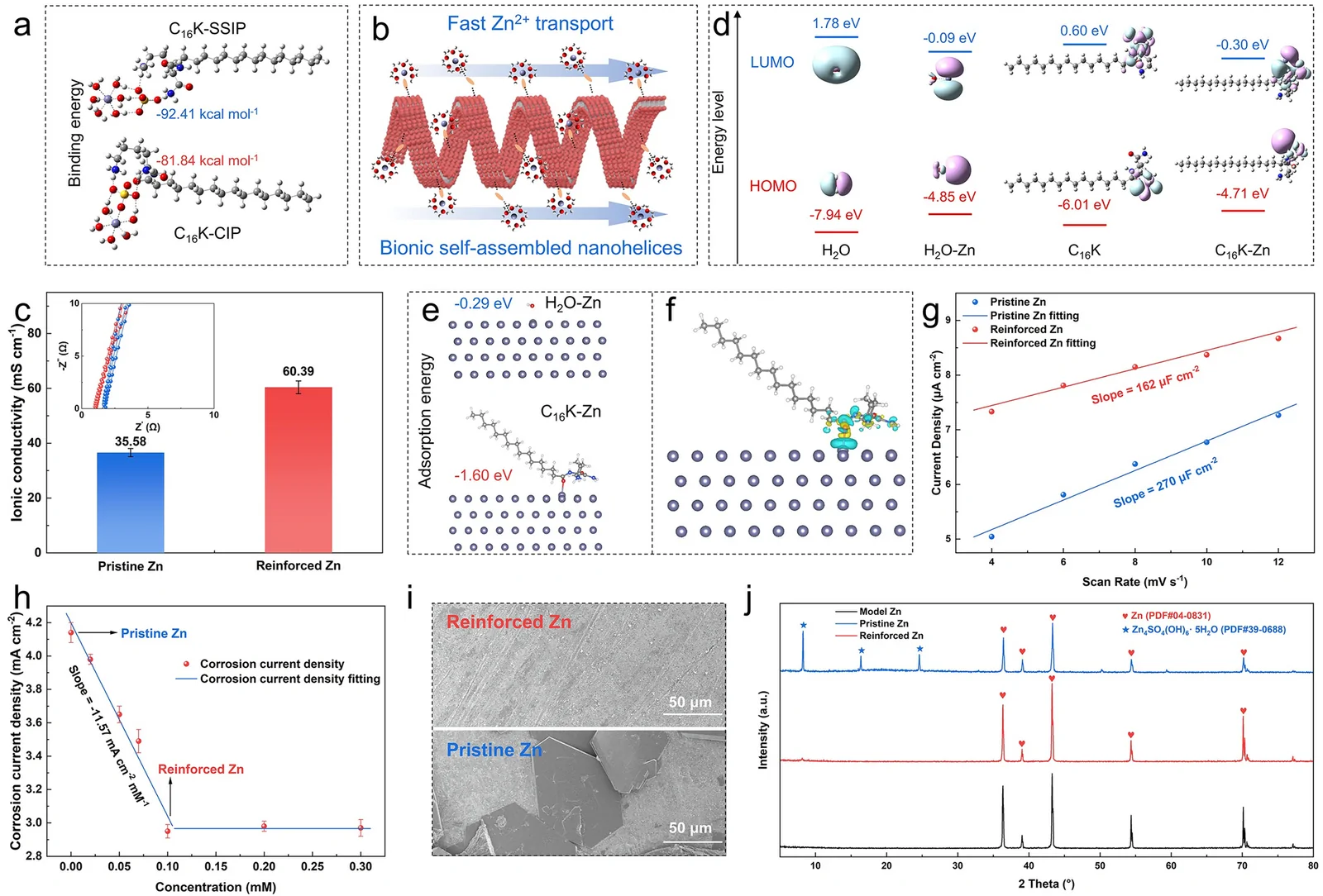
Synergistic metal-ion transport enhancement and electrode protection by lipopeptide self-assembly. **a** Binding energy of C_16_K-SSIP and C_16_K-CIP. **b** Schematic illustration showing the bionic self-assembled nanohelices facilitating rapid metal-ion transport. **c** Ionic conductivity statistics of the native ZnSO_4_- and C_16_K-containing electrolytes. **d** Molecular orbital energies of H_2_O, H_2_O-Zn, C_16_K, and C_16_K-Zn. **e** Adsorption energies of H_2_O and C_16_K molecules on Zn (002) plane, respectively. **f** Charge density distribution map of C_16_K molecule on the Zn(002) facet. **g** EDL measurements of Zn metal in native ZnSO_4_ and C_16_K-containing electrolytes, respectively. **h** Corrosion current density evolution versus C_16_K concentrations in the Zn||Zn symmetric cell. **i, j** SEM images and XRD patterns of the Zn anodes after soaked in native ZnSO_4_ and C_16_K-containing electrolytes for 16 days, respectively

The electrostatic potential (ESP) calculations demonstrated that the electron density of the C_16_K molecule was predominantly localized at the electronegative oxygen and nitrogen atoms, surpassing that of H_2_O (Fig. S16), thereby indicating its high feasibility of coordination with metal ions [24, 36]. Subsequently, the quantum chemistry calculations revealed that the C_16_K molecule exhibited much stronger binding affinities toward SSIP (− 92.41 kcal mol^−1^) and CIP (− 81.84 kcal mol^−1^) (Fig. 2a), compared to its interaction with Zn^2+^ (− 16.95 kcal mol^−1^) and that of H_2_O with Zn^2+^ (− 6.55 kcal mol^−1^) (Fig. S17). Given that the solvation structure of Zn^2+^ remained unchanged, this enhanced interactions plausibly arose from the anion-bridged adsorption of the solvated Zn^2+^ onto the self-assembled nanohelices. Therefore, upon applying an external electric field, the metal ions could migrate along the nanohelical architectures and facilitate mobility (Fig. 2b) [15, 23]. This speculation was validated by the ionic conductivity measurements, during which the lipopeptide-containing electrolyte exhibited a quite high ionic conductivity up to 60.39 mS cm^−1^, approximately twice of that in the native ZnSO_4_ case (35.58 mS cm^−1^) (Fig. 2c), thus confirming the transport-enhancing effect of the bionic supramolecular nanohelices.

Subsequently, the effect of the interfacial self-assembly was investigated. Specifically, the molecular orbital analysis revealed that C_16_K exhibited enhanced electron transfer capability compared to H_2_O due to the lower lowest unoccupied molecular orbital (LUMO) and higher highest occupied molecular orbital (HOMO) energies (Fig. 2d). The density functional theory (DFT) calculations demonstrated that C_16_K had a stronger adsorption energy (-1.60 eV) than H_2_O (-0.29 eV) on Zn(002) plane (Fig. 2e), through the electron transfer between the amide O atom and Zn surface (Fig. 2f). Obviously, the molecules employed tilted configurations on the metallic surface, in addition to the interdigital nature of the palmitoyl chains, underlying the foundation why the height of the interfacial self-assembly (~ 3.3 nm as discussed above) was lower than twice of two lipopeptide molecule lengths (~ 5.4 nm) [24, 42]. This endowed a smaller contact angle on the Zn surface (79°) of the C_16_K-contained electrolyte compared to that of the native ZnSO_4_ solution (99°) (Fig. S18) along with the decreased zeta potential of the Zn powder from -5.9 to -2.5 mV (Fig. S19), thus suggesting the dynamic, robust interfacial self-assembly of C_16_K on the electrolyte–electrode interface [14, 24]. Moreover, the cyclic voltammetry (CV) characterizations via the Zn||Zn symmetric cells indicated that the electrical double-layer (EDL) capacitance attenuated to 162 from 270 μF cm^−2^ upon introducing C_16_K (Figs. 2g and S20), demonstrating that the ultrathin bilayers penetrated the inner Helmholtz plane (IHP), isolating H_2_O molecules away as well as promoting Zn^2+^ desolvation and suppressing corrosions from the Zn surface. Therefore, the effect on corrosion behaviors of C_16_K interfacial self-assembly was further evaluated using the Tafel experiments. The results demonstrated that the corrosion was gradually mitigated upon introducing C_16_K, showing a linearly fitted remittance slope of − 11.57 mA cm^−2^ mM^−1^ from original 4.14 mA cm^−2^ in the native ZnSO_4_ solution to 2.95 mA cm^−2^ when the concentration of C_16_K reached CMC (Figs. 2h and S21). Notably, the corrosion current density kept constant after CMC regardless of concentrations, probably attributed to the overspreading of the supramolecular bilayer, thereby minimizing the corrosions. In addition to the increased corrosion potential (from − 1.007 to − 1.004 V) (Fig. S21) and the decreased initial HER potential (from − 1.897 to − 1.934 V) (Fig. S22), it could be supposed that the by-products formed on Zn anodes were efficiently suppressed upon C_16_K interfacial self-assembly. In fact, the SEM characterizations demonstrated that the Zn electrode maintained a smooth surface with negligible XRD signals of by-products after 16-day immersion at the presence of C_16_K (Fig. 2i, top panel), in contrast to the randomly oriented hexagonal by-products in the control (Fig. 2i, bottom panel), which presented characteristic diffraction peaks (located at 8.3°, 16.4°, and 24.6°) corresponding to the by-products of Zn_4_SO_4_(OH)_6_·5H_2_O (ZHS, PDF#39-0688) (Fig. 2j) [43]. This again highlighted the high-efficiency suppression of corrosions or side reactions toward metallic electrode of C_16_K interfacial self-assembly.

### Enhanced Reaction Kinetics and Uniform Metal Deposition

**Fig. 3:**
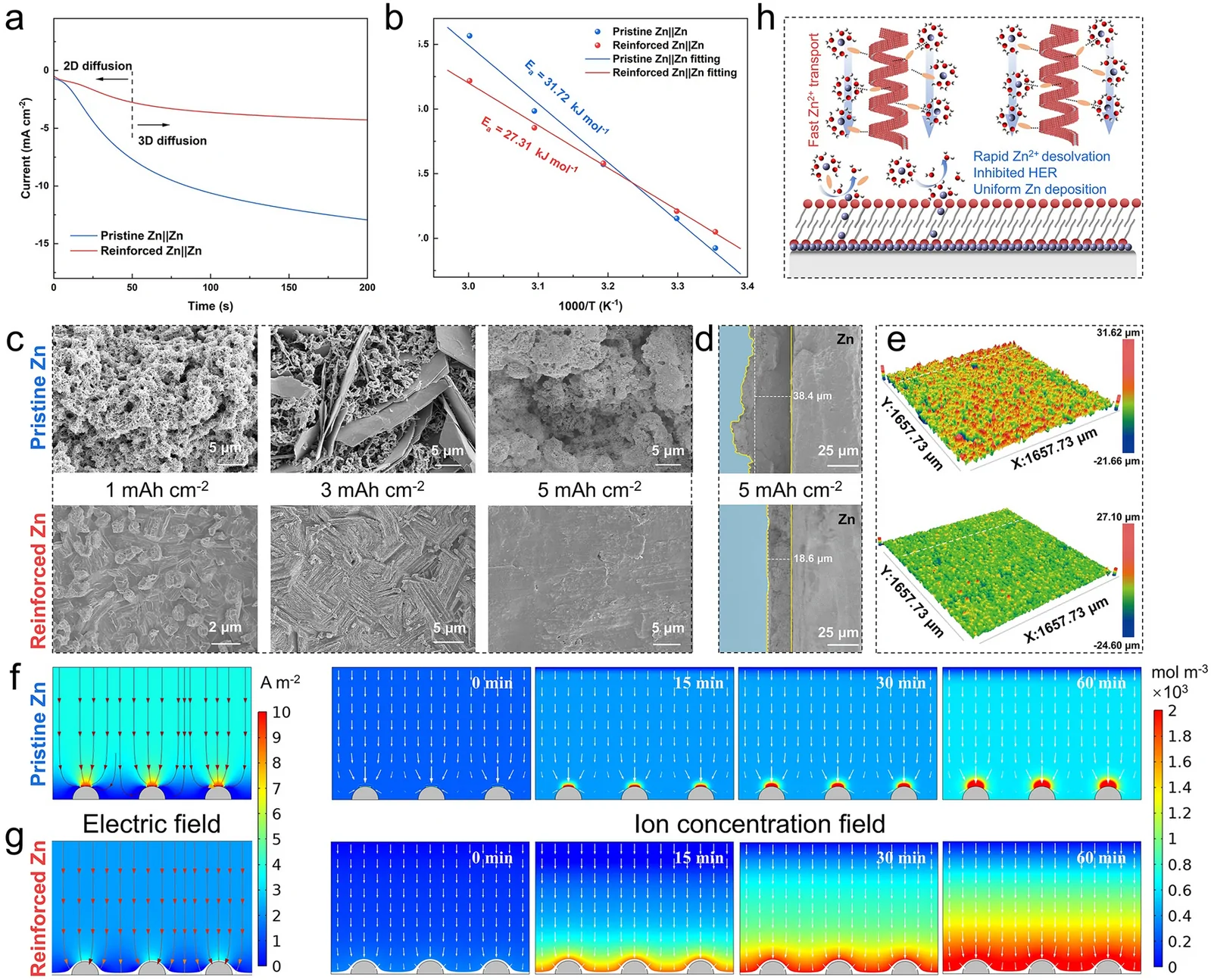
Enhanced metal-ion plating/stripping kinetics and uniform deposition induced by synergistic self-assembly. **a, b** CA tests of the Zn||Zn symmetric cells at a constant voltage of -150 mV and calculated desolvation energy of Zn^2+^ in the absence or at the presence of C_16_K, respectively. **c** SEM images of Zn plating at 1, 3, and 5 mA cm^−2^ for 1 h on the Cu substrate (top panel) in the absence or (bottom panel) at the presence of C_16_K, respectively. **d, e** Cross-sectional SEM and corresponding 3D white-light interferometry images of the Cu substrate after plating at 5 mAh cm^−2^ for 1 h of Zn (top panel) in the absence or (bottom panel) at the presence of C_16_K, respectively. **f, g** Electric field and ion concentration field simulation (top panel) in the absence or (bottom panel) at the presence of C_16_K, respectively. **h** Schematic illustration showing the mechanism underlying synergistic reinforcement on metal-ion plating/stripping kinetics as well as uniform deposition of bulk and interfacial self-assembly

The mean-square displacement (MSD) analysis from the CGMD simulations revealed that the diffusion coefficient of Zn^2+^ along the C_16_K self-assembled bilayers was higher than those in other directions (Fig. S23), indicating a preferential transport direction upon introducing the bionic supramolecular membrane. And the Zn plating voltage profile demonstrated that the Zn||Cu asymmetric cell at the presence of the lipopeptide self-assemblies exhibited smaller nucleation overpotential (${\eta }_{n}$ = 97.3 mV) and growth overpotential (${\eta }_{g}$ = 24.7 mV) at 1.0 mA cm^−2^ than the pristine Zn||Cu cell (${\eta }_{n}$ = 134.4 mV, ${\eta }_{g}$ = 32.8 mV) (Fig. S24), indicating a reduced energetic barrier for Zn nucleation [44]. The electrochemical impedance spectroscopy (EIS) measurements discovered a lower charge-transfer resistance (*R*_*ct*_) (327.5 Ω) at the presence of the lipopeptide self-assembly contrast to that in the native ZnSO_4_ solution (746.8 Ω), indicating the more efficient charge transfer upon introducing the lipopeptide (Fig. S25). Together with the improvement of the Zn^2+^ transference number, which increased to 0.34 for the reinforced Zn||Zn cell with 0.10 mM C_16_K from 0.18 for the pristine counterpart and then remained nearly constant regardless of the C_16_K concentrations (Fig. S26 and Table S3), these results substantiated that the synergistic bulk nanohelical and interfacial bilayer self-assembly promoted Zn^2+^ ion transport, mitigated concentration polarization and SO_4_^2−^ accumulation at the anode/electrolyte interface, thus dramatically accelerating the reaction kinetics of Zn^2+^ plating/stripping. Therefore, the chronoamperometry (CA) measurements taken using the symmetric cells under an external overpotential of – 150 mV demonstrated that the reinforced Zn||Zn system showed a stable current response with a lower current density after 50 s, in contrast to the continuous attenuation over 200-s plating process in the control case (Fig. 3a), illustrating the suppressed 2D accumulation and stimulated 3D diffusion for uniform Zn^2+^ deposition.

The primary bottleneck in charge migration stems from the metal-ion desolvation process nearby the electrode/electrolyte interface [45]. In this regard, the desolvation energy barrier of the Zn^2+^ transference was quantified using the Arrhenius activation energy (*E*_*a*_). Specifically, the *E*_*a*_ for the reinforced Zn||Zn system was calculated to be 27.3 kJ mol^−1^, lower than that in the native ZnSO_4_ solution (31.7 kJ mol^−1^) (Figs. 3b and S27). This remarkable reduction suggests that the C_16_K self-assemblies interacted efficiently with the hydrated Zn^2+^, facilitating interfacial desolvation and thereby enhancing charge transfer and deposition kinetics [46–48]. Furthermore, the CV measurements of the C_16_K-contained Zn||Cu asymmetric cell revealed a higher redox peak and a reduced overpotential compared to the pristine counterpart (Fig. S28), indicating the improved electrochemical reversibility upon synergistic reinforcing effect. In addition, the exchange current density in the C_16_K-contained Zn||Zn symmetric cell (2.22 mA cm^−2^) notably exceeded that in the pristine Zn||Zn case (1.95 mA cm^−2^) (Fig. S29), further substantiating the accelerated Zn^2+^ plating/stripping kinetics triggered by lipopeptide synergistic self-assembly.

Rationally, the enhanced reaction kinetics by the synergistic effects infers uniform and sustainable plating/stripping. In this regard, the SEM characterizations demonstrated that as the deposition capacity increased from 1.0 to 5.0 mAh cm^−2^ under different current densities, disordered Zn clusters emerged and gradually evolved into moss and flake-like structures in the pristine ZnSO_4_ solution, ultimately ripening into loosely bound by-products prone to detachment during plating (Fig. 3c, top panel; Fig. S30a). By contrast, the Zn deposition at the presence of C_16_K exhibited a different behavior, where the initially small grains expanded slightly, forming well-aligned hexagonal sheets that eventually developed into a uniform, compact, and dendrite-free layer (Fig. 3c, bottom panel; Fig. S30b), with a measured thickness of only 18.6 ± 0.6 μm, around half of that (38.4 ± 6.7 μm) formed in the control case (Fig. 3d) and closer to the theoretical thickness (~ 8.5 μm) of a Zn deposit at an areal capacity of 5 mAh cm^−2^. Correspondingly, the 3D white-light interferometry images of the Zn electrode displayed a smooth and uniform deposition surface (S_a_ = 3.75 μm and S_q_ = 4.80 μm) with negligible height fluctuations (S_z_ = 52.69 μm) under the deposition capacity of 5 mAh cm^−2^ at the presence of C_16_K, in contrast to the substantially rough surface in the control case (S_a_ = 4.91 μm, S_q_ = 6.30 μm and S_z_ = 53.28 μm) (Figs. 3e and S31). Notably, the uniform Zn^2+^ deposition process and suppressed side reactions could be visually monitored by in situ optical microscopy characterizations. Specifically, the homogeneous and compact Zn deposition without conspicuous dendrites or bubbles were detected on the reinforced Zn anode during the testing period (40 min), in contrast to the uneven and moss-like protrusions which erupted and rapidly accumulated into loose dendrites along with hydrogen bubbles in less than 20 min in the control system (Fig. S32).

To further elucidate the electric field distribution and ion concentration field during Zn plating, the COMSOL Multiphysics simulations were conducted. Specifically, in the pristine ZnSO_4_ electrolyte case, the charge accumulation at the protruding sites led to locally elevated current densities. As a result, the metal ions preferentially aggregated at these protrusions, while it exhibited minimal presence at the flat regions (Fig. 3f). By contrast, the interfacial self-assembled bilayer mitigated the localized electric field intensification at the protrusions, promoting a uniform field distribution near the surface. Consequently, Zn^2+^ concentration remained evenly dispersed above the reinforced anode, thus effectively inhibiting dendrite growth (Fig. 3g).

Therefore, the synergistic improvement of ion transport and interfacial stability could effectively enhance the reaction kinetics and regulate metal-ion deposition (Figs. 3h and S33). In the bulk solution, the lipopeptide molecules self-assembled into bionic supramolecular nanohelices and their functional groups interacted with the solvated Zn^2+^, promoting ion transport and increasing ionic conductivities. At the electrode interface, the self-assembled dynamic, adaptive bilayer facilitated Zn^2+^ interfacial desolvation, accelerated the ion migration, and improved the reaction kinetics [15]. In addition, the bilayer could serve as a protective barrier to regulate the ion flux and electric field distribution, which prevented direct water contact with the anode and inhibited continuous corrosion or hydrogen evolution, thus endorsing uniform metal-ion deposition while impeding dendrite growth or byproduct formation [24].

### Improved Electrochemical Stability of the Metal Electrode

**Fig. 4:**
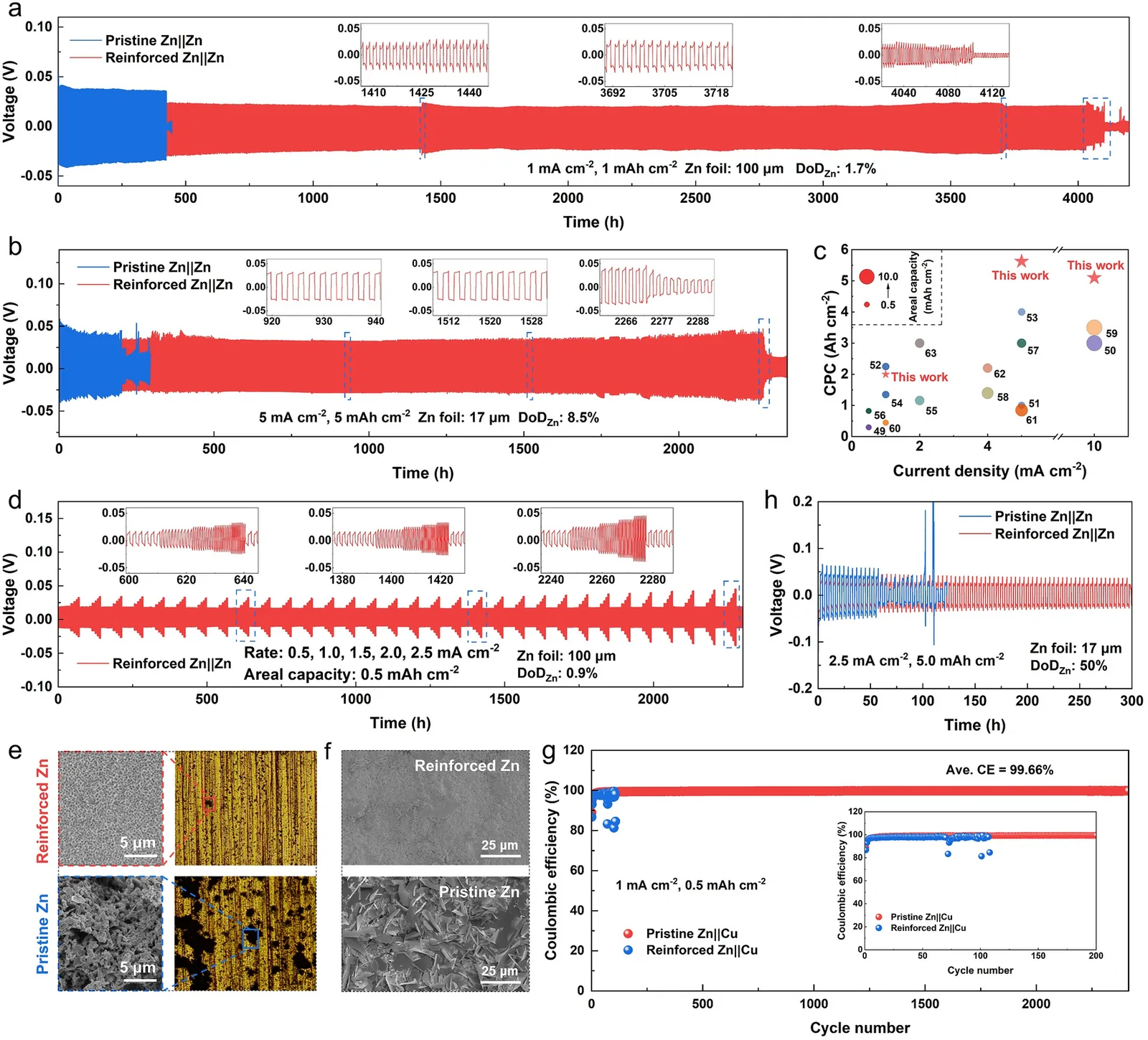
Improved electrochemical performance of metal anodes through the synergistic self-assembly strategy. **a, b** Voltage profiles of the pristine and reinforced Zn||Zn symmetric cells at current densities of 1 and 5 mA cm^−2^, respectively. The insets show locally magnified voltage profiles at different cycling times. **c** Comparison of the electrochemical performance in diverse Zn||Zn symmetric cells with different treatment strategies. **d** Long-term rate performance of the pristine and reinforced Zn||Zn symmetric cells at varied current densities from 0.5 to 2.5 mA cm^−2^. The inset shows locally magnified voltage profiles at different cycling times. **e** CLSM along with SEM images corresponding to the rectangular frameworks of the pristine and reinforced Zn anodes after cycling for 10 cycles at 1 mA cm^−2^ for 1 mAh cm^−2^. **f** SEM images of the pristine and reinforced Zn anodes after 50 cycles at 1 mA cm^−2^ for 1 mAh cm^−2^, respectively. **g** CE comparison of the pristine and reinforced Zn||Cu asymmetric cells at 1 mA cm^−1^ for 0.5 mAh cm^−2^. The inset presents an enlarged view of the initial 200 cycles. **h** Cycling performance of the pristine and reinforced Zn||Zn symmetric cells with a high Zn utilization rate of 50% DoD_Zn_

Plausibly, under the synergistic effects mentioned above, the bionic self-assembly-reinforced ZIBs may present enhanced performances. Accordingly, the reversibility of the metallic anodes was investigated using the Zn||Zn symmetric cells. Specifically, the reinforced system exhibited an ultralong lifespan exceeding 4000 h at 1 mA cm^−2^ for 1 mAh cm^−2^, approximately 40 times longer than that tested in the pristine ZnSO_4_ electrolyte (Fig. 4a). Even under a higher current density at 5 mA cm^−2^ for 5 mAh cm^−2^, the reinforced cell still maintained a prolonged cycle life of over 2250 h (Fig. 4b), achieving an exceptional cumulative plating capacity (CPC) of up to 5.625 Ah cm^−2^, intensely higher than most of the previously reported cells employing various treatment strategies including electrolyte additives [49–58], gel electrolytes [59, 60], anode coatings [61, 62], and functional separators [63] (Fig. 4c, Table S4). Especially, the cell sustained a stable cycling for over 1020 h at 10 mA cm^−2^ for 5 mAh cm^−2^ (Fig. S34), notably outperforming the counterpart based on the pristine ZnSO_4_ solution. These results underscored the critical role of synergistic self-assembly in suppressing side reactions and mitigating dendrite growth, thereby ensuring highly reversible Zn plating/stripping. Moreover, the reinforced Zn||Zn symmetric cells exhibited an outstanding rate performance with lower voltage hysteresis (Fig. S29), maintaining an ultralong lifespan of over 2300 h as the current density repeatedly increased from 0.5 to 2.5 mA cm^−2^ (Fig. 4d). In addition, FTIR and XPS of cycled Zn electrodes showed unchanged characteristic vibrational bands and N 1*s* binding energy of C_16_K (Fig. S35), confirming its molecular integrity. This further highlighted that the synergistic effect of bionic supramolecular nanohelices and bilayers was pivotal for improving the cycling reversibility in metal-ion batteries [64, 65].

The confocal laser scanning microscopy (CLSM) characterizations demonstrated that the Zn anodes in the reinforced cells still exhibited a smooth and dendrite-free surface after 10 cycles at 1 mA cm^−2^ for 1 mAh cm^−2^, showing a dense and uniform layered morphology in the SEM images (Fig. 4e, top panel). As a control, the anode based on the pristine ZnSO_4_ solution revealed extensively distributed prominent dendrites with loosely packed and disordered structures under the same conditions, which are well-known to severely deteriorate battery lifespan (Fig. 4e, bottom panel). Furthermore, even after 50 cycles the reinforced Zn anodes still maintained a flat and uniform deposition morphology without detectable by-products (Fig. 4f, top panel), contrast to the substantial accumulation of ZHS by-products in the control case (Fig. 4f, bottom panel; Fig. S36) [43]. Therefore, the symmetric cell tests again substantiated that the synergistic effect of the self-assembled nanohelices and bilayers could effectively suppress HER and associated corrosion by facilitating Zn^2+^ transport and preventing water contact.

Furthermore, the coulombic efficiency (CE) measurements demonstrated that the reinforced Zn||Cu asymmetric cell at the presence of C_16_K exhibited an exceptional cycling stability, preserving an average CE of up to 99.66% over 2400 cycles at 1 mA cm^−2^ for 0.5 mAh cm^−2^ (Fig. 4g), along with reversible and steady voltage curves (Fig. S37a). As a control, the device in the absence of lipopeptide experienced severe fluctuations after 70 cycles, and completely failed at 109 cycles (Fig. S37b). Furthermore, under a higher current density at 5 mA cm^−2^ for 2.5 mAh cm^−2^, the reinforced cell enabled stable cycling for over 400 cycles with an impressive CE of 99.76% (Figs. S38 and S39a). By contrast, the pristine Zn||Cu asymmetric cell suffered from premature CE degradation and short-circuit failure at only 76 cycles (Figs. S38 and S39b), thus underscoring the high-efficiency of the synergistic effect in suppressing side reactions or dendrite growth. Moreover, the Zn||Zn symmetric cells using ultrathin Zn foils (17 μm in thickness, Fig. S40) at a high Zn utilization rate of 50% depth of discharge (DoD_Zn_) characterizations demonstrated that the C_16_K-based device sustained a long-term cycling performance for nearly 300 h with uniform plating/stripping, fivefold longer than that of the pristine counterpart which exhibited a rapid voltage drop after approximately only 60 h (Figs. 4h and S41). This highlighted the reliability of the synergistically improved ion transport and interfacial stability for sustainable plating/stripping in metal-ion batteries, endorsing the promising potential for practical applications in wearable electronics [64, 65].

### Reinforced Electrochemical Performance of the Full Cells

**Fig. 5:**
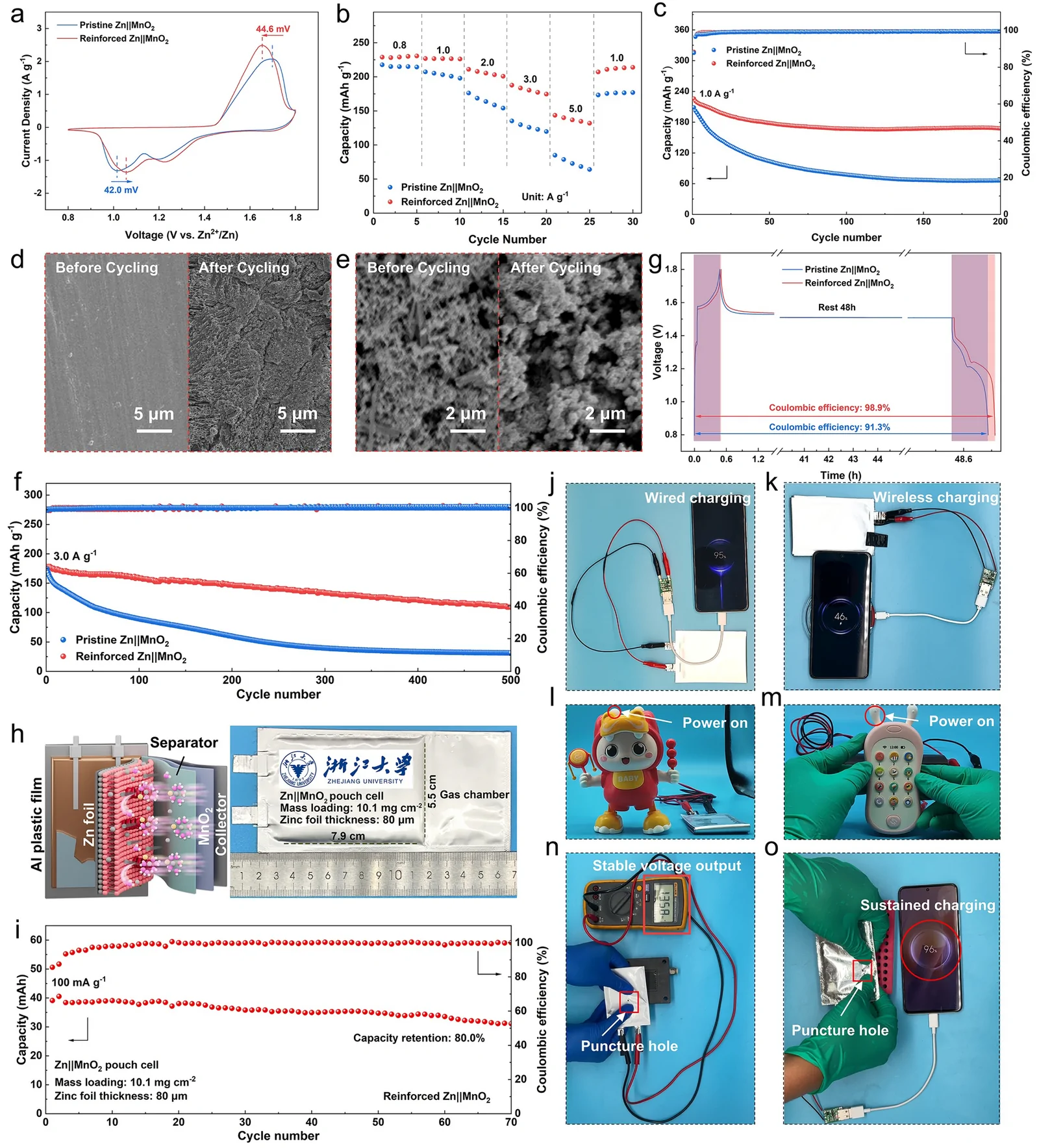
Electrochemical performance of the full cells reinforced by the synergistic self-assembly strategy. **a-c** CV profiles with a voltage range of 0.8–1.8 V at a scan rate of 0.5 mV s^−1^, rate performance, and long-term cycling performance at 1.0 A g^−1^ of the Zn||MnO_2_ full cells in the absence or at the presence of C_16_K self-assemblies, respectively. **d, e** SEM images showing the electrode morphologies of the Zn||MnO_2_ full cells (left panel) before cycling and (right panel) after cycling for 50 cycles at 1.0 A g^−1^, respectively. **f, g** Long-term cycling performance at 3.0 A g^−1^ and self-discharge behavior of the pristine and reinforced Zn||MnO_2_ full cells, respectively. **h** Schematic illustration and photographical image of a reinforced Zn||MnO_2_ pouch cell, respectively. **i** Cycling performance of the reinforced Zn||MnO_2_ pouch cell at 100 mA g^−1^. The mass loading is 10.1 mg cm^−2^; the thickness of the employed zinc foil is 80 μm. **j-m** Photographic pictures showing the reinforced Zn||MnO_2_ pouch cell charging smartphones via the wired and wireless methods, powering children’s toy doll and toy phone, respectively. **n, o** Photographic pictures showing the reinforced Zn||MnO_2_ pouch cell maintaining a stable voltage output and sustaining smartphone charging even suffering mechanical puncture, respectively

Based on the results from the symmetric cells, the Zn||MnO_2_ button full cells were further developed using needle-like β-MnO_2_ as the cathode (the β-MnO_2_ was synthesized via a hydrothermal method according to the previous report (Fig. S42)) [36]. To mitigate Mn^2+^ dissolution during cycling, MnSO_4_ at a concentration of 0.1 M was incorporated into the electrolyte. The CV curves demonstrated that the Zn||MnO_2_ cells presented similar and typically reversible redox peaks (Fig. 5a), inferring that the basic charge/discharge processes remained unaffected after introducing MnSO_4_. Especially, the CV curves of the reinforced full cell displayed a decreased oxidation potential by 44.6 mV, an enhanced reduction potential by 42.0 mV and an enhanced redox current relative to the native counterpart (Fig. 5a), along with a more stable discharge plateau accompanied by the lower voltage hysteresis (Fig. S43), suggesting the accelerated reaction kinetics and reduced electrochemical polarization driven by the synergistic effect of C_16_K self-assemblies. Furthermore, the GITT measurements were conducted to evaluate the ion-diffusion coefficients (${D}_{Zn}^{2+}$) throughout the charge–discharge process (Fig. S44). As expected, the reinforced Zn||MnO_2_ cell showed higher ${D}_{Zn}^{2+}$ values (~ 10^–13^ to 10^–11^ cm^2^ s^−1^) than the pristine counterpart, further confirming the improved ion diffusion kinetics.

The rate stability of the full cells was then evaluated. Specifically, the reinforced battery delivered high average discharge specific capacities of 229.3, 226.6, 205.7, 180.7, and 137.4 mAh g^−1^ at 0.8, 1.0, 2.0, 3.0, and 5.0 A g^−1^, respectively, surpassing those of the unprocessed counterpart (Fig. 5b). Notably, the reinforced cell maintained a high capacity of 167.9 mAh g^−1^ after 200 cycles at 1.0 A g^−1^, achieving 75% of the capacity retention and dramatically outperforming the pristine Zn||MnO_2_ case of only 32% (Fig. 5c). In addition, the SEM characterizations established that the electrode surfaces in the reinforced system still retained dendrite-free morphologies after 50 cycles at 1.0 A g^−1^ (Figs. 5d, e), in contrast to the extensive dendrites and sheet-like by-products in the unprocessed counterpart (Fig. S45) [43], thus visually confirming the electrochemical analyses.

This allowed us to further test the electrochemical performances of the metal-ion batteries in higher yields. Specifically, under a higher current density of 3.0 A g^−1^, the reinforced Zn||MnO_2_ cells still kept 61% capacity retention even after 500 cycles, whereas the pristine Zn||MnO_2_ cell suffered from severe capacity fading, with only 18% capacity retention under the same conditions (Fig. 5f). In fact, a similar trend was also observed in the case of even a higher current density of 5.0 A g^−1^ (Fig. S46). Furthermore, the self-discharge tests demonstrated that after being charged to 1.8 V and rested for 48 h before discharging to 0.8 V, the reinforced Zn||MnO_2_ full cells retained 98.9% of its initial charge capacity, surpassing 91.3% retention observed in the untreated case (Fig. 5g). In addition, the presence of C_16_K after high cathode potential treatments was confirmed by MS and XPS analysis (Figs. S47 and S48), thus further highlighting the effect of the synergistic reinforcement of bionic self-assembly in sustaining prolonged and stable cycling performance of the metal-ion batteries.

To evaluate the universality of the synergistic reinforcement of ion transport and interfacial stability for sustainable plating/stripping in metal-ion batteries, (NH_4_)_2_V_4_O_9_ was then employed as an alternative cathode material (Fig. S49) [66]. Similarly, the CV characterizations demonstrated that the C_16_K self-assembly-reinforced Zn||(NH_4_)_2_V_4_O_9_ full cells presented a reduced potential gap between the oxidation and reduction peaks contrast to the untreated counterpart, coupled with a lower *R*_*ct*_ of 211.5 Ω (Fig. S50), proposing the enhanced charge-transfer kinetics. This endorsed the higher specific capacities of the Zn||(NH_4_)_2_V_4_O_9_ full cells, achieving a large and reversible capacity up to 263.5 mAh g^−1^ even at a high current density of 5.0 A g^−1^ (Fig. S51) and a superior cycling stability with retaining 70% of its initial capacity even after 500 cycles at 2.0 A g^−1^ (Fig. S52). And when the current density increased to 5.0 A g^−1^, the reinforced cell still exhibited a sustainable stability with an impressive 84% capacity retention after 1000 cycles, notably outperforming 50% retention observed in the control (Fig. S53) and competitive to recently reported rechargeable zinc-ion batteries (Table S5).

These behaviors highlighted the application potentials of the synergistic reinforcement strategy in practical energy storage field and wearable electronics. In this regard, the pouch cells of large capacities were developed using the C_16_K self-assembly-reinforced ZIBs. Specifically, the Zn||MnO_2_ pouch cell was engineered using a thin Zn foil of 80 μm in thickness with a dimension of 7.9 cm in length and 5.5 cm in width, paired with MnO_2_ cathode with a high mass loading of 10.1 mg cm^−2^. The cell components were organized in a layer-by-layer structure and separated and encapsulated within aluminum (Al) plastic films (Fig. 5h). The assessments demonstrated that the reinforced Zn||MnO_2_ pouch cell exhibited a high initial capacity up to 39.1 mAh at 100 mA g^−1^ and could maintain 80% of its capacity after 70 cycles (Fig. 5i). When at an elevated current density of 125 mA g^−1^, the cell could still retain 72% of its initial capacity of 25.3 mAh after 150 cycles (Fig. S54). These results highlighted the feasibility of the bioinspired reinforced pouch cell in enabling long-term and large-capacity batteries for energy applications.

Therefore, the applicability of the pouch cell in real scenarios was determined. Specifically, the reinforced pouch cell was able to be employed for wired charging of a smart phone (Fig. 5j; Video S1). Furthermore, three interconnected pouch cells endorsed a stable voltage up to 4.05 V (Fig. S55), allowing the wireless smartphone charging (Fig. 5k; Video S2). Next, the pouch cell could also be utilized to power a baby’s toy doll and toy phone (Figs. 5l, m; Videos S3 and S4), highlighting the safety and reliability of the reinforced metal-ion batteries. Notably, contrast to LIBs that pose risks of thermal runaway and leakage, the reinforced pouch cell executes with non-flammable electrolytes and possess inherent biocompatibility and safety. In fact, the puncture tests verified that once being pierced, the reinforced pouch cell could still keep constant energy output in a benign manner (Figs. 5n, o; Videos S5 and S6). These characteristics allowed the reinforced batteries suitable for applications requiring rigorous safety standards such as wearable electronics, where stability, eco-friendliness, and user protection are of paramount importance [20].

### Application of the Reinforced Metal-ion Batteries in Wearable Electronics

**Fig. 6:**
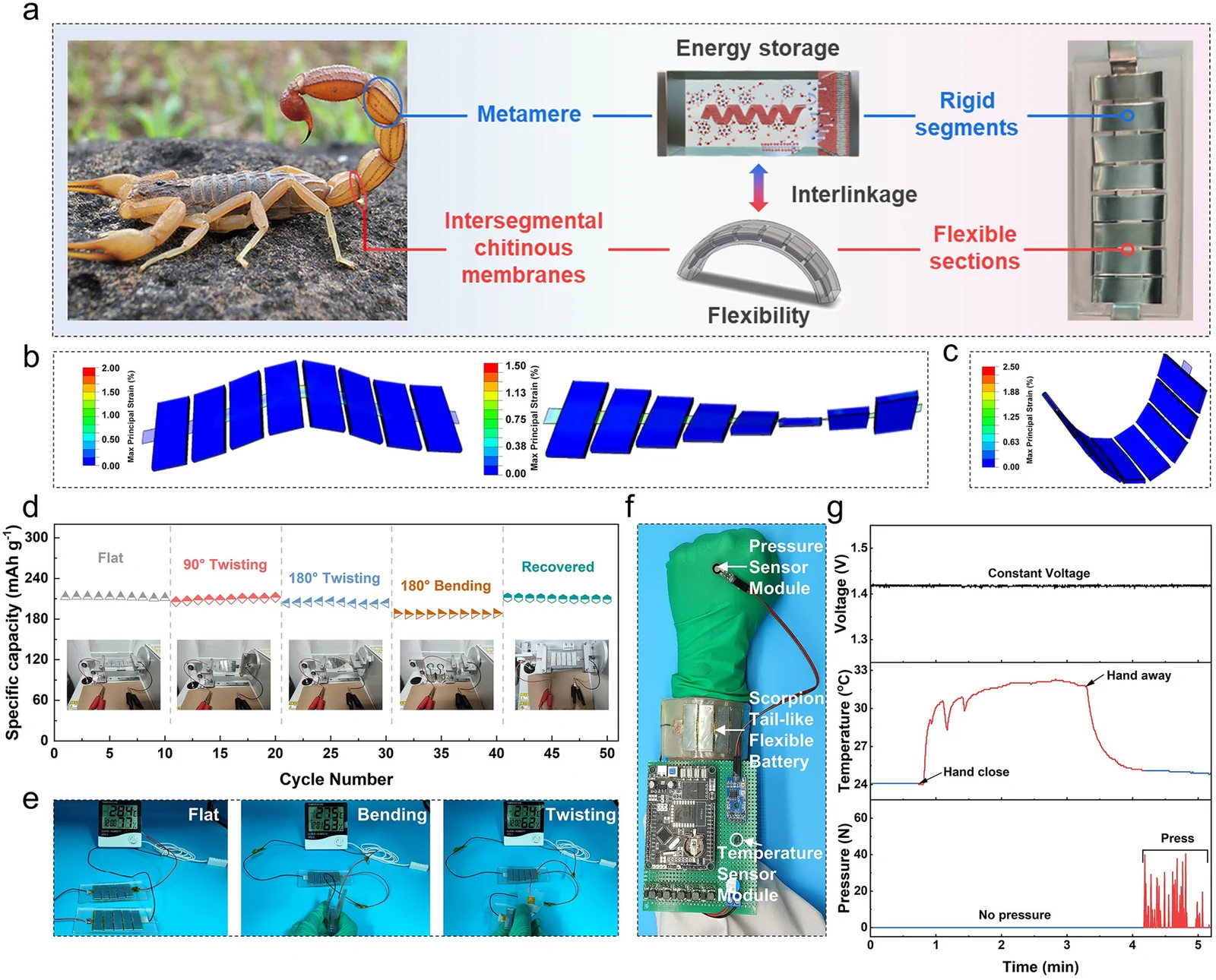
Scorpion tail-mimic flexible cells using the reinforced Zn||MnO_2_ system for wearable electronics. **a** Scorpion tail-inspired bionic design of the flexible cell: rigid segments for energy storage mimic the metameres, the flexible sections emulate the intersegmental chitinous membranes, ensuring flexibility. **b, c** Finite element analysis of the maximal principal strain of the scorpion tail-mimic structure under various deformation conditions. **d** Cycle performance of the bionic Zn||MnO_2_ flexible cells under various mechanical deformations (the insets show the corresponding optical photographs). **e** Photographic pictures show the bionic Zn||MnO_2_ flexible cells driving a thermo-hygrometer under various mechanical deformations. **f** Photographic picture representing the bionic reinforced Zn||MnO_2_ flexible cell worn on the human wrist for powering the multimodal integrated sensors chip for real-time monitoring. **g** Time-dependent voltage profile along with the collected temperature and pressure signals of the multimodal chip shown in **f** powered by the bionic reinforced Zn||MnO_2_ flexible cell

The high safety and capacity endow the bioinspired reinforced aqueous metal-ion batteries the availability to be employed for wearable applications and bio-machine interfaces [67–69]. In this regard, the integration of the mechanical flexibility and durability should be demanded, which is actually challenging. Inspired from the conformation of the scorpion tail, a bionic battery structure was designed [27]. Specifically, the rigid segments resembling metameres functioned as the energy storage units, while the flexible sections emulated the intersegmental chitinous membranes, ensuring high adaptability toward the mechanical deformations (Fig. 6a).

This structural design was assessed to alleviate the formation of surface defects and prevent delamination of the electrode materials from the current collector. The finite element simulations determined that under bending of 15° or twisting of 10°, the unwrapped flexible sections effectively dispersed the stress and bore the dominated principal strains, leaving the rigid segments for energy storage tolerating the minimal stress concentration (Fig. 6b), which preserved the overall structural integrity and cycling stability. Furthermore, when standing a high-curvature bending up to 60° to simulate deformation once worn on a human wrist, the maximum principal stress still primarily distributed within the unwrapped flexible sections, leaving the rigid energy storage segments exhibiting negligible stress accumulation (Fig. 6c). Consequently, the bionic design significantly enhanced the battery’s operational stability under various mechanical deformations, making it appropriate for next-generation wearable applications.

The electrochemical performance evaluations of the bionic flexible cell composed of the reinforced Zn||MnO_2_ system displayed a characteristic CV curve with distinct and reversible redox peaks (Fig. S56), confirming that the scorpion tail-like conformation did not disturb the intrinsic electrochemical reactions. The long-term cycling performance demonstrated that the cell retained 73% of the initial capacity after 100 cycles (Fig. S57), highlighting its convincing sustainability. When subjected to successive deformation states including 90° twisting, 180° twisting, and 180° bending then recovering, the flexible cell delivered capacity retentions of 98%, 96%, 88%, and 99%, respectively (Fig. 6d), manifesting its acceptable flexibility and stable energy output capability. Furthermore, the EIS and AFM characterization (Fig. S58) demonstrated that the Zn||MnO_2_ wearable battery maintained nearly consistent internal resistance and self-assembled bilayer morphologies at the interface after repeated bending, confirming the mechanical robustness. Therefore, the two interconnected flexible cells could power a thermo-hygrometer and a timer regardless of mechanical deformations (Fig. 6e; Videos S7 and S8). Especially, when the flexible cell was worn on the human wrist and connected to an integrated chip equipped with temperature and pressure sensor modules (Fig. 6f), the working voltage remained stable at 1.42 V (Fig. 6g, top panel), allowing reliable operation of the integrated sensors. When a hand approached then retracted from the temperature sensor, the recorded temperature accordingly increased and declined in a real-time manner (Fig. 6g, middle panel). In addition, the pressure sensor could accurately monitor the pressing signals under varying pressures (Fig. 6g, bottom panel). These results exemplified the feasibility of the bionic flexible metal-ion batteries of reinforced performances for powering smart wearable electronics, offering stable energy output under dynamic mechanical conditions.

## Conclusions

The extensive applications in wearable electronics of the aqueous metal-ion batteries are severely hindered due to intrinsic challenges such as electrode/electrolyte interfacial instability during operation and structural damage under bending/stretching. Inspired from nature, a bionic reinforcement strategy was developed, allowing the bioinspired lipopeptide molecules self-assemble into bulk supramolecular nanohelices to accelerate metal-ion transport and organize into a dynamic, ultrathin bilayer at the interface to homogenize ion flux and electric field distributions. This dual-function mechanism synergistically enabled uniform metal-ion plating/stripping, effectively suppressing dendrites or side reactions. As a result, the Zn||Zn symmetric cells demonstrated reinforced cycling stability, while the Zn||Cu asymmetric cells achieved a high CE up to 99.66%, and the Zn||(NH_4_)_2_V_4_O_9_ button cells exhibited remarkable capacity retention. This synergistic reinforcement endowed the Zn||MnO_2_ pouch cell a high initial capacity, allowing to power smartphones and toy electronics in a safe manner. Subsequently, a scorpion tail-mimic flexible battery conformation was developed using the reinforced Zn||MnO_2_ cell, successfully powering the temperature and pressure sensors in integrated wearable electronics regardless of mechanical deformations. This work presents a synergistic reinforcement strategy to effectively regulate metal-ion behaviors in the electrochemical process by bionic self-assembly, offering an infusive advancement in developing high-efficiency aqueous metal-ion batteries as reliable power sources for flexible and wearable applications.

## Supplementary Information

Below is the link to the electronic supplementary material.

Supplementary file1 (DOCX 12410 KB)

Supplementary file2 (MP4 1159 KB)

Supplementary file3 (MP4 1150 KB)

Supplementary file4 (MP4 3816 KB)

Supplementary file5 (MP4 2425 KB)

Supplementary file6 (MP4 2042 KB)

Supplementary file7 (MP4 1564 KB)

Supplementary file8 (MP4 1310 KB)

Supplementary file9 (MP4 2935 KB)

## References

[1] J. Shin, J.W. Song, M.T. Flavin, S. Cho, S. Li et al., A non-contact wearable device for monitoring epidermal molecular flux. Nature **640**(8058), 375–383 (2025). 10.1038/s41586-025-08825-2 40205217 10.1038/s41586-025-08825-2 PMC12182646

[2] S. Niu, N. Matsuhisa, L. Beker, J. Li, S. Wang et al., A wireless body area sensor network based on stretchable passive tags. Nat. Electron. **2**(8), 361–368 (2019). 10.1038/s41928-019-0286-2

[3] W. Gao, S. Emaminejad, H.Y.Y. Nyein, S. Challa, K. Chen et al., Fully integrated wearable sensor arrays for multiplexed *in situ* perspiration analysis. Nature **529**(7587), 509–514 (2016). 10.1038/nature16521 26819044 10.1038/nature16521 PMC4996079

[4] H.U. Chung, B.H. Kim, J.Y. Lee, J. Lee, Z. Xie et al., Binodal, wireless epidermal electronic systems with in-sensor analytics for neonatal intensive care. Science **363**(6430), eaau0780 (2019). 10.1126/science.aau0780 30819934 10.1126/science.aau0780 PMC6510306

[5] C. Dagdeviren, Y. Su, P. Joe, R. Yona, Y. Liu et al., Conformable amplified lead zirconate titanate sensors with enhanced piezoelectric response for cutaneous pressure monitoring. Nat. Commun. **5**, 4496 (2014). 10.1038/ncomms5496 25092496 10.1038/ncomms5496

[6] T.R. Ray, J. Choi, A.J. Bandodkar, S. Krishnan, P. Gutruf et al., Bio-integrated wearable systems: a comprehensive review. Chem. Rev. **119**(8), 5461–5533 (2019). 10.1021/acs.chemrev.8b00573 30689360 10.1021/acs.chemrev.8b00573

[7] T.P. Nguyen, A.D. Easley, N. Kang, S. Khan, S.-M. Lim et al., Polypeptide organic radical batteries. Nature **593**(7857), 61–66 (2021). 10.1038/s41586-021-03399-1 33953410 10.1038/s41586-021-03399-1

[8] J. Yan, Q. Wang, T. Wei, Z. Fan, Recent advances in design and fabrication of electrochemical supercapacitors with high energy densities. Adv. Energy Mater. **4**(4), 1300816 (2014). 10.1002/aenm.201300816

[9] B. Dunn, H. Kamath, J.-M. Tarascon, Electrical energy storage for the grid: a battery of choices. Science **334**(6058), 928–935 (2011). 10.1126/science.1212741 22096188 10.1126/science.1212741

[10] D. Kundu, B.D. Adams, V. Duffort, S.H. Vajargah, L.F. Nazar, A high-capacity and long-life aqueous rechargeable zinc battery using a metal oxide intercalation cathode. Nat. Energy **1**, 16119 (2016). 10.1038/nenergy.2016.119

[11] J. Zheng, Q. Zhao, T. Tang, J. Yin, C.D. Quilty et al., Reversible epitaxial electrodeposition of metals in battery anodes. Science **366**(6465), 645–648 (2019). 10.1126/science.aax6873 31672899 10.1126/science.aax6873

[12] F. Wang, O. Borodin, T. Gao, X. Fan, W. Sun et al., Highly reversible zinc metal anode for aqueous batteries. Nat. Mater. **17**(6), 543–549 (2018). 10.1038/s41563-018-0063-z 29662160 10.1038/s41563-018-0063-z

[13] S. Huang, P. Zhang, J. Lu, J.S. Kim, D.H. Min et al., Molecularly engineered multifunctional imide derivatives for practical Zn metal full cells. Energy Environ. Sci. **17**(20), 7870–7881 (2024). 10.1039/d4ee02867h

[14] H. Lu, X. Zhang, M. Luo, K. Cao, Y. Lu et al., Amino acid-induced interface charge engineering enables highly reversible Zn anode. Adv. Funct. Mater. **31**(45), 2103514 (2021). 10.1002/adfm.202103514

[15] Y. Chen, T. Xue, C. Chen, S. Jang, P.V. Braun et al., Helical peptide structure improves conductivity and stability of solid electrolytes. Nat. Mater. **23**(11), 1539–1546 (2024). 10.1038/s41563-024-01966-1 39107570 10.1038/s41563-024-01966-1

[16] G. Li, Z. Zhao, S. Zhang, L. Sun, M. Li et al., A biocompatible electrolyte enables highly reversible Zn anode for zinc ion battery. Nat. Commun. **14**(1), 6526 (2023). 10.1038/s41467-023-42333-z 37845239 10.1038/s41467-023-42333-z PMC10579325

[17] X. Fan, C. Zhong, J. Liu, J. Ding, Y. Deng et al., Opportunities of flexible and portable electrochemical devices for energy storage: expanding the spotlight onto semi-solid/solid electrolytes. Chem. Rev. **122**(23), 17155–17239 (2022). 10.1021/acs.chemrev.2c00196 36239919 10.1021/acs.chemrev.2c00196

[18] T. Yang, H. Chen, Z. Jia, Z. Deng, L. Chen et al., A damage-tolerant, dual-scale, single-crystalline microlattice in the knobby starfish, *Protoreaster nodosus*. Science **375**(6581), 647–652 (2022). 10.1126/science.abj9472 35143308 10.1126/science.abj9472

[19] N.N. Shi, C.-C. Tsai, F. Camino, G.D. Bernard, N. Yu et al., Thermal physiology. Keeping cool: enhanced optical reflection and radiative heat dissipation in Saharan silver ants. Science **349**(6245), 298–301 (2015). 10.1126/science.aab3564 26089358 10.1126/science.aab3564

[20] Y. Liu, K. He, G. Chen, W.R. Leow, X. Chen, Nature-inspired structural materials for flexible electronic devices. Chem. Rev. **117**(20), 12893–12941 (2017). 10.1021/acs.chemrev.7b00291 28991450 10.1021/acs.chemrev.7b00291

[21] C.C.M. Sproncken, P. Liu, J. Monney, W.S. Fall, C. Pierucci et al., Large-area, self-healing block copolymer membranes for energy conversion. Nature **630**(8018), 866–871 (2024). 10.1038/s41586-024-07481-2 38839964 10.1038/s41586-024-07481-2 PMC11208134

[22] N.H. Joh, T. Wang, M.P. Bhate, R. Acharya, Y. Wu et al., *De novo* design of a transmembrane Zn^2^⁺-transporting four-helix bundle. Science **346**(6216), 1520–1524 (2014). 10.1126/science.1261172 25525248 10.1126/science.1261172 PMC4400864

[23] Y. Chen, T. Yang, Y. Lin, C.M. Evans, Ion transport in helical-helical polypeptide polymerized ionic liquid block copolymers. Nat. Commun. **16**(1), 2451 (2025). 10.1038/s41467-025-57784-9 40069217 10.1038/s41467-025-57784-9 PMC11897142

[24] S. Chen, Y. Xia, R. Zeng, Z. Luo, X. Wu et al., Ordered planar plating/stripping enables deep cycling zinc metal batteries. Sci. Adv. **10**(10), eadn2265 (2024). 10.1126/sciadv.adn2265 38446894 10.1126/sciadv.adn2265 PMC10917354

[25] C. Vicente-Garcia, I. Colomer, Lipopeptides as tools in catalysis, supramolecular, materials and medicinal chemistry. Nat. Rev. Chem. **7**(10), 710–731 (2023). 10.1038/s41570-023-00532-8 37726383 10.1038/s41570-023-00532-8

[26] I.W. Hamley, A. Adak, V. Castelletto, Influence of chirality and sequence in lysine-rich lipopeptide biosurfactants and micellar model colloid systems. Nat. Commun. **15**(1), 6785 (2024). 10.1038/s41467-024-51234-8 39117639 10.1038/s41467-024-51234-8 PMC11310517

[27] S.K. Mitchell, X. Wang, E. Acome, T. Martin, K. Ly et al., An easy-to-implement toolkit to create versatile and high-performance HASEL actuators for untethered soft robots. Adv. Sci. **6**(14), 1900178 (2019). 10.1002/advs.201900178 10.1002/advs.201900178 PMC6662077 31380206

[28] D. Jia, K. Tao, J. Wang, C. Wang, X. Zhao et al., Dynamic adsorption and structure of interfacial bilayers adsorbed from lipopeptide surfactants at the hydrophilic silicon/water interface: effect of the headgroup length. Langmuir **27**(14), 8798–8809 (2011). 10.1021/la105129m 21675796 10.1021/la105129m

[29] D. Jia, K. Tao, J. Wang, C. Wang, X. Zhao et al., Interfacial adsorption of lipopeptide surfactants at the silica/water interface studied by neutron reflection. Soft Matter **7**(5), 1777–1788 (2011). 10.1039/C0SM00581A

[30] G. Kresse, J. Furthmüller, Efficiency of ab-initio total energy calculations for metals and semiconductors using a plane-wave basis set. Comput. Mater. Sci. **6**(1), 15–50 (1996). 10.1016/0927-0256(96)00008-0 10.1103/physrevb.54.11169 9984901

[31] J.P. Perdew, K. Burke, M. Ernzerhof, Generalized gradient approximation made simple. Phys. Rev. Lett. **77**(18), 3865–3868 (1996). 10.1103/physrevlett.77.3865 10062328 10.1103/PhysRevLett.77.3865

[32] S. Grimme, J. Antony, S. Ehrlich, H. Krieg, A consistent and accurate *ab initio* parametrization of density functional dispersion correction (DFT-D) for the 94 elements H-Pu. J. Chem. Phys. **132**(15), 154104 (2010). 10.1063/1.3382344 20423165 10.1063/1.3382344

[33] A.V. Marenich, C.J. Cramer, D.G. Truhlar, Universal solvation model based on solute electron density and on a continuum model of the solvent defined by the bulk dielectric constant and atomic surface tensions. J. Phys. Chem. B **113**(18), 6378–6396 (2009). 10.1021/jp810292n 19366259 10.1021/jp810292n

[34] W.R.P. Scott, P.H. Hünenberger, I.G. Tironi, A.E. Mark, S.R. Billeter et al., The GROMOS biomolecular simulation program package. J. Phys. Chem. A **103**(19), 3596–3607 (1999). 10.1021/jp984217f

[35] U. Essmann, L. Perera, M.L. Berkowitz, T. Darden, H. Lee et al., A smooth particle mesh Ewald method. J. Chem. Phys. **103**(19), 8577–8593 (1995). 10.1063/1.470117

[36] K. Ma, S. Chen, R. Zeng, Z. Luo, Y. Wang et al., Self-assembled supramolecular pillared arrays as bionic interface to stabilize zinc metal anodes. Chem. Eng. J. **503**, 158660 (2025). 10.1016/j.cej.2024.158660

[37] M.K. Banjare, R. Kurrey, T. Yadav, S. Sinha, M.L. Satnami et al., A comparative study on the effect of imidazolium-based ionic liquid on self-aggregation of cationic, anionic and nonionic surfactants studied by surface tension, conductivity, fluorescence and FTIR spectroscopy. J. Mol. Liq. **241**, 622–632 (2017). 10.1016/j.molliq.2017.06.009

[38] T. Zemb, M. Dubois, B. Deme, T. Gulik-Krzywicki, Self-assembly of flat nanodiscs in salt-free catanionic surfactant solutions. Science **283**(5403), 816–819 (1999). 10.1126/science.283.5403.816 9933158 10.1126/science.283.5403.816

[39] Y. Yang, H. Sai, S.A. Egner, R. Qiu, L.C. Palmer et al., Peptide programming of supramolecular vinylidene fluoride ferroelectric phases. Nature **634**(8035), 833–841 (2024). 10.1038/s41586-024-08041-4 39385033 10.1038/s41586-024-08041-4

[40] L. Ziserman, H.-Y. Lee, S.R. Raghavan, A. Mor, D. Danino, Unraveling the mechanism of nanotube formation by chiral self-assembly of amphiphiles. J. Am. Chem. Soc. **133**(8), 2511–2517 (2011). 10.1021/ja107069f 21244023 10.1021/ja107069f

[41] X. Shi, J. Xie, J. Wang, S. Xie, Z. Yang et al., A weakly solvating electrolyte towards practical rechargeable aqueous zinc-ion batteries. Nat. Commun. **15**, 302 (2024). 10.1038/s41467-023-44615-y 38182604 10.1038/s41467-023-44615-y PMC10770389

[42] L. Bin, S. He, W. Feng, H. Wu, M. Kang et al., Lipid analogues enhance the lifespans of reversible Zn-based aqueous batteries *via* optimal interfacial assembly. Adv. Funct. Mater. **35**(35), 2502041 (2025). 10.1002/adfm.202502041

[43] X. Liu, J.-W. Qian, J.-W. Chen, Y.-K. Xu, W.-Y. Wang et al., A sustainable and scalable approach for *in situ* induction of gradient nucleation sites in biomass-derived interface layers for ultra-stable aqueous zinc metal batteries. Angew. Chem. Int. Ed. **64**(26), e202504613 (2025). 10.1002/anie.202504613 10.1002/anie.202504613 40130742

[44] X. Yu, Z. Li, X. Wu, H. Zhang, Q. Zhao et al., Ten concerns of Zn metal anode for rechargeable aqueous zinc batteries. Joule **7**(6), 1145–1175 (2023). 10.1016/j.joule.2023.05.004

[45] D. Lin, Y. Liu, Y. Cui, Reviving the lithium metal anode for high-energy batteries. Nat. Nanotechnol. **12**(3), 194–206 (2017). 10.1038/nnano.2017.16 28265117 10.1038/nnano.2017.16

[46] Y. Yang, C. Liu, Z. Lv, H. Yang, Y. Zhang et al., Synergistic manipulation of Zn^2+^ ion flux and desolvation effect enabled by anodic growth of a 3D ZnF_2_ matrix for long-lifespan and dendrite-free Zn metal anodes. Adv. Mater. **33**(11), 2007388 (2021). 10.1002/adma.202007388 10.1002/adma.202007388 33554430

[47] X. Li, Z. Li, C. Li, F. Tian, Z. Qiao et al., Facilitating uniform lithium-ion transport *via* polymer-assisted formation of unique interfaces to achieve a stable 4.7 V Li metal battery. Natl. Sci. Rev. **12**(6), nwaf182 (2025). 10.1093/nsr/nwaf182 40475066 10.1093/nsr/nwaf182 PMC12139002

[48] M. Xia, J. Zhou, B. Lu, Comprehensive insights into aqueous potassium-ion batteries. Adv. Energy Mater. **15**(12), 2404032 (2025). 10.1002/aenm.202404032

[49] D. Dong, T. Wang, Y. Sun, J. Fan, Y.-C. Lu, Hydrotropic solubilization of zinc acetates for sustainable aqueous battery electrolytes. Nat. Sustain. **6**(11), 1474–1484 (2023). 10.1038/s41893-023-01172-y

[50] Z. Luo, Y. Xia, S. Chen, X. Wu, E. Akinlabi et al., A homogeneous plating/stripping mode with fine grains for highly reversible Zn anodes. Energy Environ. Sci. **17**(18), 6787–6798 (2024). 10.1039/D4EE02264E

[51] W. Zhang, M. Dong, K. Jiang, D. Yang, X. Tan et al., Self-repairing interphase reconstructed in each cycle for highly reversible aqueous zinc batteries. Nat. Commun. **13**(1), 5348 (2022). 10.1038/s41467-022-32955-0 36097022 10.1038/s41467-022-32955-0 PMC9468148

[52] R. Chen, C. Zhang, J. Li, Z. Du, F. Guo et al., A hydrated deep eutectic electrolyte with finely-tuned solvation chemistry for high-performance zinc-ion batteries. Energy Environ. Sci. **16**(6), 2540–2549 (2023). 10.1039/D3EE00462G

[53] X. Wang, W. Zhou, L. Wang, Y. Zhang, S. Li et al., Benchmarking corrosion with anionic polarity index for stable and fast aqueous batteries even in low-concentration electrolyte. Adv. Mater. **37**(14), e2501049 (2025). 10.1002/adma.202501049 40025972 10.1002/adma.202501049

[54] Y. Lv, C. Huang, M. Zhao, M. Fang, Q. Dong et al., Synergistic anion-cation chemistry enables highly stable Zn metal anodes. J. Am. Chem. Soc. **147**(10), 8523–8533 (2025). 10.1021/jacs.4c16932 40033817 10.1021/jacs.4c16932

[55] K. Wang, H. Zhan, W. Su, X.-X. Liu, X. Sun, Ordered interface regulation at Zn electrodes induced by trace gum additives for high-performance aqueous batteries. Energy Environ. Sci. **18**(3), 1398–1407 (2025). 10.1039/D4EE04100C

[56] J. Luo, L. Xu, Y. Zhou, T. Yan, Y. Shao et al., Regulating the inner Helmholtz plane with a high donor additive for efficient anode reversibility in aqueous Zn-ion batteries. Angew. Chem. Int. Ed. **62**(21), e202302302 (2023). 10.1002/anie.202302302 10.1002/anie.202302302 36959698

[57] S.-J. Zhang, J. Hao, H. Wu, Q. Chen, C. Ye et al., Protein interfacial gelation toward shuttle-free and dendrite-free Zn-iodine batteries. Adv. Mater. **36**(35), e2404011 (2024). 10.1002/adma.202404011 38970531 10.1002/adma.202404011

[58] L. Zheng, H. Li, X. Wang, Z. Chen, C. Hu et al., Competitive solvation-induced interphases enable highly reversible Zn anodes. ACS Energy Lett. **8**(5), 2086–2096 (2023). 10.1021/acsenergylett.3c00650

[59] S. Chen, Y. Ying, L. Ma, D. Zhu, H. Huang et al., An asymmetric electrolyte to simultaneously meet contradictory requirements of anode and cathode. Nat. Commun. **14**, 2925 (2023). 10.1038/s41467-023-38492-8 37217467 10.1038/s41467-023-38492-8 PMC10202929

[60] Y. Wang, Q. Li, H. Hong, S. Yang, R. Zhang et al., Lean-water hydrogel electrolyte for zinc ion batteries. Nat. Commun. **14**, 3890 (2023). 10.1038/s41467-023-39634-8 37393327 10.1038/s41467-023-39634-8 PMC10314915

[61] X. Hu, H. Dong, N. Gao, T. Wang, H. He et al., Self-assembled polyelectrolytes with ion-separation accelerating channels for highly stable Zn-ion batteries. Nat. Commun. **16**(1), 2316 (2025). 10.1038/s41467-025-57666-0 40057473 10.1038/s41467-025-57666-0 PMC11890744

[62] F. Zhao, J. Feng, H. Dong, R. Chen, T. Munshi et al., Ultrathin protection layer *via* rapid sputtering strategy for stable aqueous zinc ion batteries. Adv. Funct. Mater. **34**(51), 2409400 (2024). 10.1002/adfm.202409400

[63] D. Wang, S. Hu, T. Li, C. Chang, S. Li et al., Anti-dendrite separator interlayer enabling staged zinc deposition for enhanced cycling stability of aqueous zinc batteries. Nat. Commun. **16**(1), 259 (2025). 10.1038/s41467-024-55153-6 39747007 10.1038/s41467-024-55153-6 PMC11696076

[64] T. Li, H. Yang, X. Dong, H. Ma, J. Cai et al., Co-regulation of interface and bulk for enhanced localized high-concentration electrolytes in stable and practical zinc metal batteries. Angew. Chem. Int. Ed. **64**(29), e202501183 (2025). 10.1002/anie.202501183 10.1002/anie.202501183 40384603

[65] W. Lyu, X. Yu, Y. Lv, A.M. Rao, J. Zhou et al., Building stable solid-state potassium metal batteries. Adv. Mater. **36**(24), 2305795 (2024). 10.1002/adma.202305795 10.1002/adma.202305795 38294305

[66] Y. Zhang, H. Jiang, L. Xu, Z. Gao, C. Meng, Ammonium vanadium oxide [(NH_4_)_2_V_4_O_9_] sheets for high capacity electrodes in aqueous zinc ion batteries. ACS Appl. Energy Mater. **2**(11), 7861–7869 (2019). 10.1021/acsaem.9b01299

[67] Z. Chen, P.-C. Hsu, J. Lopez, Y. Li, J.W.F. To et al., Fast and reversible thermoresponsive polymer switching materials for safer batteries. Nat. Energy **1**, 15009 (2016). 10.1038/nenergy.2015.9

[68] J. Kim, J. Jeong, S.H. Ko, Electrochemical biosensors for point-of-care testing. Bio-des. Manuf. **7**(4), 548–565 (2024). 10.1007/s42242-024-00301-6

[69] W. Guo, F. Tian, D. Fu, H. Cui, H. Song et al., High-performance aqueous calcium ion batteries enabled by Zn metal anodes with stable ion-conducting interphases. Nano Lett. **24**(39), 12095–12101 (2024). 10.1021/acs.nanolett.4c02778 39291849 10.1021/acs.nanolett.4c02778

