# Supplementary material for: Bioinspired Self-Assembly-Reinforced Ion Transport and Interface Regulation Enables Sustainable Metal-Ion Batteries for Wearable Electronics
Source: Nanomicro Lett. 2026 Jan 26;18:222. doi: 10.1007/s40820-026-02071-5 (PMC12835463; doi:10.1007/s40820-026-02071-5)
Supplement: Supplementary file 1 — Supplementary file1 (DOCX 12410 KB) [file 40820_2026_2071_MOESM1_ESM.docx]

Supporting Information for

**Bioinspired Self-Assembly Reinforced Ion Transport and Interface Regulation Enables Sustainable Metal-Ion Batteries for Wearable Electronics**

Kang Ma^1, 2, 3, #^, Ran Zeng^1, 2, 3, #^, Shuang Chen^2, 4^, Yu Zhang^2, 3^, Jiqian Wang^3, 5^, Xuzhi Hu^6^, Yinzhu Jiang^2, 4^, Hai Xu^3, 5^, Hongge Pan^4, 7^, Deqing Mei^1^, Ehud Gazit^2, 8, 9,^ *, Kai Tao^1, 2, 3,^ *

^1^ State Key Laboratory of Fluid Power and Mechatronic Systems, Zhejiang Key Laboratory of Advanced Equipment Manufacturing and Measurement Technology, School of Mechanical Engineering, Zhejiang University, Hangzhou 310058, P. R. China

^2^ Zhejiang-Israel Joint Laboratory of Self-Assembling Functional Materials, ZJU-Hangzhou Global Scientific and Technological Innovation Center, Hangzhou 311215, P. R. China

^3^ Zhejiang-Ireland Joint Laboratory of Bio-Organic Dielectrics & Devices, Zhejiang University, Hangzhou 310058, P. R. China

^4^ State Key Laboratory of Silicon and Advanced Semiconductor Materials, School of Materials Science and Engineering, Zhejiang University, Hangzhou 310058, P. R. China

^5^ Department of Biological and Energy Chemical Engineering, China University of Petroleum (East China), 66 Changjiang West Road, Qingdao 266580, P. R. China

^6^ State Key Laboratory of Solid Lubrication, Lanzhou Institute of Chemical Physics, Chinese Academy of Sciences, No.18, Tianshui Middle Road, Lanzhou 730000, P. R. China

^7^ Institute of Science and Technology for New Energy, Xi'an Technological University, Xi'an 710021, P. R. China

^8^ The Shmunis School of Biomedicine and Cancer Research, George S. Wise Faculty of Life Sciences, Tel Aviv University, Tel Aviv 6997801, Israel

^9^ Department of Materials Science and Engineering, The Iby and Aladar Fleischman Faculty of Engineering, Tel Aviv University, Tel Aviv 6997801, Israel

^#^Kang Ma and Ran Zeng contributed equally to this work.

*Corresponding authors. E-mail: [ehudg@post.tau.ac.il](mailto:ehudg@post.tau.ac.il) (Ehud Gazit); [kai.tao@zju.edu.cn](mailto:kai.tao@zju.edu.cn) (Kai Tao)

**Supplementary Figures and Tables**


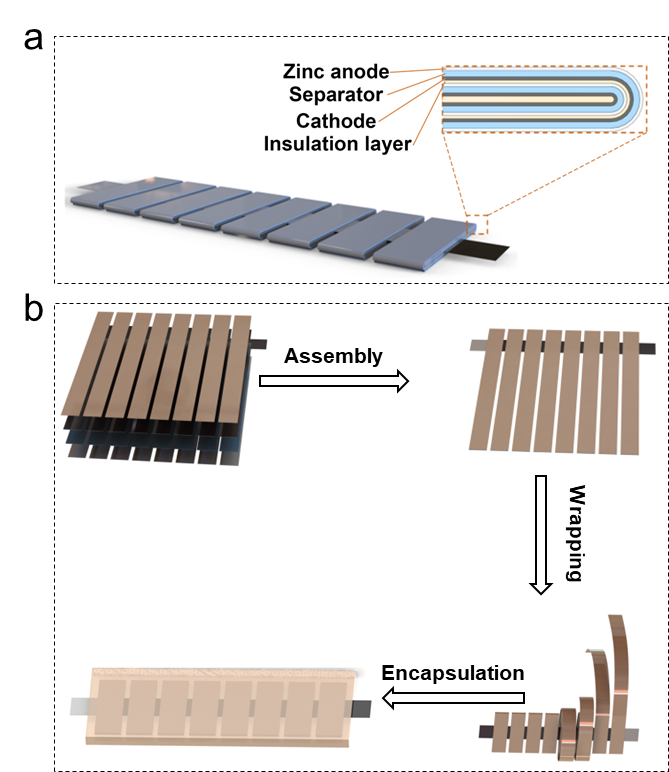


**Fig. S1** Schematic cartoon showing the fabrication process of the scorpion tail-mimic flexible battery. **a,** Structural scheme and internal configuration of the scorpion tail-mimic Zn||MnO_2_ flexible cell. **b,** Fabrication process: the multilayered electrodes are cut into the designed shape, the extending strips are then wound around the central trunk to form the scorpion tail-like structures


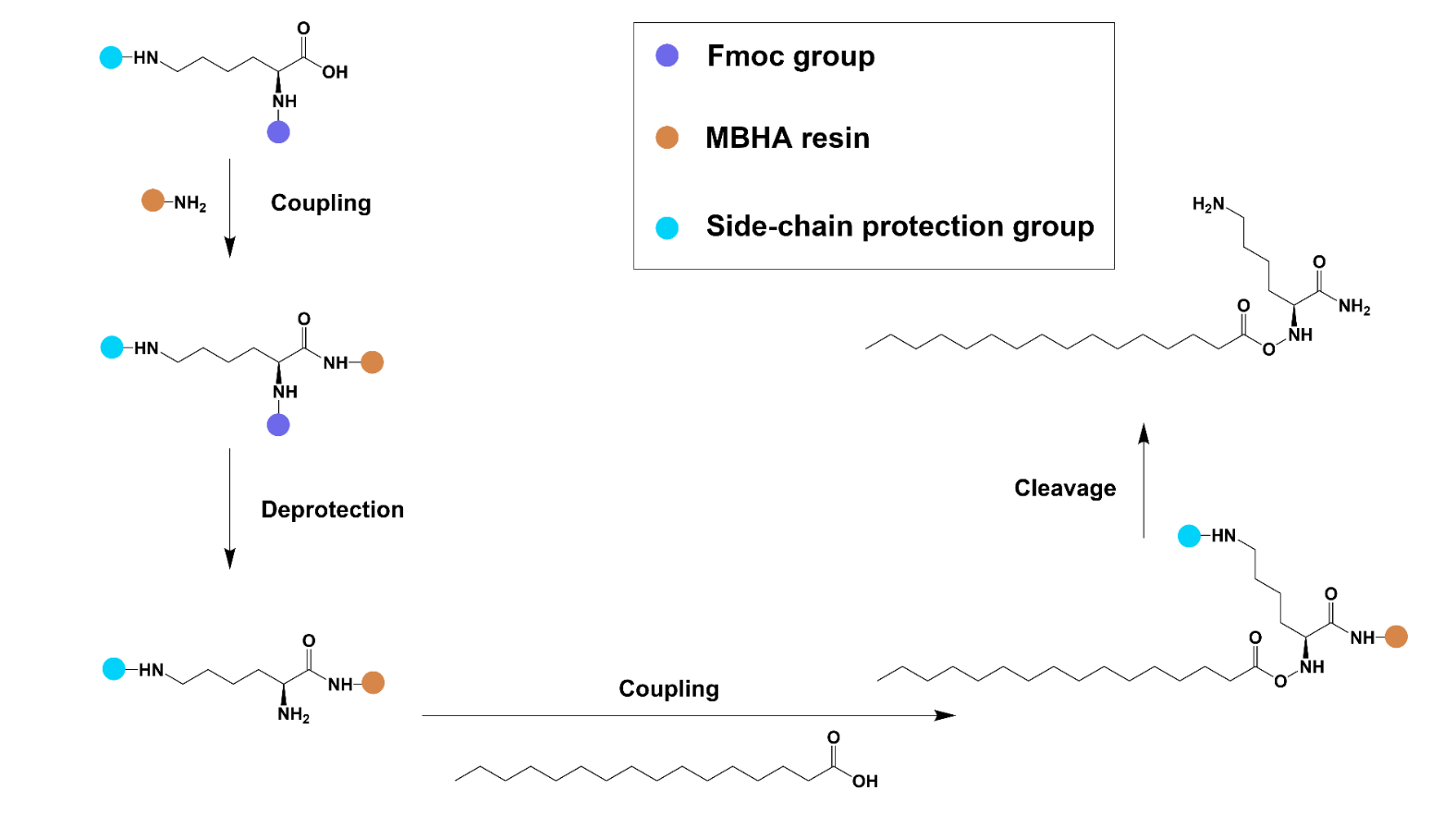


**Fig. S2** Flow chart representing the synthesizing process of the designed C_16_K lipopeptide through the Fmoc solid-phase peptide synthesis strategy


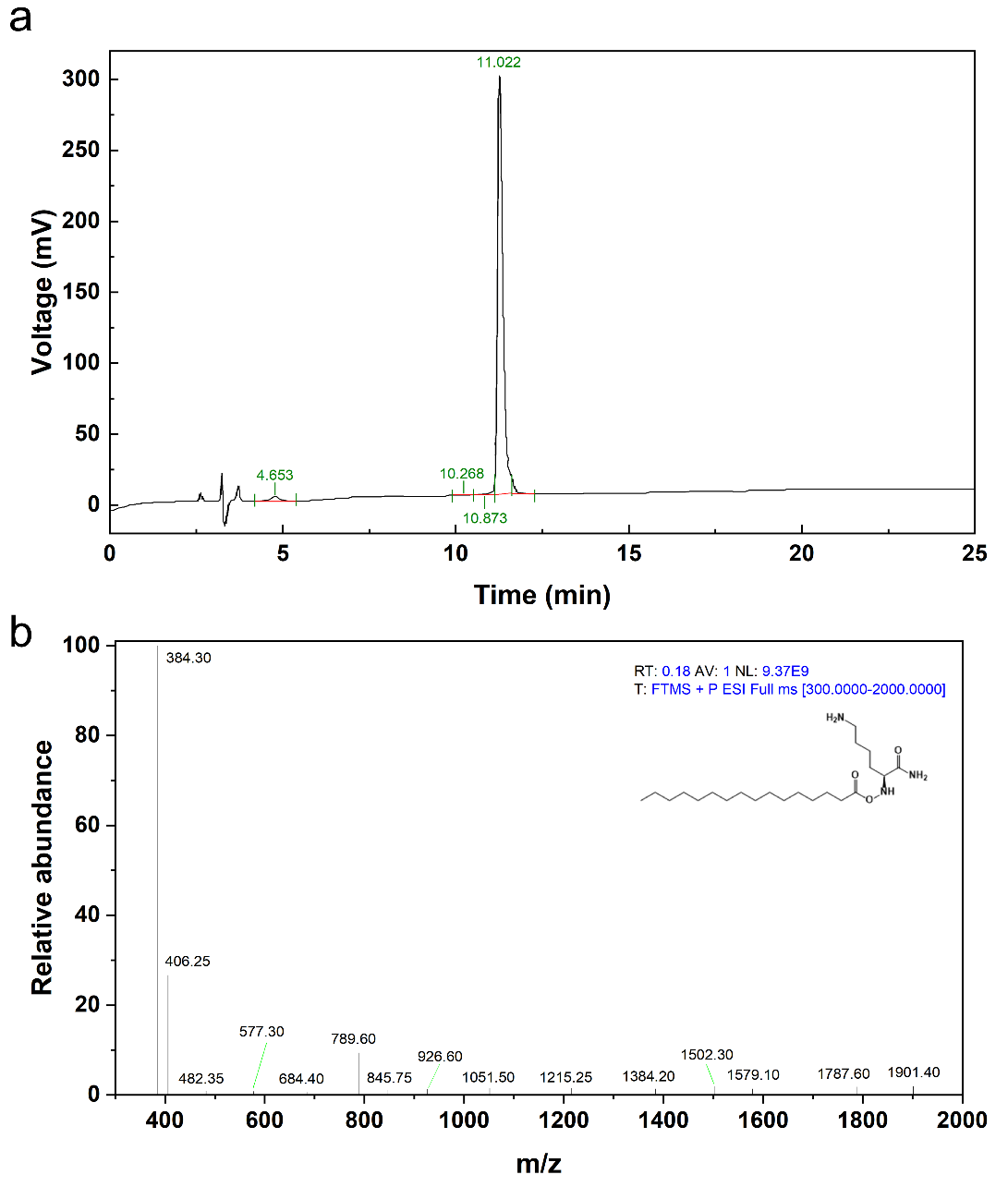


**Fig. S3** RP-HPLC and MS spectra of the synthesized C_16_K lipopeptide. **a,** RP-HPLC profile of the synthesized C_16_K. The purity was analyzed to be more than 97.8%. A gradient elution mode was employed. The monitoring wavelength was set at 220 nm, and the flow rate was 1.0 mL min^-1^. **b,** MS pattern of the lipopeptide, showing its relative molecular weight of 383.30 [384.30 = *MW* + H]


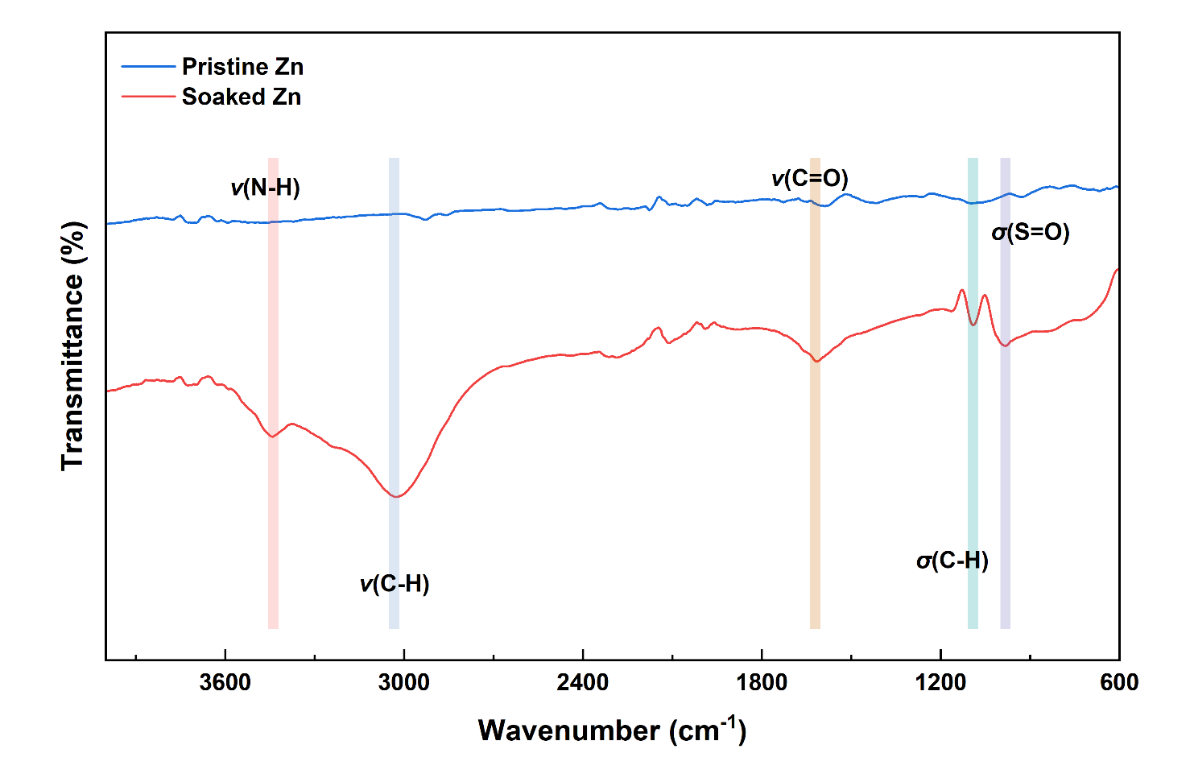


**Fig. S4** FTIR spectra of the pristine Zn foil and Zn soaked in the ZnSO_4_ electrolyte at the presence of C_16_K (ZnSO_4_: 2.0 M, C_16_K: 0.10 mM).

The peaks at 3440 cm^-1^, 3031 cm^-1^, 1617 cm^-1^ and 1092 cm^-1^ were assigned to the stretching vibrations of *v*_(N-H)_, *v*_(C-H)_, *v*_(C=O)_ and the bending vibration of σ_(C-H)_, respectively, indicating the adsorption of C_16_K molecules onto the Zn foil.

**
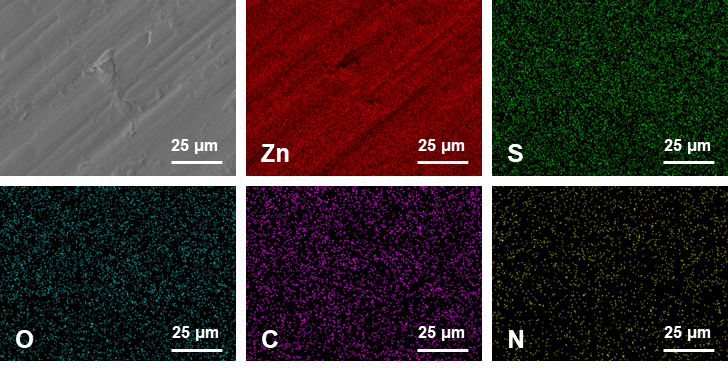
**

**Fig. S5** SEM image and corresponding EDS mappings of the Zn foil soaked in the ZnSO_4_ electrolyte at the presence of C_16_K (ZnSO_4_: 2.0 M, C_16_K: 0.10 mM)

**Table S1** The weight percentage and corresponding standard deviation of different elements from Fig. S5

| **Element** | **Signal type** | **(wt.)%** | **(wt.)% sigma** |
| --- | --- | --- | --- |
| C | EDS | 11.91 | 0.36 |
| N | EDS | 1.14 | 0.24 |
| O | EDS | 1.97 | 0.09 |
| S | EDS | 1.13 | 0.03 |
| Zn | EDS | 83.85 | 0.41 |
| Total |  | 100 |  |

Combining Fig. S5 and Table S1, it was revealed a uniform distribution of C_16_K molecules across the Zn foil.


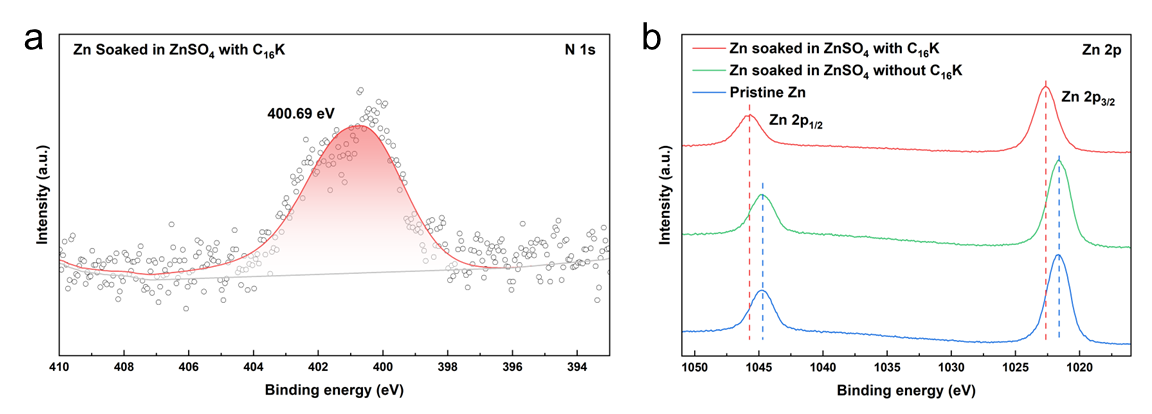


**Fig. S6** High-resolution XPS spectra of Zn foils: **a** N1s spectra of the Zn foil soaked in the ZnSO_4_ electrolyte at the presence of C_16_K (ZnSO_4_: 2.0 M, C_16_K: 0.10 mM). **b** Zn 2p spectra of pristine Zn and Zn foils soaked in the ZnSO_4_ electrolyte in the absence or at the presence of C_16_K for 30 min (ZnSO_4_: 2.0 M, C_16_K: 0.10 mM)

The XPS profiles showed a noticeable positive shift of Zn 2p peaks to higher binding energies after immersion in the ZnSO_4_ electrolyte at the presence of C_16_K, signaling the formation of chemical bonds between C_16_K and the Zn surficial atoms.


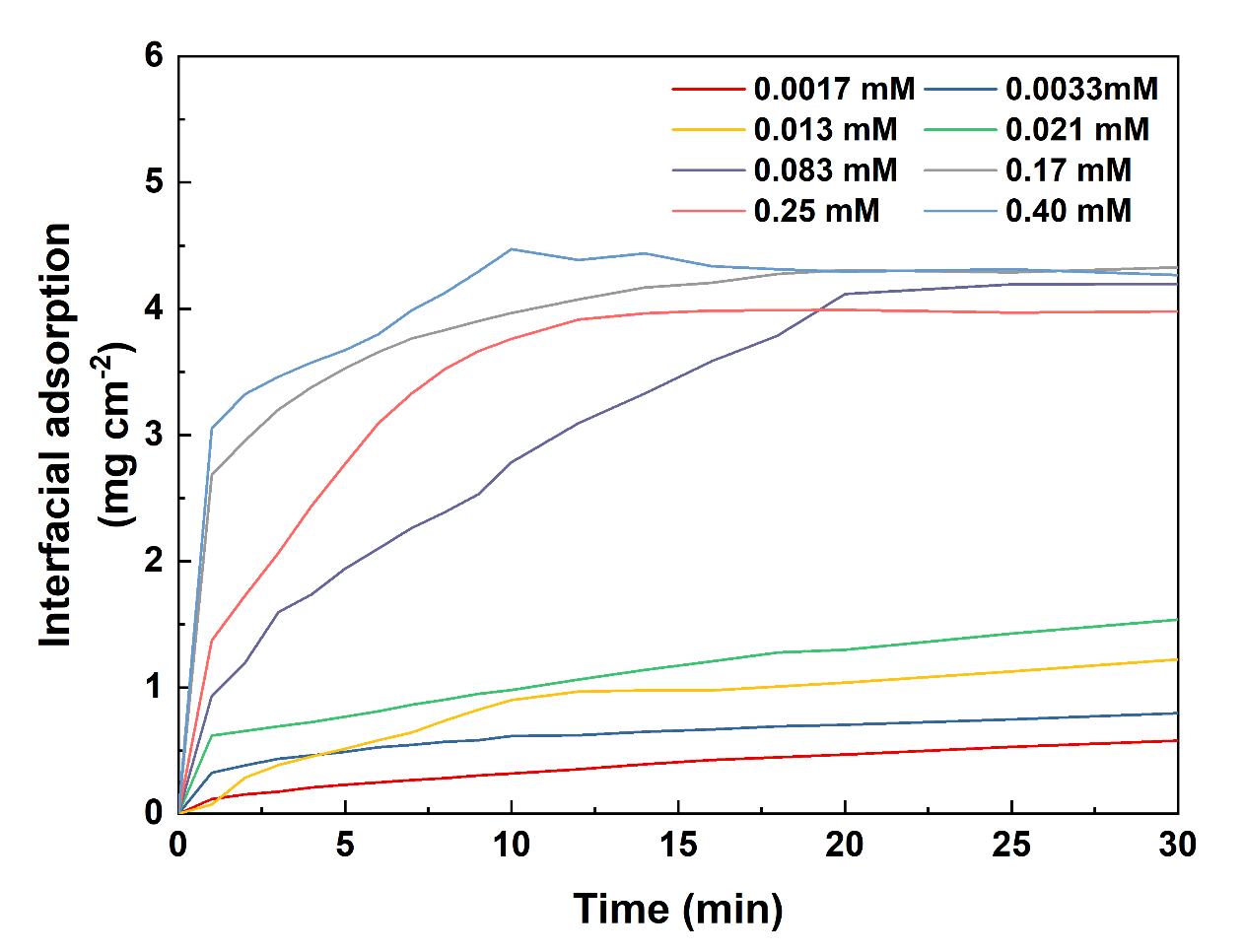


**Fig. S7** Concentration-dependent dynamic adsorption curves of C_16_K on the liquid-solid interface

**
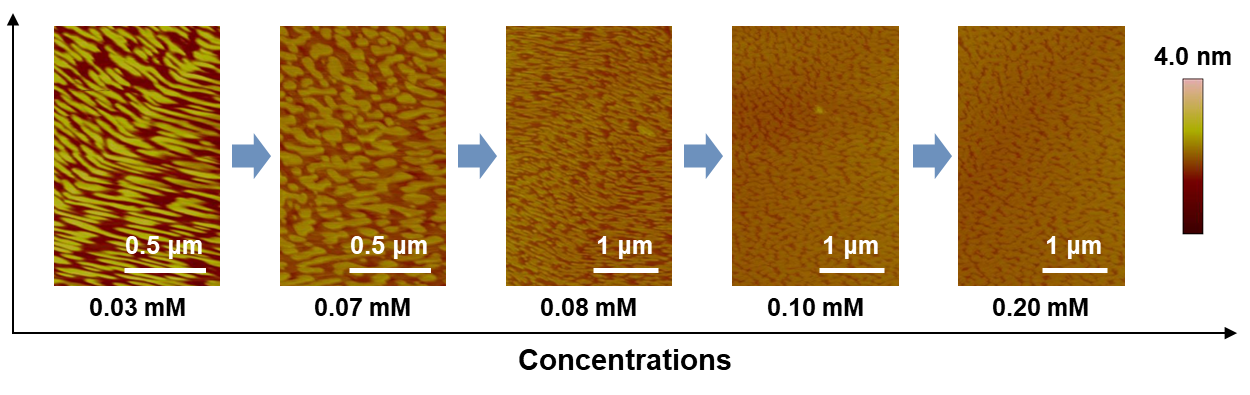
**

**Fig. S8** AFM images showing concentration-dependent topological evolution of the interfacial self-assembly of C_16_K. From left to right: 0.03 mM, 0.07 mM, 0.08 mM, 0.10 mM, 0.20 mM


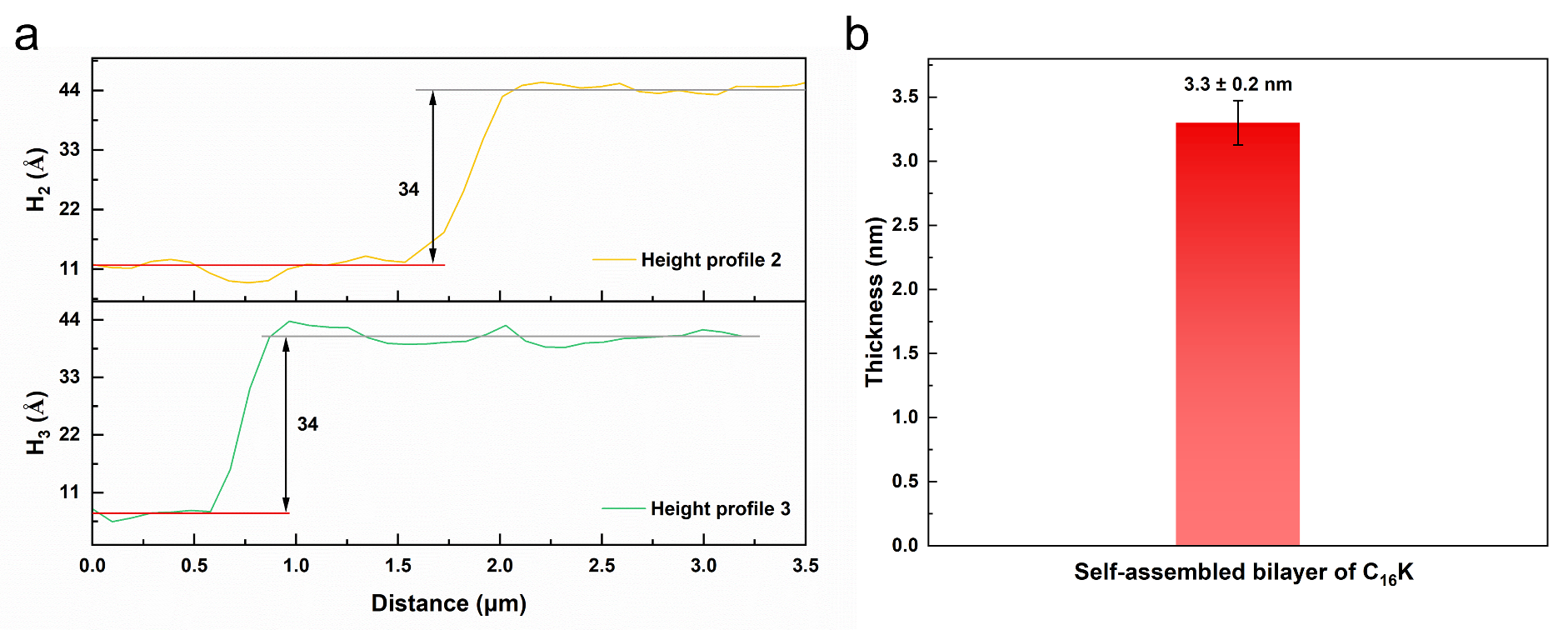


**Fig. S9** Height profiles along Line #2 and #3 in Fig. 1d in the main text. Combined with the value from Line#1, the statistical average height of the membrane was 3.3 ± 0.2 nm from AFM results

**Table S2** Summary of the structural parameters extracted from fitting of the NR data in Fig. 1e in the main text

|  | **Substrate** | **Bottom Lysine (K)** | **Palmitic acid alkyl chain (C_16_)** | **Top Lysine (K)** |
| --- | --- | --- | --- | --- |
| *t* (Å) | 20 | 6 | 20 | 6 |
| *VF* (%) | - | 35 | 35 | 35 |
| *M*  (mg m^-2^) | - | 2.84 | 9.45 | 2.84 |

Tip: *t*, *VF* and *M* are the scattering thickness, volume fraction and adsorbed mass, respectively.


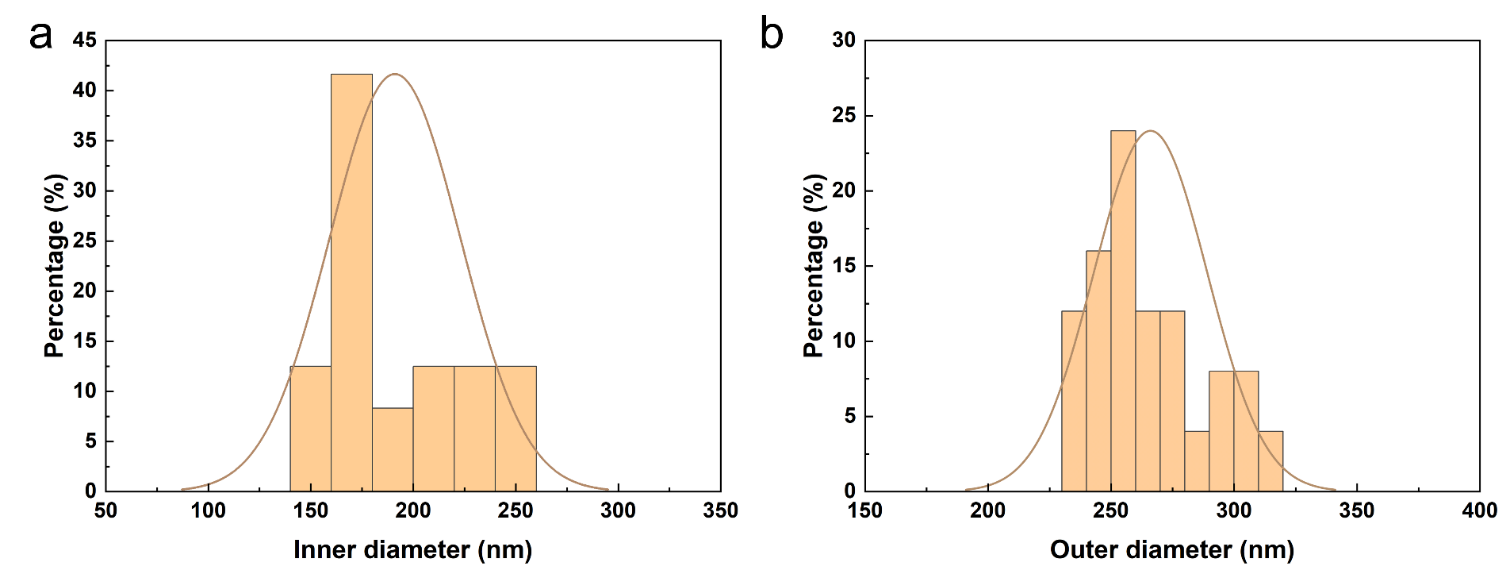


**Fig. S10** Statistical diameter distributions of the C_16_K self-assembled nanotubes. **a,** inner diameter, **b,** outer diameter

**~~
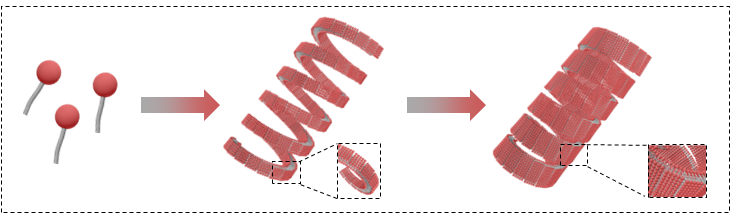
~~**

**Fig. S11** Schematic cartoon illustrating the morphological evolution of C_16_K bulk self-assembly


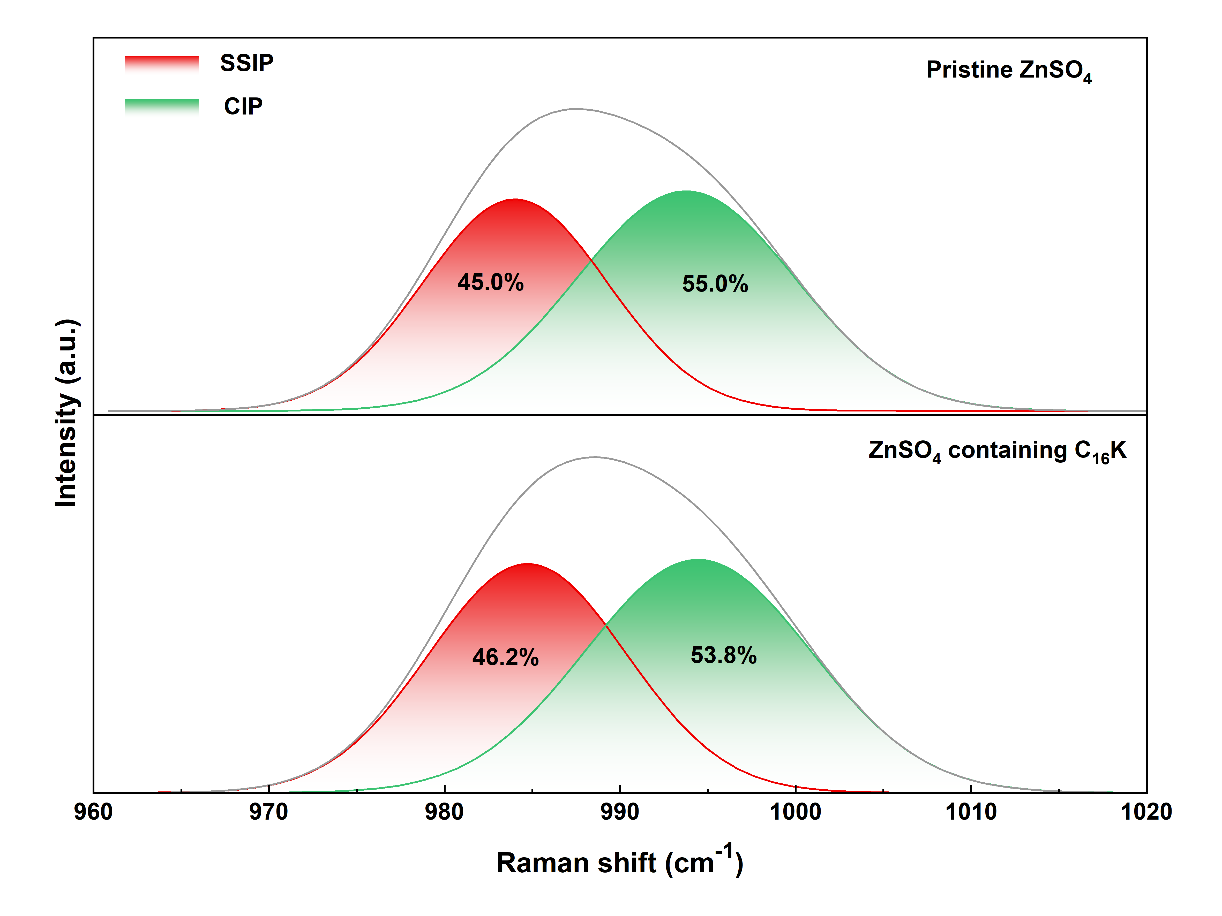


**Fig. S12** Raman spectra of the ZnSO_4_ electrolyte (top panel) in the absence or (lower panel) at the presence of C_16_K (ZnSO_4_: 2.0 M, C_16_K: 0.10 mM)

**
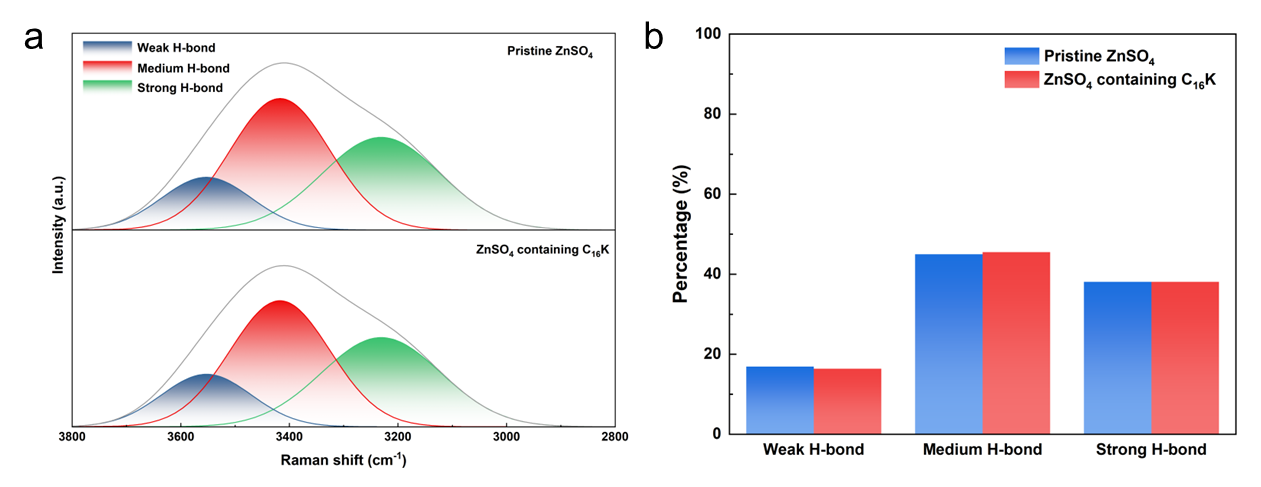
**

**Fig. S13** Comparison of the hydrogen bonds in the ZnSO_4_ electrolyte in the absence or at the presence of C_16_K (ZnSO_4_: 2.0 M, C_16_K: 0.10 mM). **a,** Raman spectra and corresponding fitting curves in the range of 3800 cm^-1^ - 2800 cm^-1^. **b,** Ratios of different hydrogen bonds calculated from the fitting results in (a)


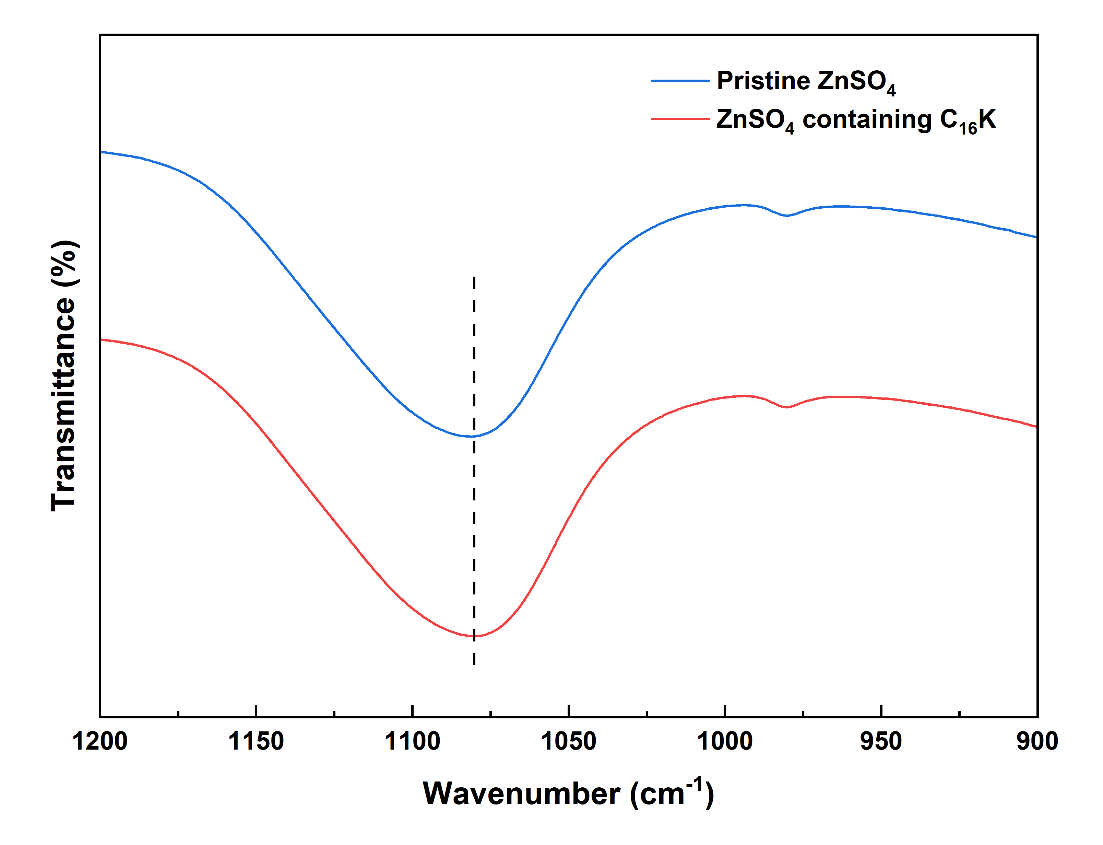


**Fig. S14** FTIR spectra of the ZnSO_4_ electrolyte in the absence or at the presence of C_16_K (ZnSO_4_: 2.0 M, C_16_K: 0.10 mM)


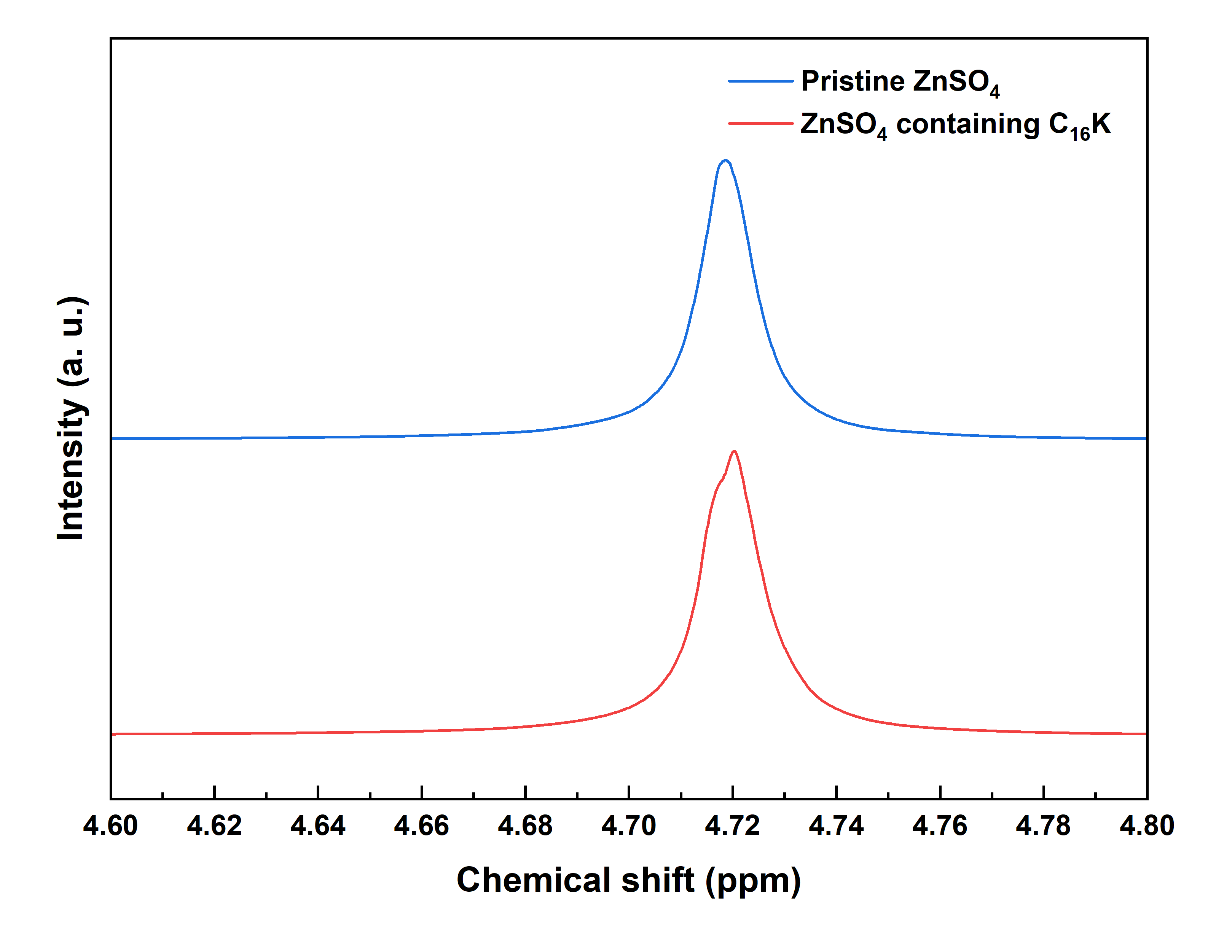


**Fig. S15** ^1^H-NMR spectra of the ZnSO_4_ electrolyte in the absence or at the presence of C_16_K (ZnSO_4_: 2.0 M, C_16_K: 0.10 mM, D_2_O as the solvent)


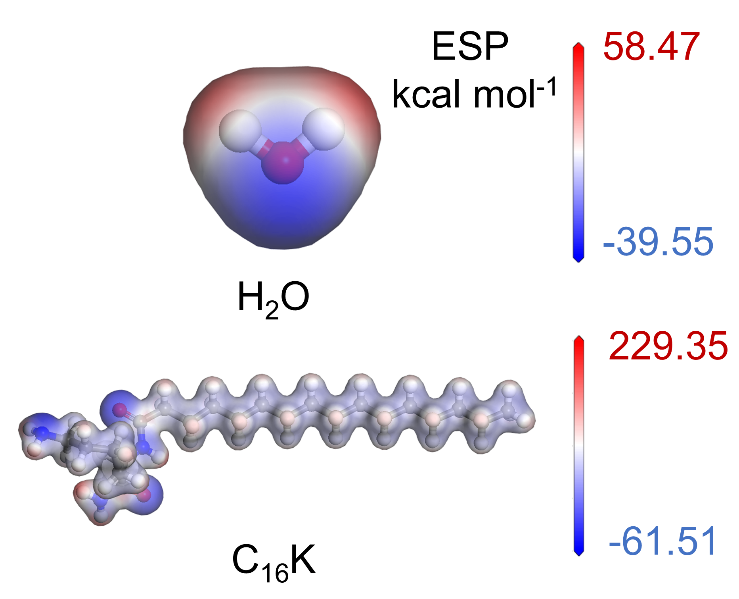


**Fig. S16** ESP distribution of (top)a water molecule and (bottom) a C_16_K molecule

**
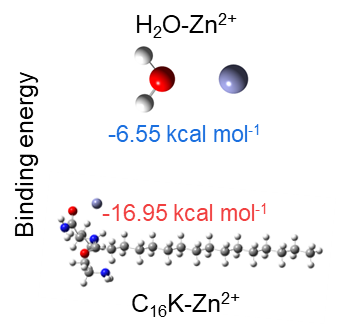
**

**Fig. S17** Calculated binding energies of H_2_O-Zn^2+^ and C_16_K-Zn^2+^


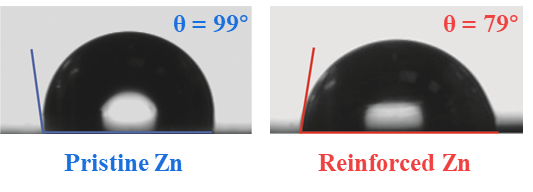


**Fig. S18** Contact angle measurements of the (left) pristine and (right) C_16_K-modified Zn foil


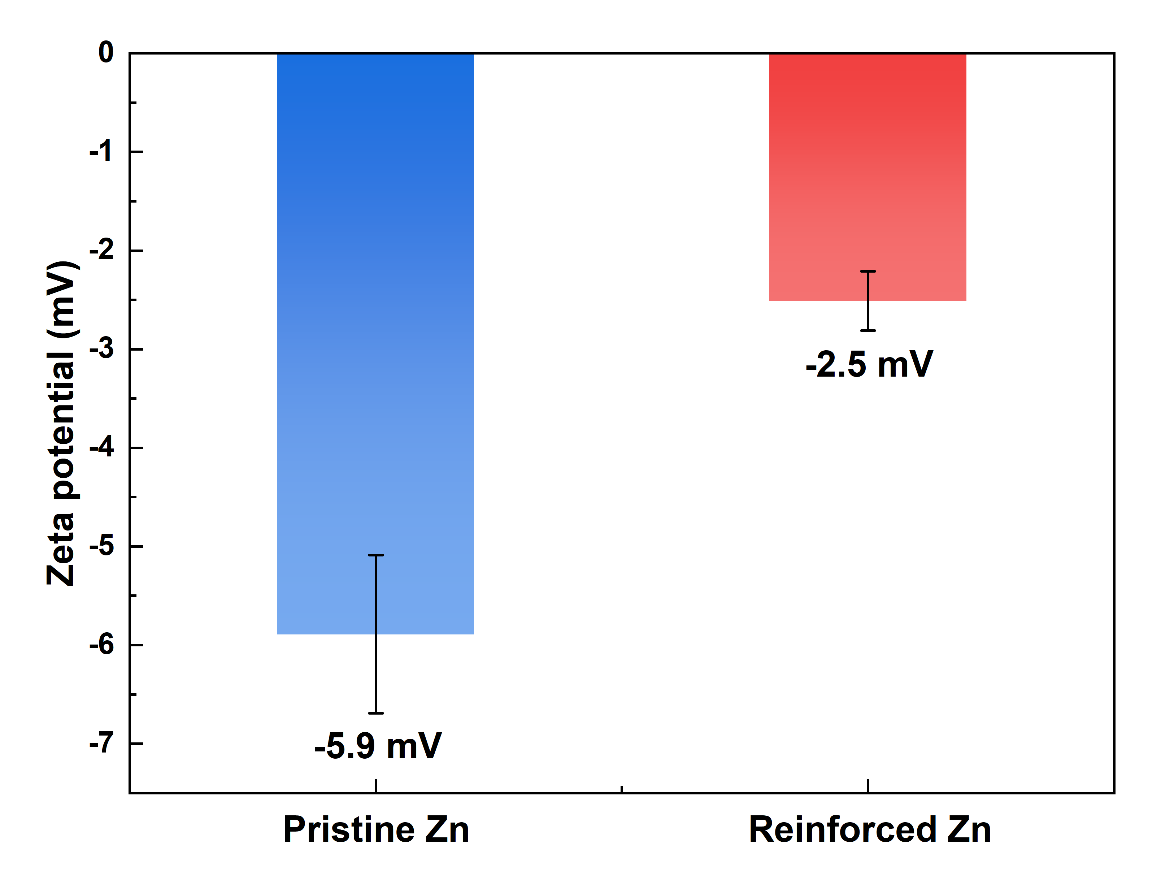


**Fig. S19** Zeta potential statistics of the Zn powder in the absence or at the presence of C_16_K (ZnSO_4_: 2.0 M, C_16_K: 0.10 mM)


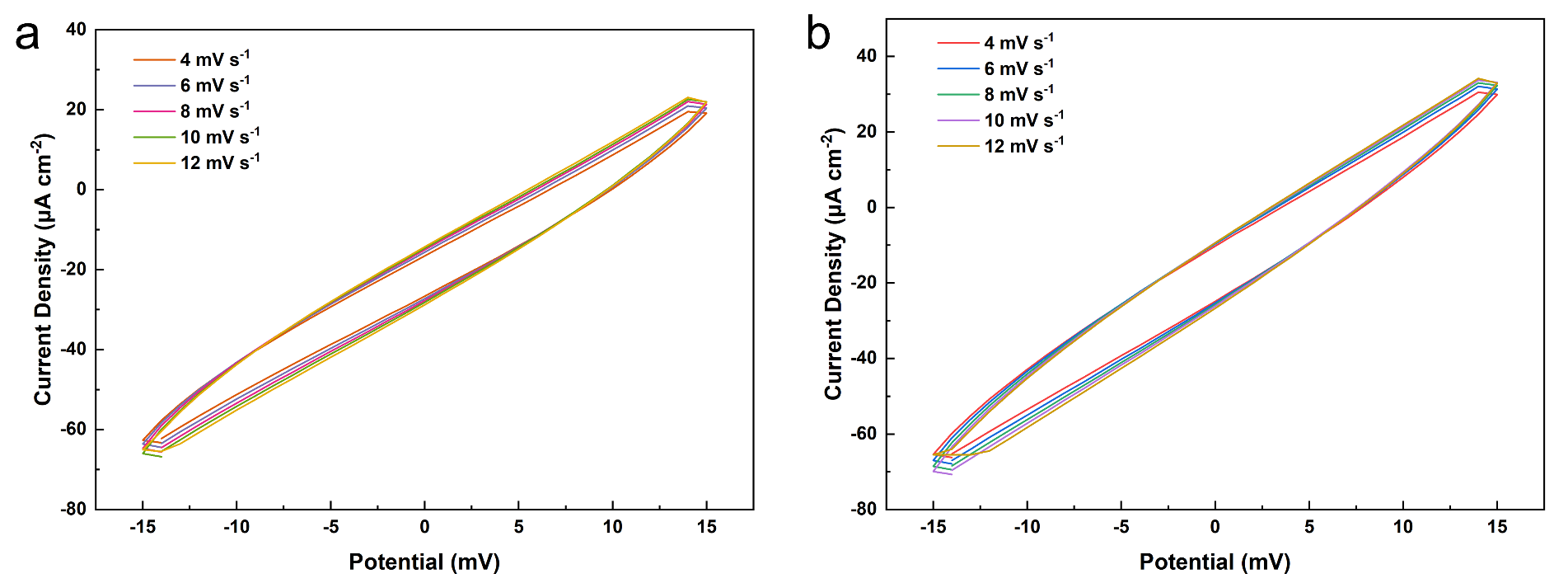


**Fig. S20** CV curves of the **a** pristine and **b** lipopeptide self-assembly reinforced Zn||Zn symmetric cells at various scan rates


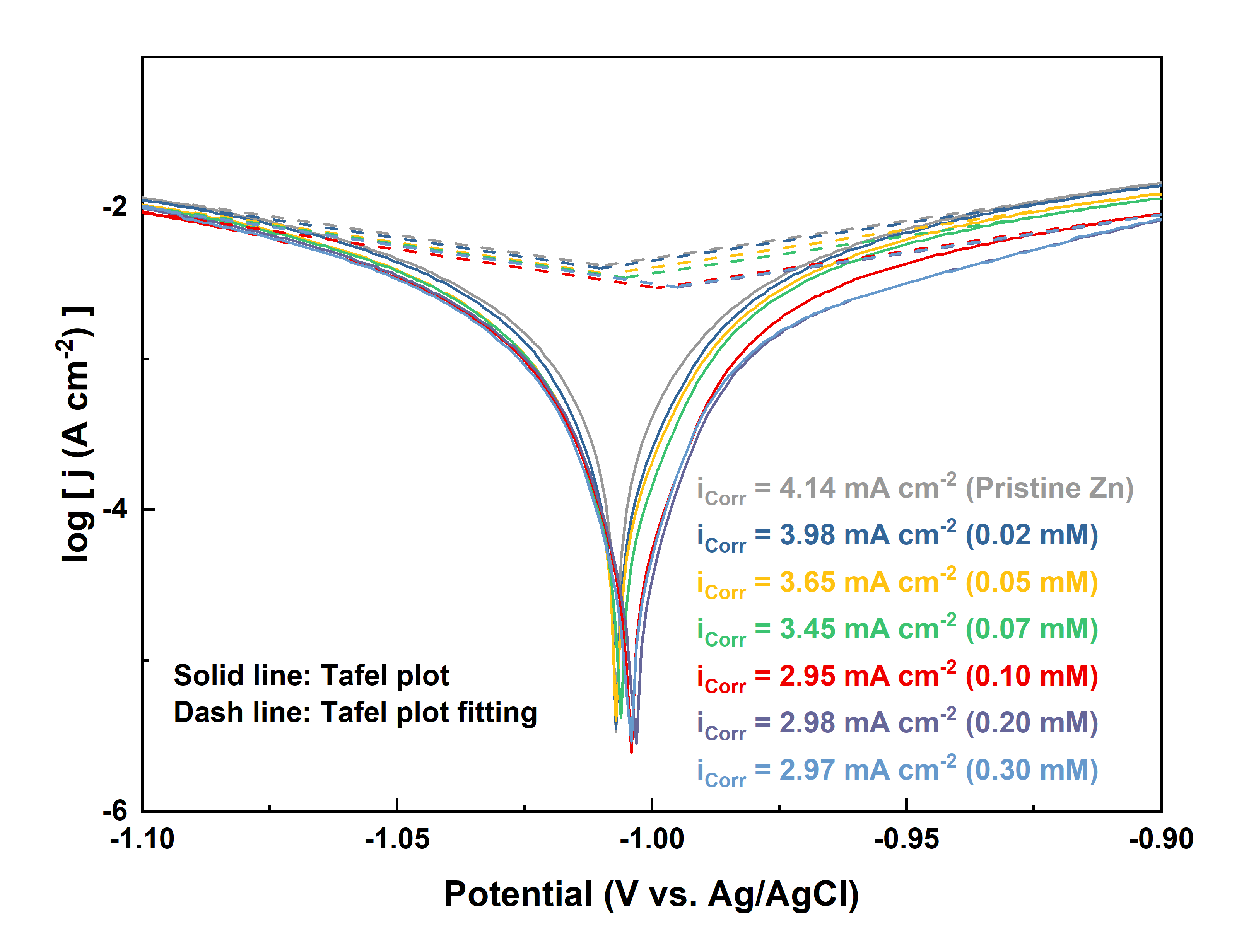


**Fig. S21** Tafel plots of the pristine Zn and C_16_K-modified Zn at varying concentrations


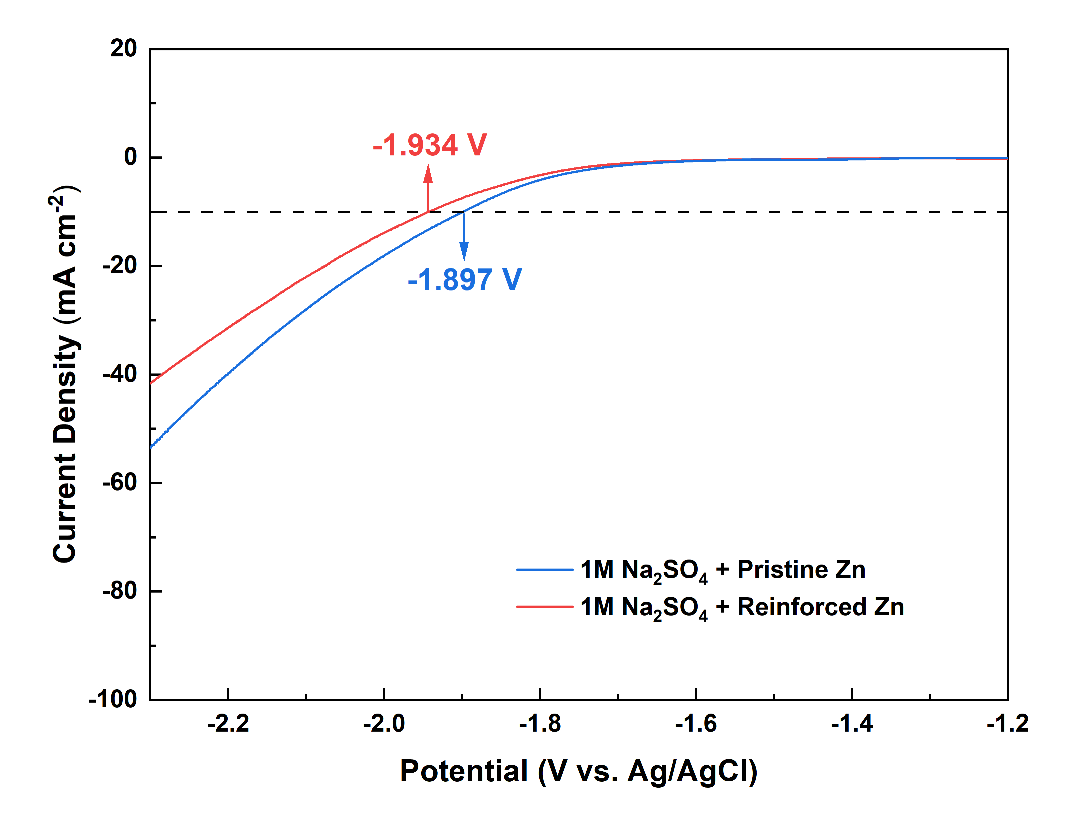


**Fig. S22** LSV curves of the Zn electrode tested in the absence or at the presence of C_16_K (ZnSO_4_: 2.0 M, C_16_K: 0.10 mM) using the three-electrode system. The values represent the initial HER potentials of the systems

**
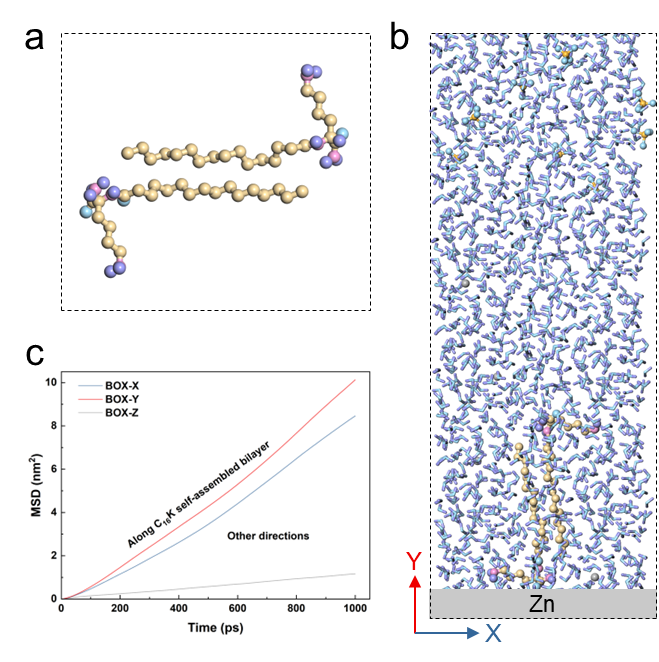
**

**Fig. S23** Migration behavior simulations of Zn^2+^ at the presence of the C_16_K self-assembled bilayers. **a,** Detailed view of the molecular structure of a pair of interdigitated lipopeptides in the self-assembled bilayer during the CGMD simulations. **b,** Snapshot of the interdigitated lipopeptide pair at the anode/electrolyte interface during the CGMD simulations. **c,** MSD of Zn^2+^ along the C_16_K self-assembled bilayer and in other directions from CGMD simulations


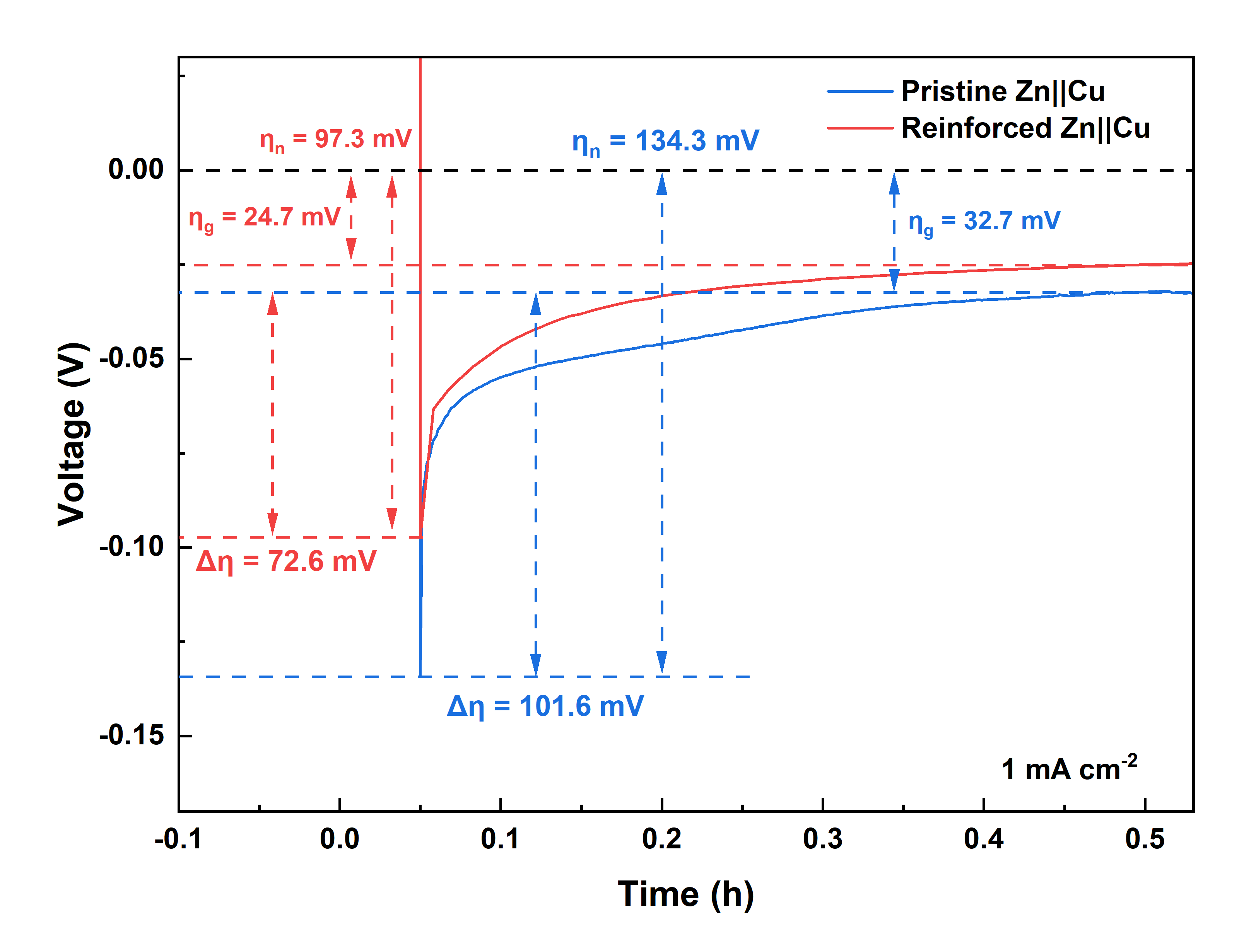


**Fig. S24** Nucleation overpotential and growth overpotential of Zn plating in Zn||Cu asymmetric cell in the absence or at the presence of C_16_K (ZnSO_4_: 2.0 M, C_16_K: 0.10 mM, current density: 1 mA cm^-2^)

The nucleation overpotential ($\eta_{n}$) is defined as the potential difference between the equilibrium potential (0 V) and the most negative potential at the onset of Zn plating, whereas the growth overpotential ($\eta_{g}$) is defined as the potential difference between the equilibrium potential and the subsequent quasi-steady plating potential. The overshoot $\Delta\eta$ is defined as ${\Delta\eta=\eta_{n}-\eta}_{g}$.


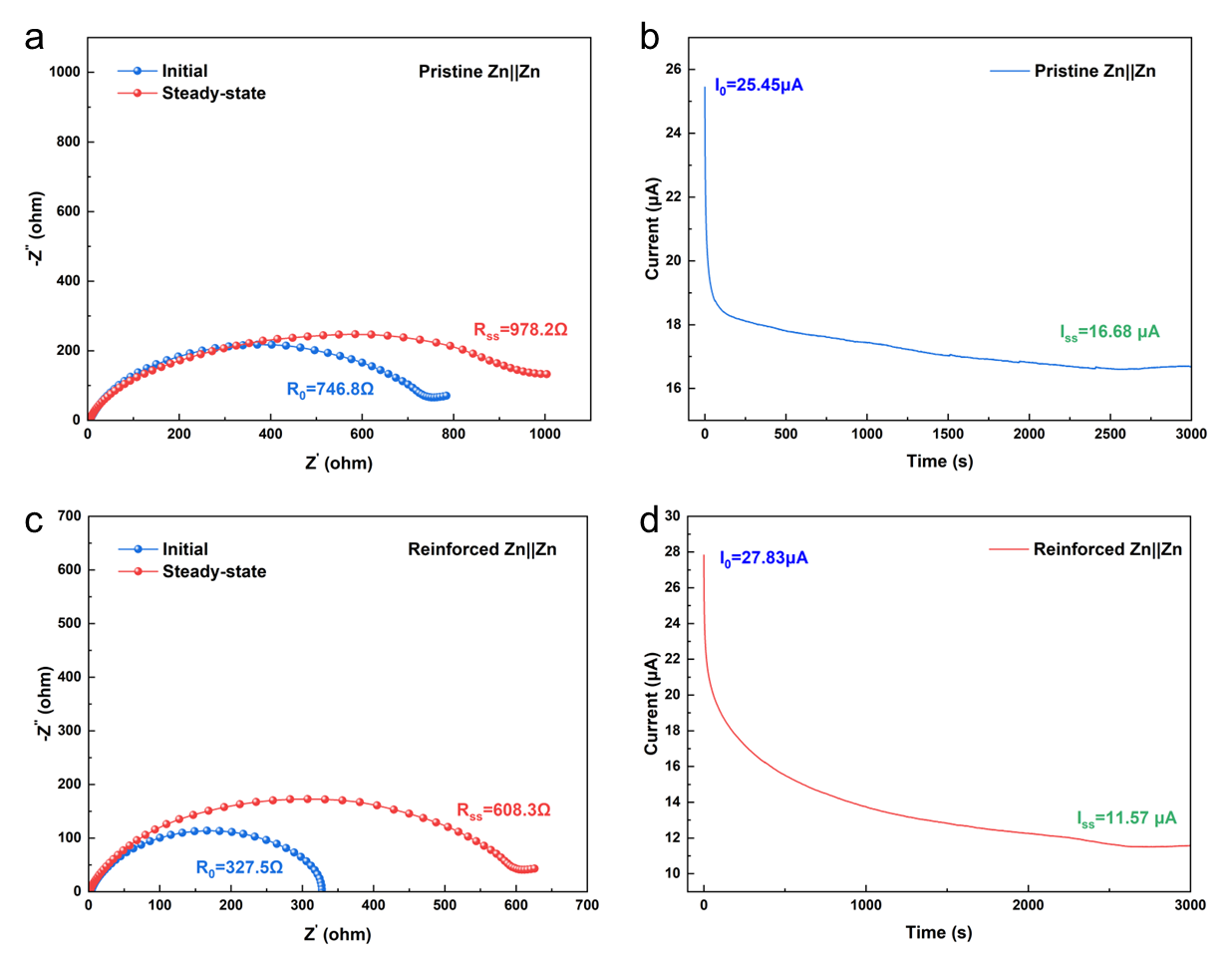


**Fig. S25** EIS profiles and corresponding amperometric i-t curves. **a and c,** EIS profiles of the Zn||Zn symmetric cells **a** in the absence or **c** at the presence of C_16_K (ZnSO_4_: 2.0 M, C_16_K: 0.10 mM). **b and d,** The corresponding amperometric *i*-*t* curves **b** in the absence or **d** at the presence of C_16_K under an applied overpotential of 20 mV


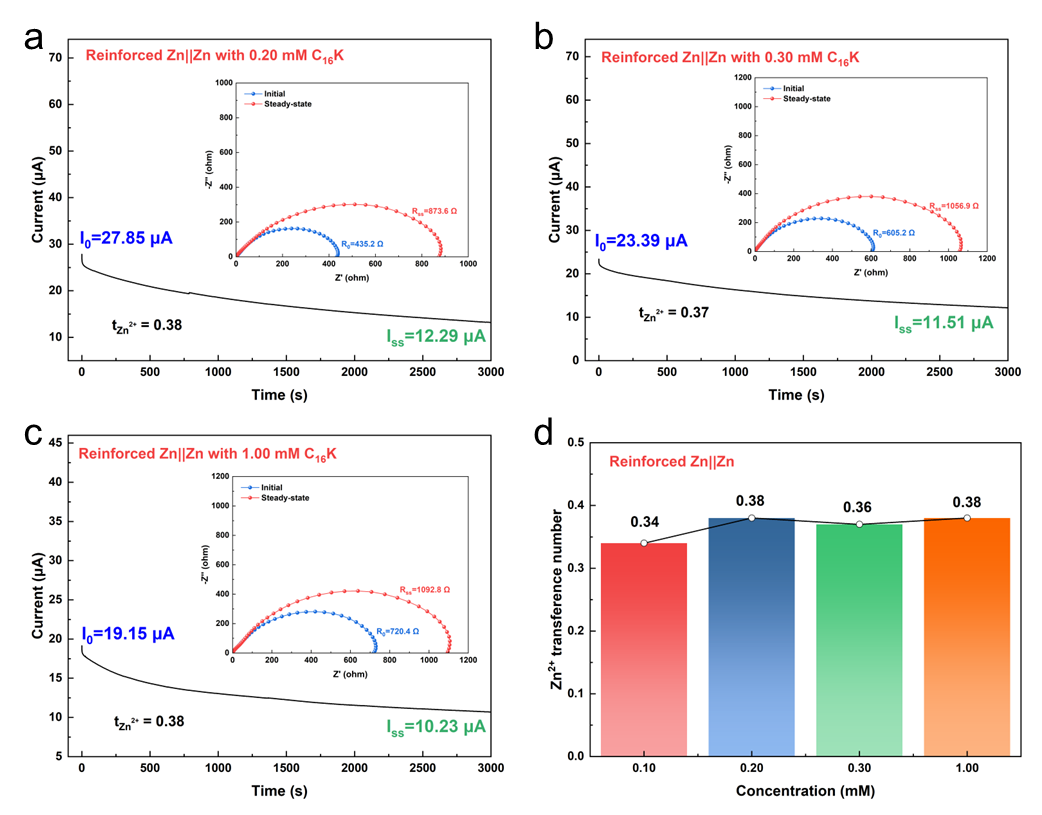


**Fig. S26** Zn^2+^ transference numbers of the reinforced Zn||Zn symmetric cells with different C_16_K concentrations. **a-c** Amperometric *i*-*t* curves of the reinforced Zn||Zn cells with (**a**) 0.20 mM, (**b**) 0.30 mM, and (**c**) 1.00 mM C_16_K, together with the EIS profiles recorded before and after polarization (insets). **d** Summary of the Zn^2+^ transference number at different C_16_K concentrations

**Table S3** Calculated Zn^2+^ transfer numbers ($t\left( {Zn}^{2+} \right)$) of the Zn||Zn symmetric cells

| **Cell** | **R_0_(ohm)** | **I_0_(μA cm^-2^)** | **R_ss_(ohm)** | **I_ss_(μA cm^-2^)** | **t_Zn_^2+^** |
| --- | --- | --- | --- | --- | --- |
| Pristine Zn\|\|Zn | 746.8 | 25.45 | 978.2 | 16.68 | 0.18 |
| Reinforced Zn\|\|Zn with 0.10 mM C_16_K | 327.5 | 27.83 | 608.3 | 11.57 | 0.34 |


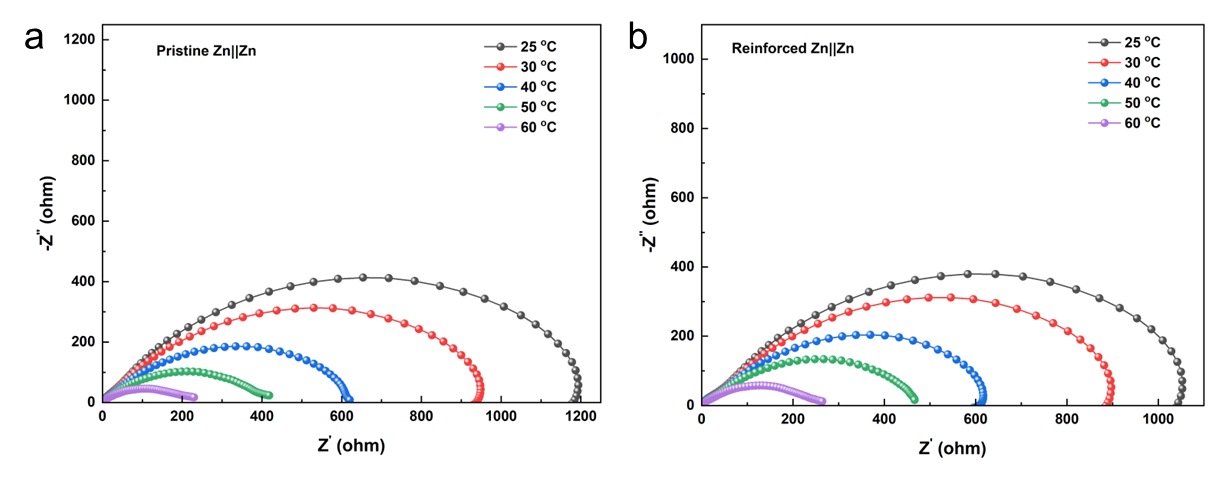


**Fig. S27** Nyquist plots of the Zn||Zn symmetric cells tested under various temperatures **a** in the absence or **b** at the presence of C_16_K


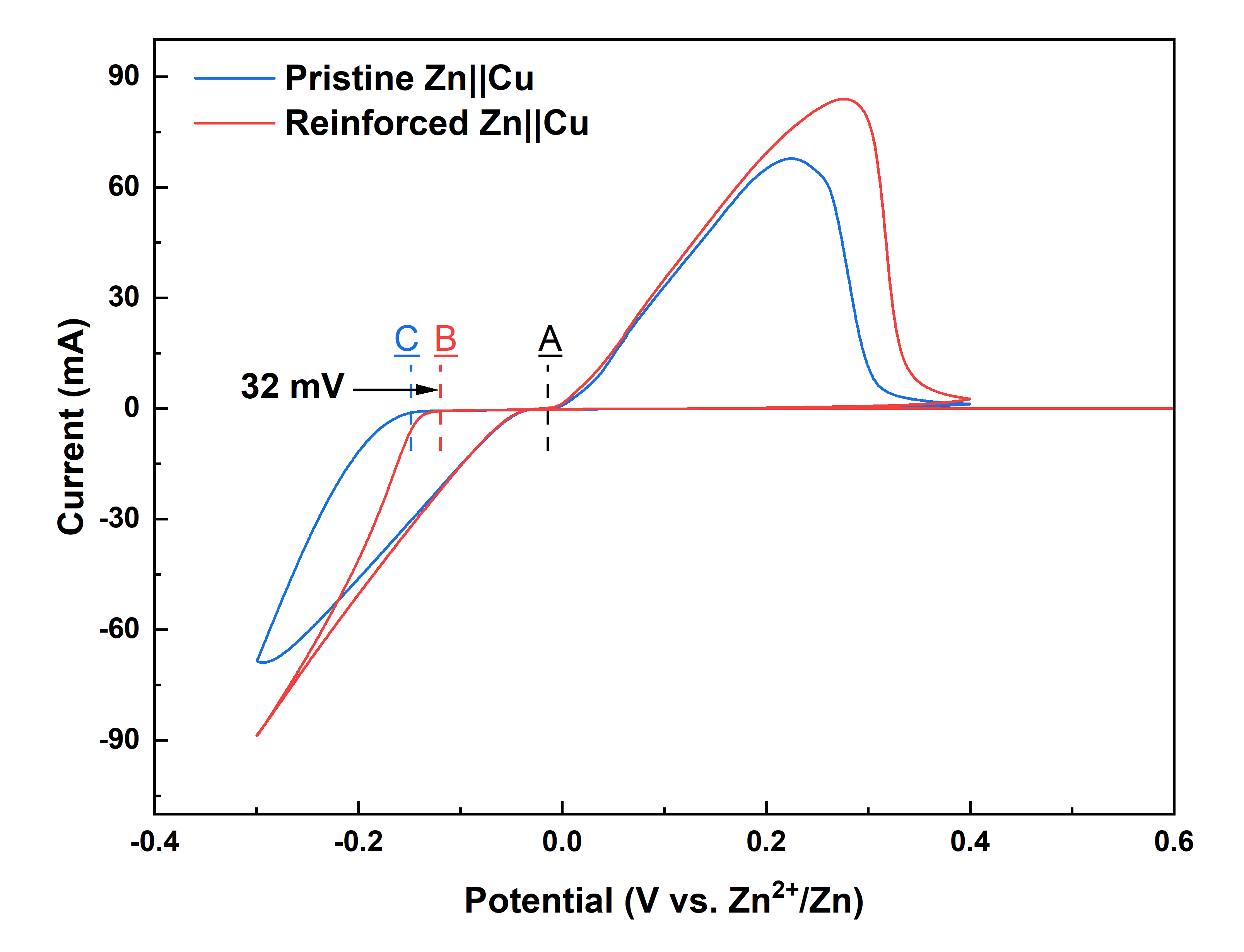


**Fig. S28** CV curves of the Zn||Cu asymmetric cells in the absence or at the presence of C_16_K (ZnSO_4_: 2.0 M, C_16_K: 0.10 mM, current density: 1 mA cm^-2^, scan rate: 2 mV s^-1^). Point A denotes the crossover potential, while points B and C indicate the onset potential of Zn^2+^ deposition reactions


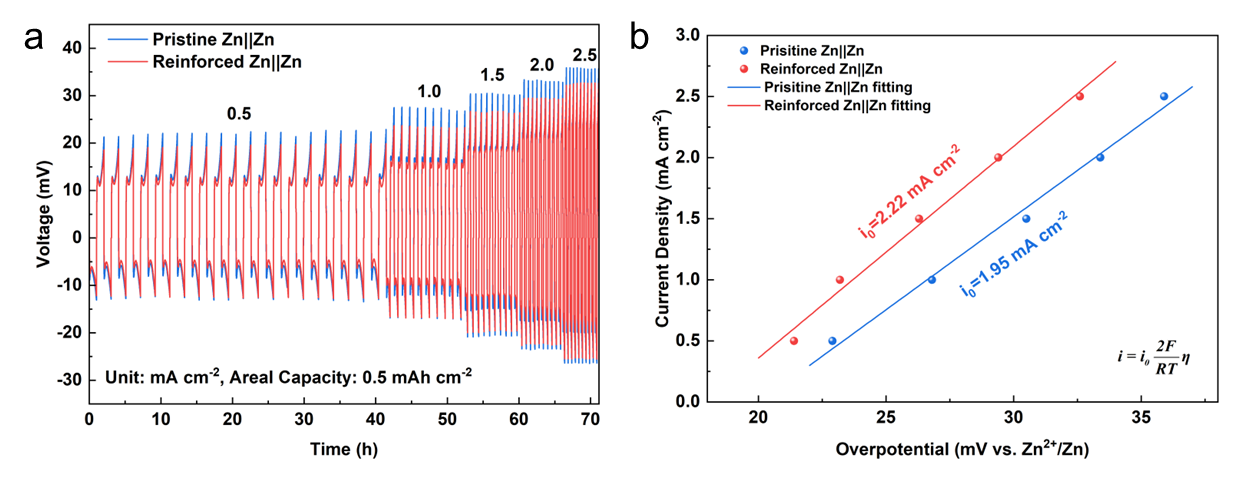


**Fig. S29** Exchange current density measurements of the Zn||Zn symmetric cells tested in the absence or at the presence of C_16_K. **a,** Galvanostatic cycling performances under current densities ranging from 0.5 to 2.5 mA cm⁻². **b,** Exchange current density plots derived from the Zn plating/stripping measurements


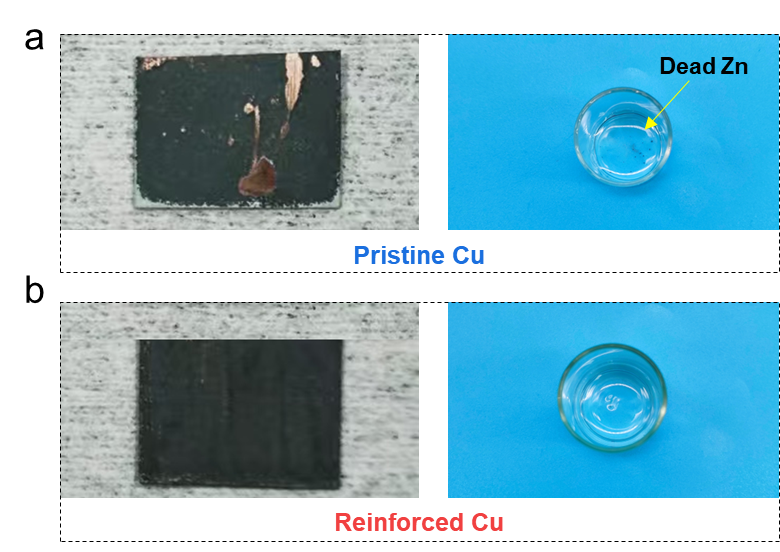


**Fig. S30** Optical images of the Zn deposited on Cu foils and corresponding electrolytes **a** in the absence or **b** at the presence of C_16_K (ZnSO_4_: 2.0 M, C_16_K: 0.10 mM, test conditions: 5 mA cm^-2^ for 1 h)


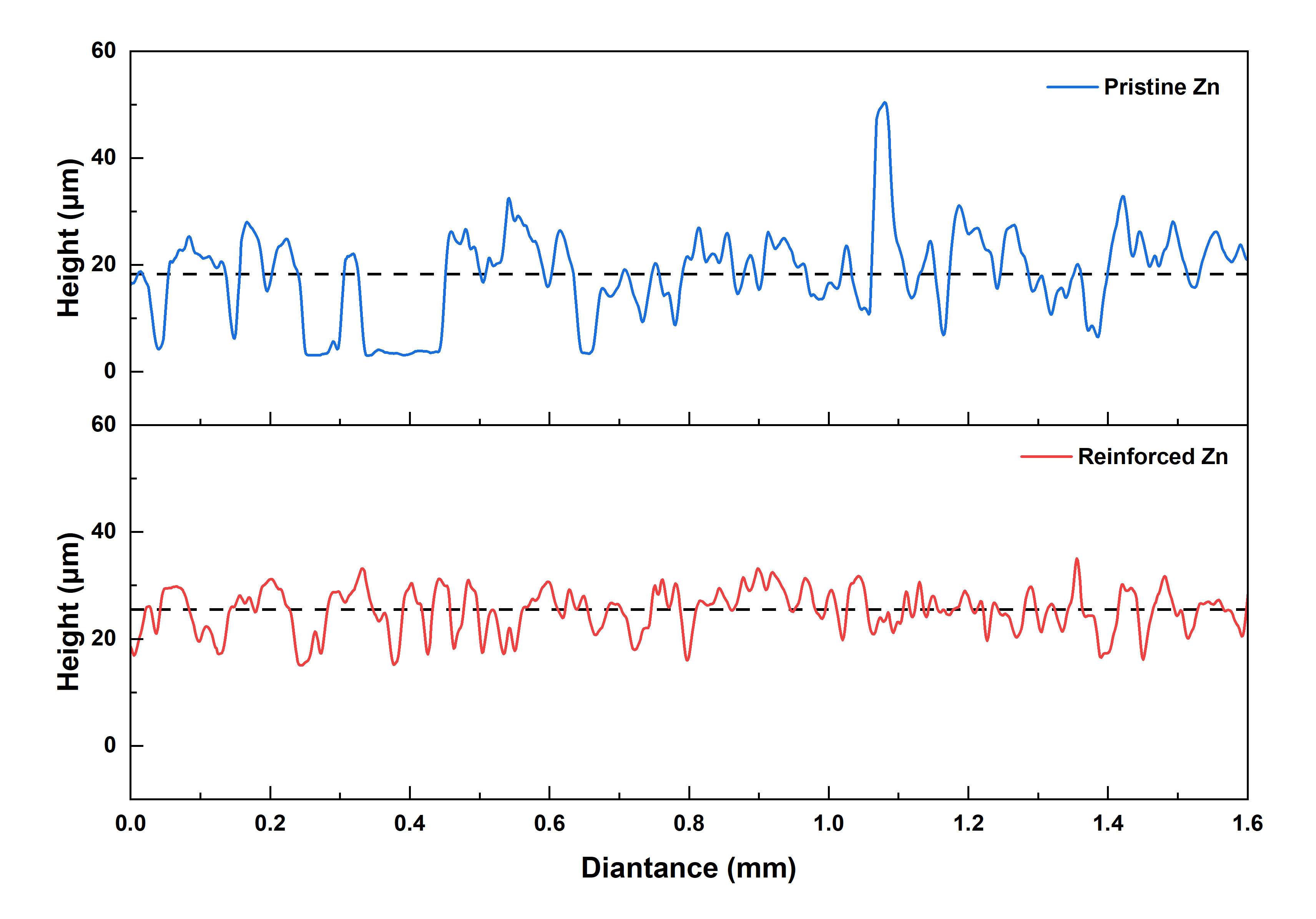


**Fig. S31** Line roughness analysis. Surface profiles extracted along the white dotted lines in Fig. 3e in the main text


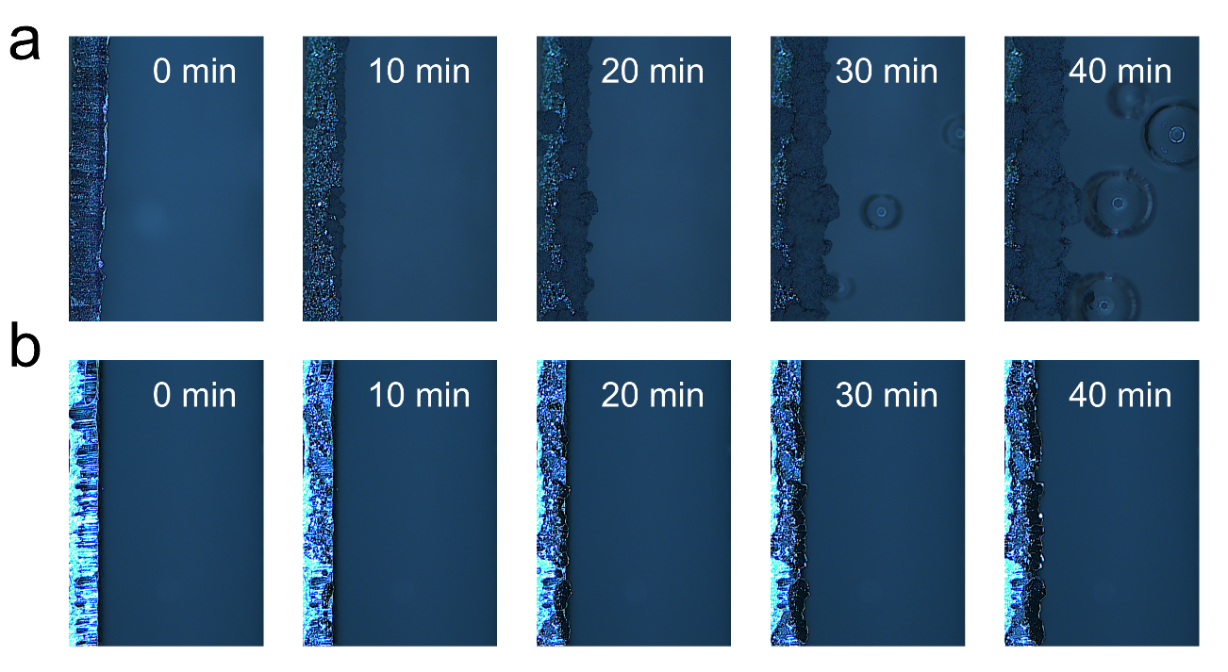


**Fig. S32** Time-evolved *in-situ* optical microscopy images of the Zn electrodes during Zn plating in the Zn||Zn symmetric cells. **a,** In the absence of C_16_K. **b,** At the presence of C_16_K. (ZnSO_4_: 2.0 M, C_16_K: 0.10 mM, test conditions: 1 mA cm^−2^ & 1 mAh cm^−2^)


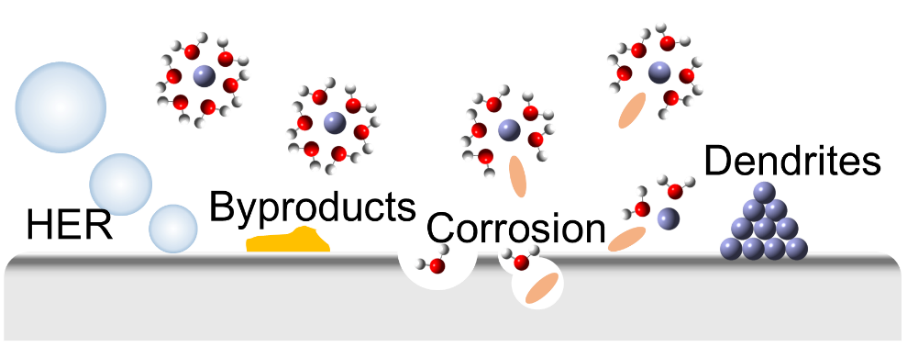


**Fig. S33** Schematic illustration of Zn plating in native ZnSO_4_ electrolyte, showing intense byproducts formation and HER side reactions

**T****able S4** Comparison of the electrochemical performances in diverse Zn||Zn symmetric cells with different treatment strategies.

| **Strategy** | | **Current density**  **(mA cm^-2^)** | **Areal capacity**  **(mAh cm^-2^)** | **Cycle lifespan**  **(h)** | **CPC**  **(Ah cm^-2^)** | **Ref.** |
| --- | --- | --- | --- | --- | --- | --- |
| Eelectrolyte additives | Synergistic reinforcement of ion transport and interfacial stability | 1 | 1 | 4000 | 2 | This work |
|  |  | 5 | 5 | 2250 | 5.625 |  |
|  |  | 10 | 5 | 1020 | 5.1 |  |
|  | HSE-10 m | 0.5 | 0.5 | 1200 | 0.3 | [49] |
|  | BE/5Cor | 10 | 10 | 600 | 3 | [50] |
|  | ZnSO_4_-C_3_N_4_QDs | 5 | 1 | 400 | 1 | [51] |
|  | HDES | 1 | 1 | 4500 | 2.25 | [52] |
|  | 0.5 M Zn(HBS)_2_ | 5 | 1 | 1600 | 4 | [53] |
|  | 0.05 M BmBr/ZSO | 1 | 1 | 2700 | 1.35 | [54] |
|  | 0.1-LBG | 2 | 2 | 1160 | 1.16 | [55] |
|  | ZnSO_4_-1% Py | 0.5 | 0.5 | 3300 | 0.825 | [56] |
|  | SP-ZnCl_2_ | 5 | 2 | 1200 | 3 | [57] |
|  | ZnOTF/MAAC | 4 | 4 | 700 | 1.4 | [58] |
| Gel electrolytes | Asymmetric electrolyte | 10 | 10 | 700 | 3.5 | [59] |
|  | ZIG-20wt% | 1 | 0.5 | 900 | 0.45 | [60] |
| Coatings | Zn@PAH/PAA | 5 | 5 | 340 | 0.85 | [61] |
|  | CN10@Zn | 4 | 2 | 1100 | 2.2 | [62] |
| Separators | HTS | 2 | 2 | 3000 | 3 | [63] |


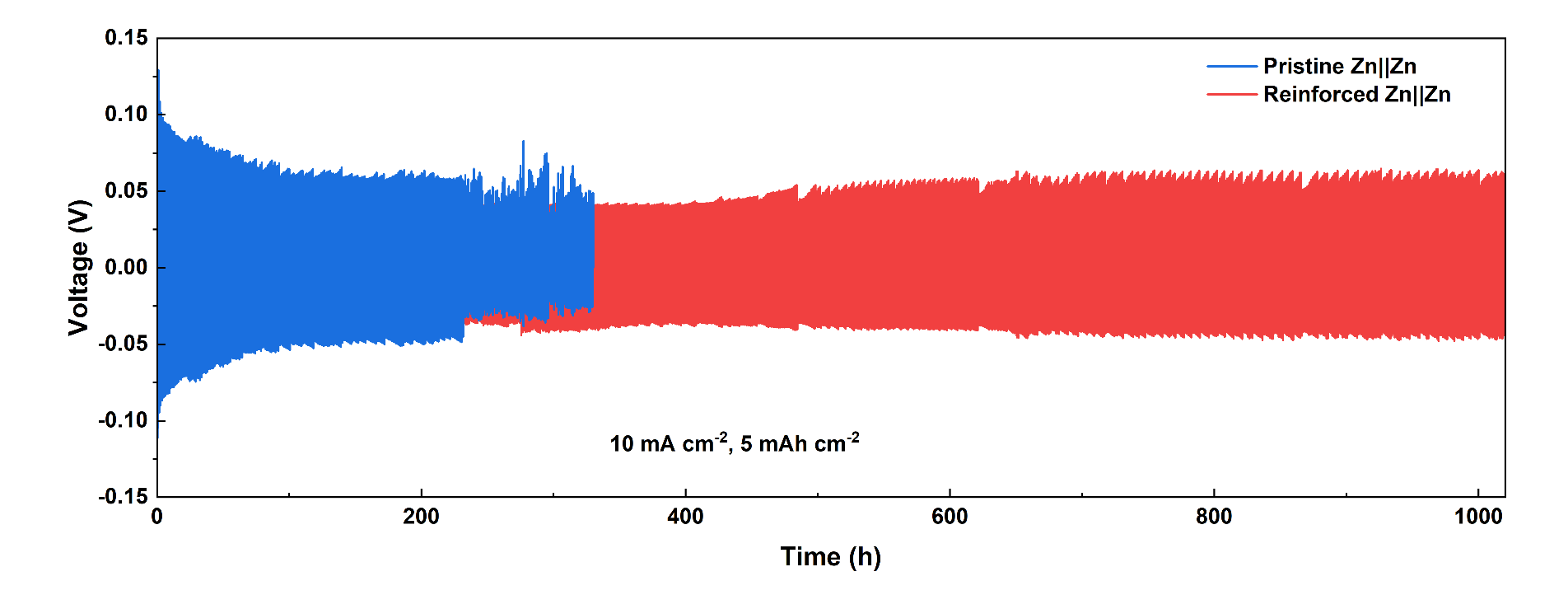


**Fig. S34** Voltage profiles of the pristine (blue) and reinforced (red) Zn||Zn symmetric cells at a current density of 10 mA cm^-2^ for 5 mAh cm^-2^


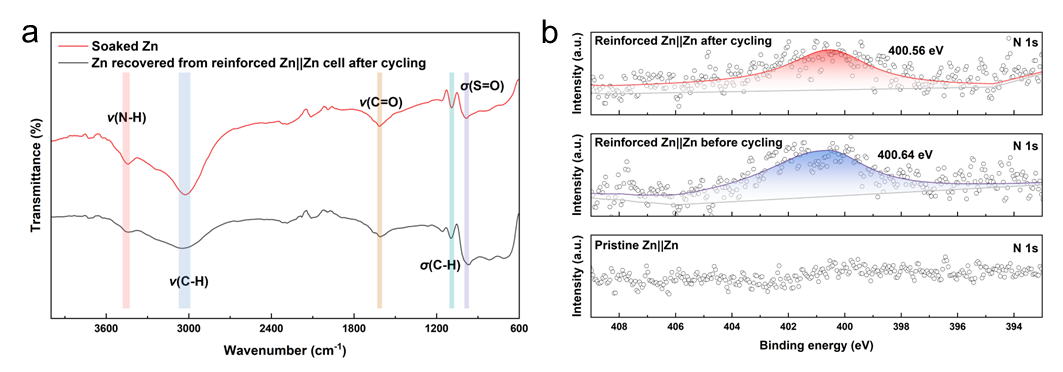


**Fig. S35 a** FTIR spectra of the Zn soaked in the ZnSO_4_ electrolyte at the presence of C_16_K and Zn recovered from reinforced Zn||Zn cell after cycling. **b** High-resolution N1s XPS spectra of Zn recovered from pristine Zn||Zn and reinforced Zn||Zn cells before and after cycling


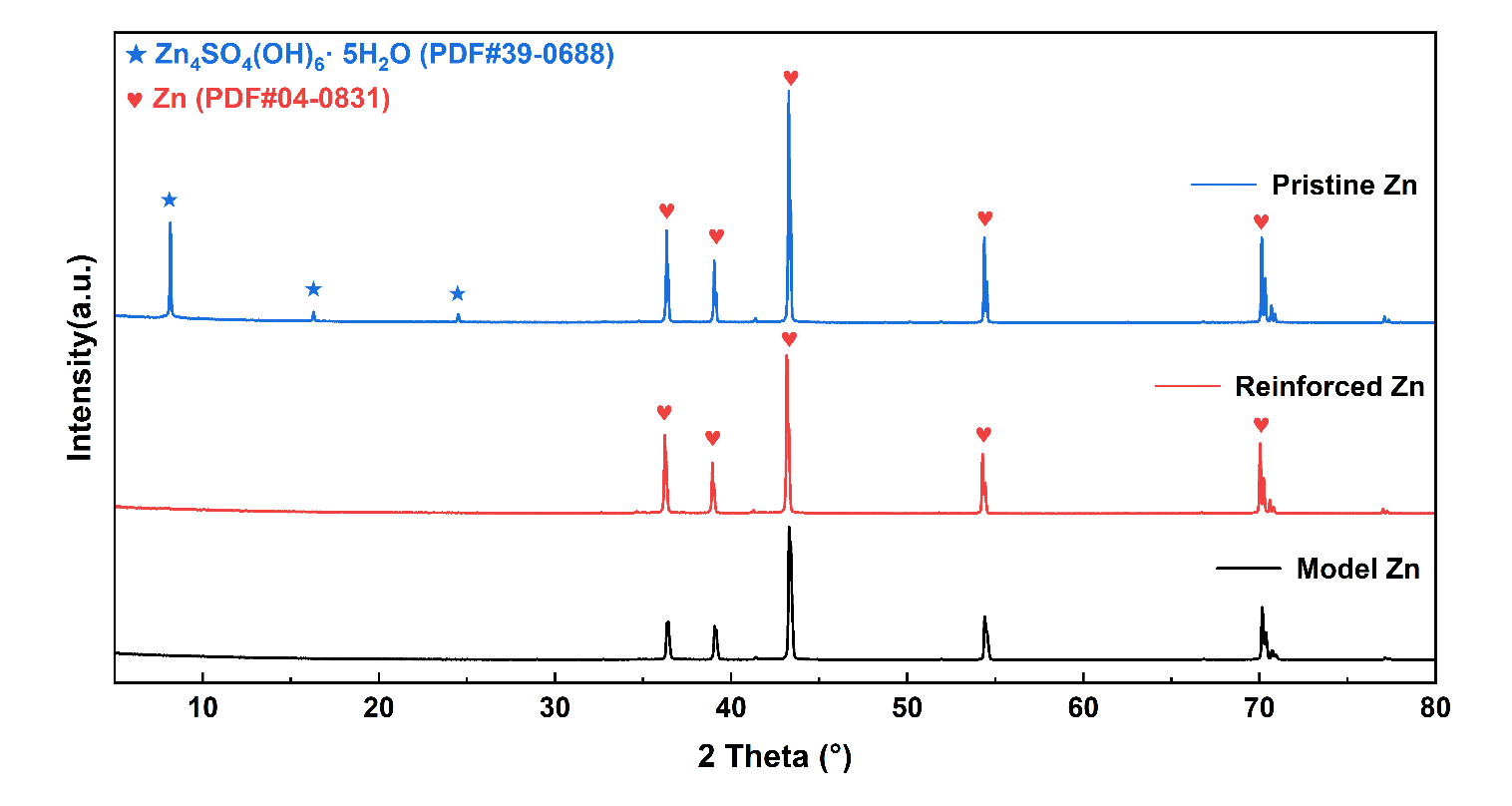


**Fig. S36** XRD patterns of the pristine and reinforced Zn anodes after cycling for 50 cycles at 1 mA cm^-2^ for 1 mAh cm^-2^


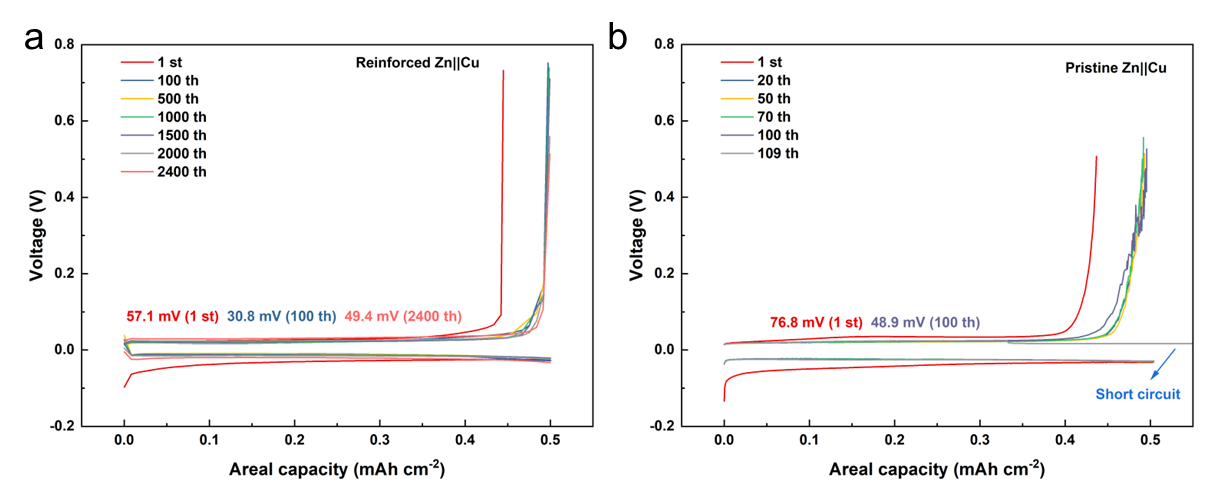


**Fig. S37** Representative capacity-voltage profiles of the **a** reinforced and **b** pristine Zn||Cu asymmetric cells at a current density of 1 mA cm^-2^ for 0.5 mAh cm^-2^


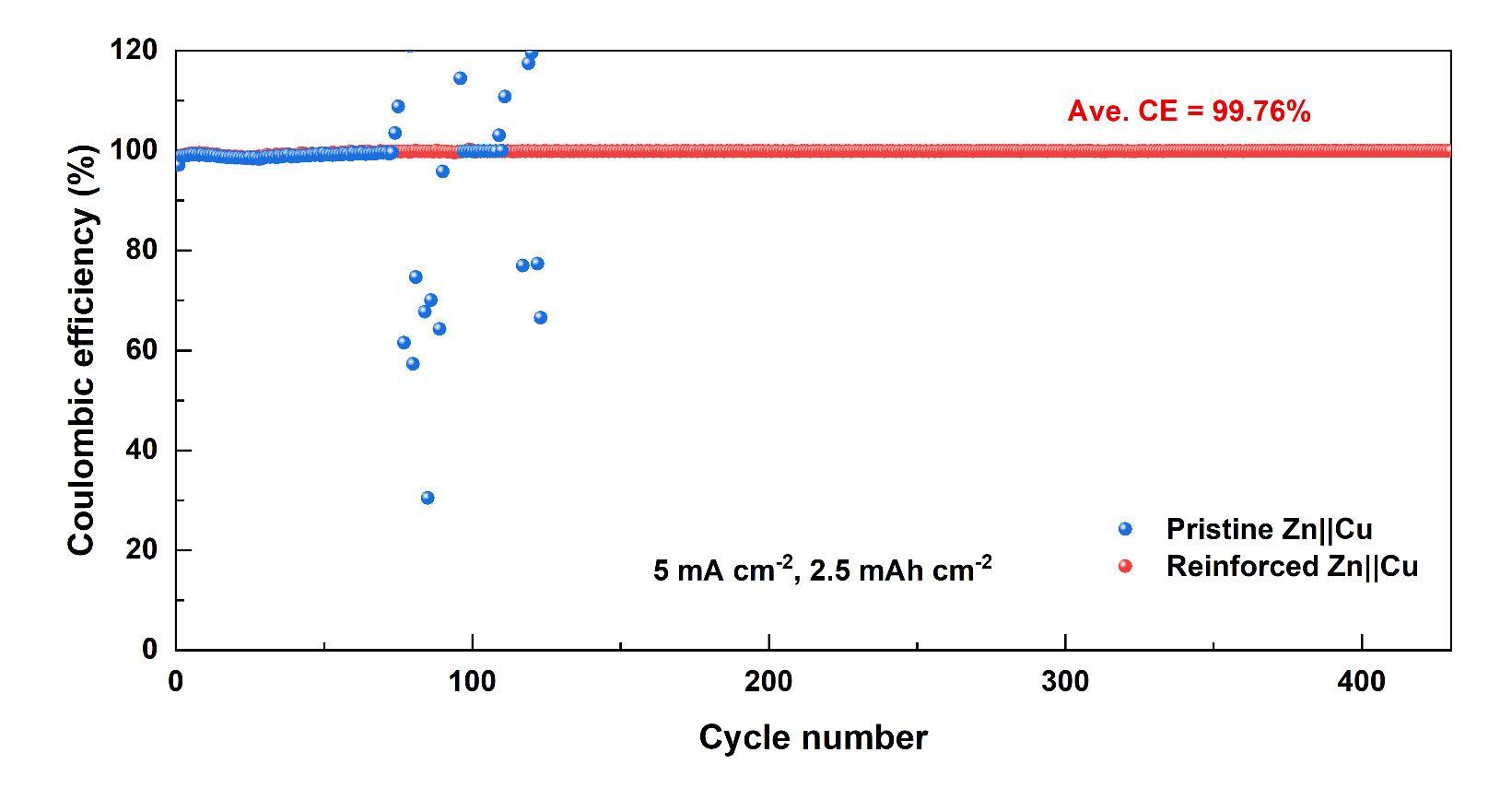


**Fig. S38** CE comparison of the reinforced (red) and pristine (blue) Zn||Cu asymmetric cells at 5 mA cm^-2^ for 2.5 mAh cm^-2^


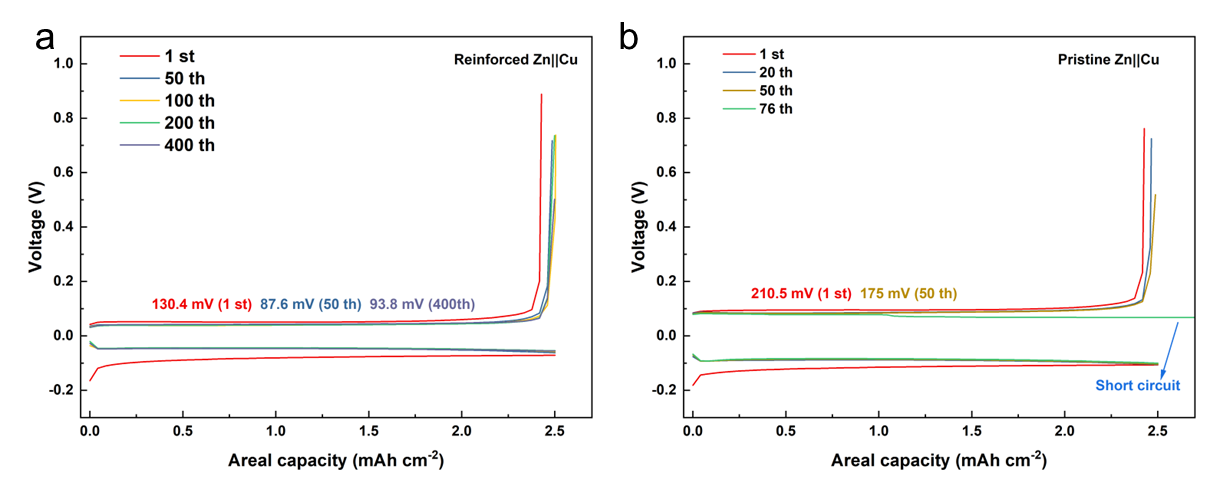


**Fig. S39** Representative capacity-voltage profiles of the **a** reinforced and **b** pristine Zn||Cu asymmetric cells at a current density of 5 mA cm^-2^ for 2.5 mAh cm^-2^


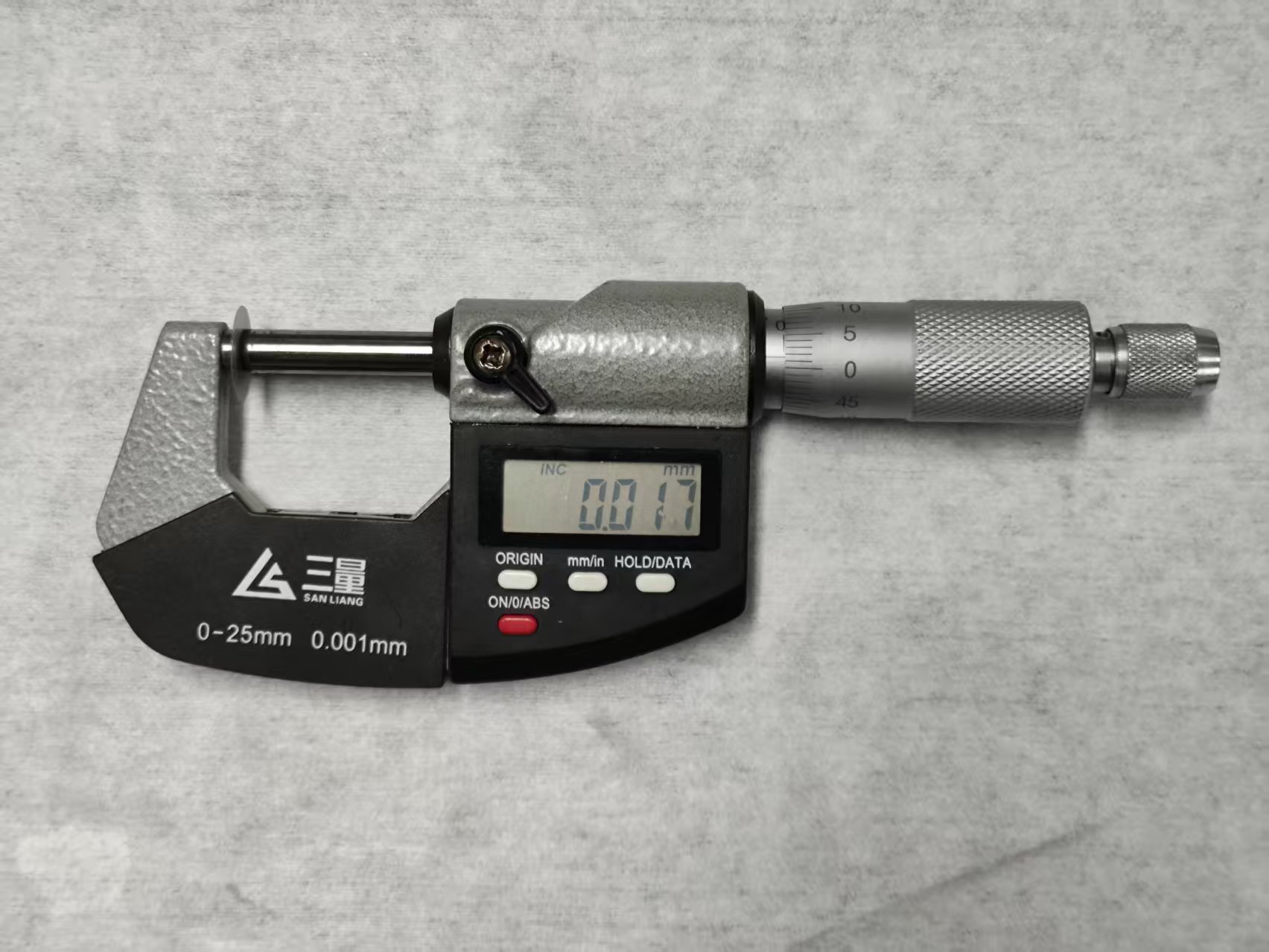


**Fig. S40** Measurement of the thickness of the Zn foil employed in the experiment at a high Zn utilization rate of 50% DoD


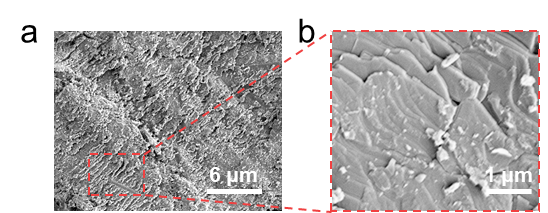


**Fig. S41** SEM images of the Zn anode after 300 h of cycling at a high Zn utilization rate of 50% DoD. **b** is a magnified view of the selected region in **a**


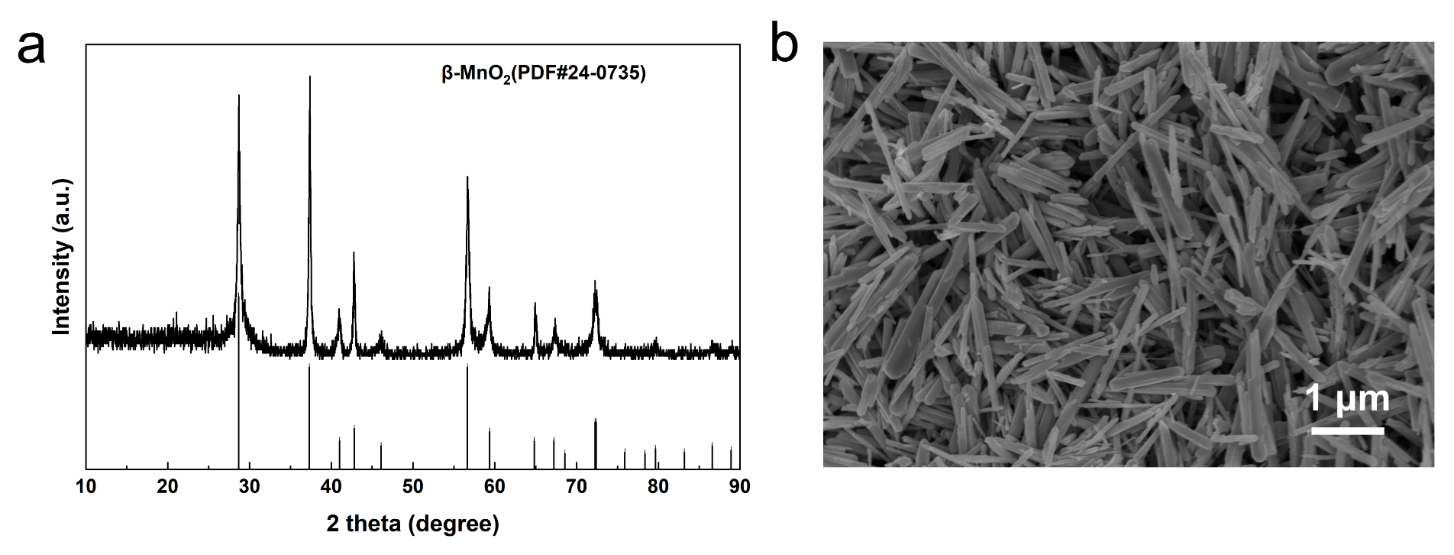


**Fig. S42 a** XRD pattern and **b** SEM image of the synthesized β-MnO_2_ cathode (PDF#24-0735) by a hydrothermal method. The results demonstrate that the cathode material possessed needle-like morphologies and high crystallinity with the characteristic peaks of β-MnO_2_


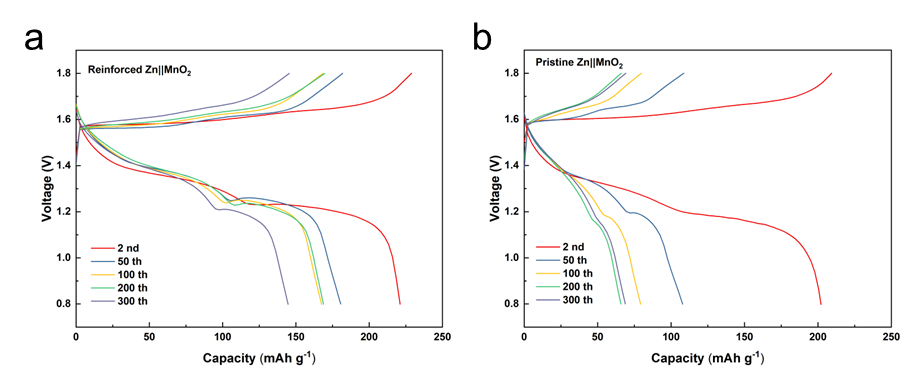


**Fig. S43** The charge/discharge profiles of the **a** reinforced and **b** pristine Zn||MnO_2_ full cells at 1.0 A g^-1^. The charge/discharge profiles of the C_16_K self-assembly reinforced Zn||MnO_2_ full cells exhibit a more stable discharge plateau with lower voltage hysteresis contrast to the untreated counterpart


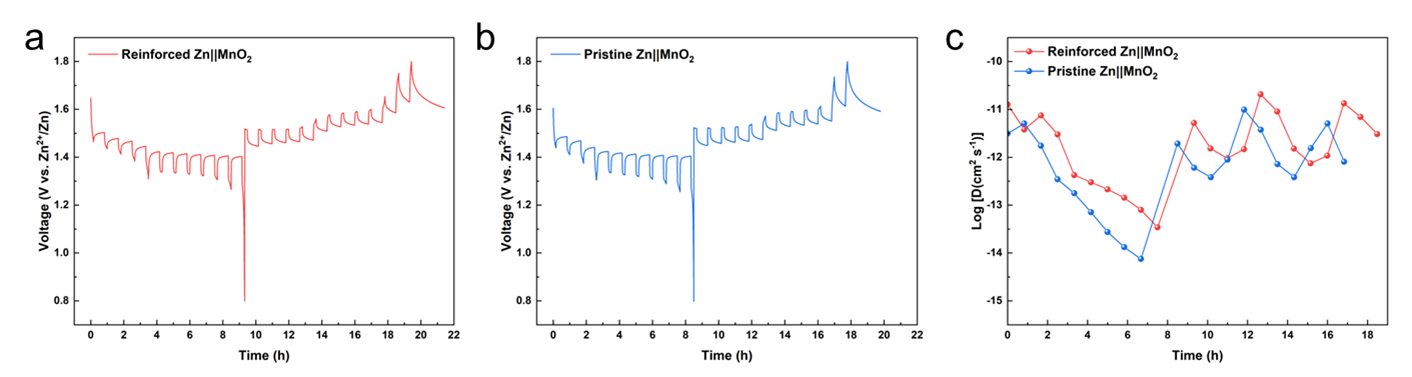


**Fig. S44** GITT curves of (**a)** reinforced and (**b)** pristine Zn||MnO_2_ cells at 0.1 A g^-1^, and (**c)** the corresponding diffusion coefficient values calculated for both cells


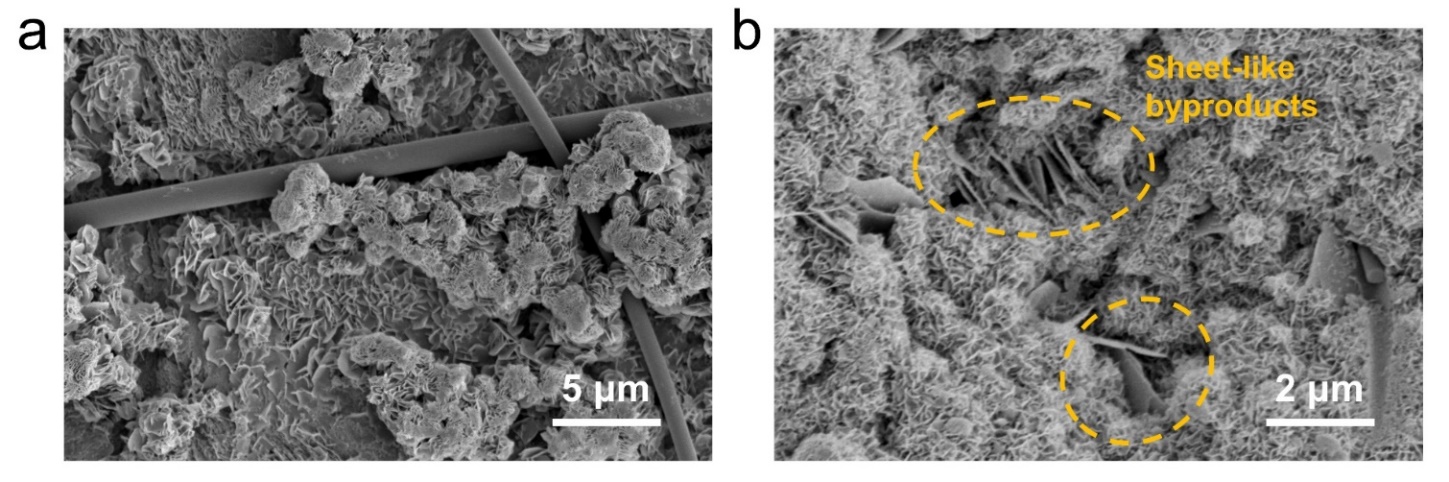


**Fig. S45** SEM images of the **a** Zn anode and **b** MnO_2_ cathode in the pristine Zn||MnO_2_ full cells after 50 cycles


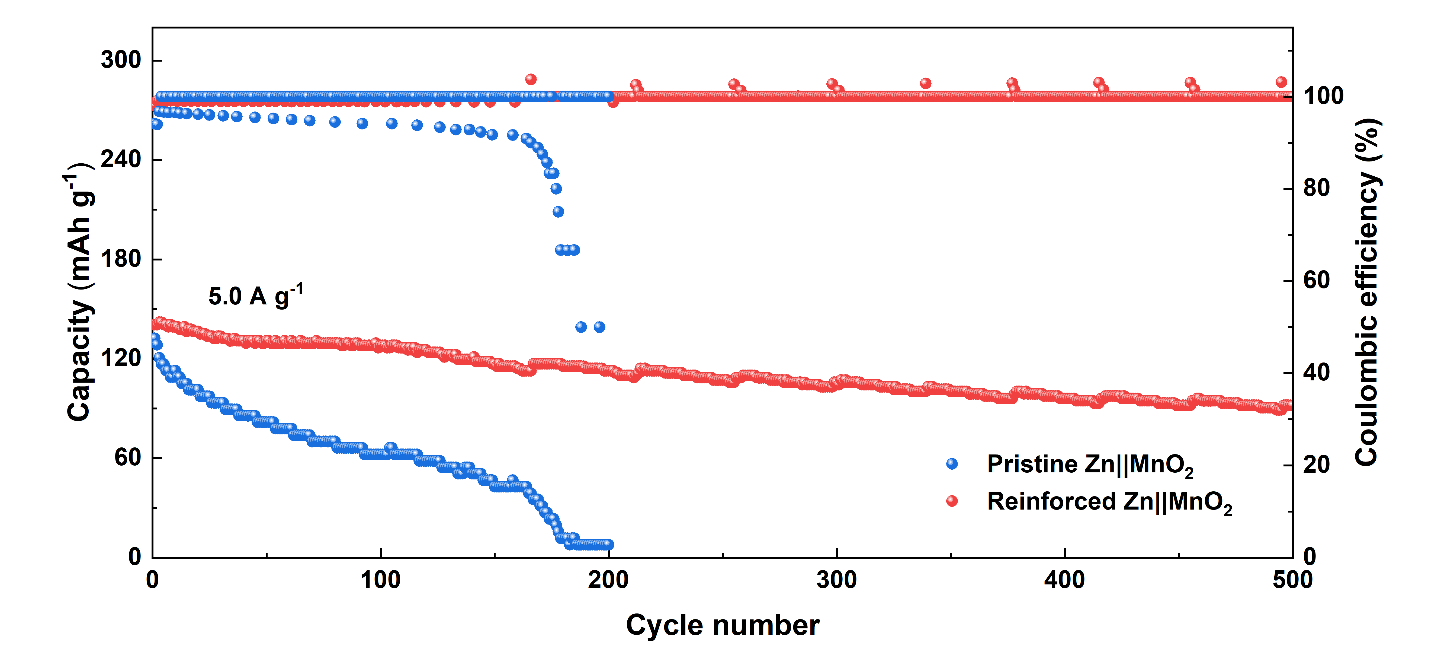


**Fig. S46** Long-term cycling performance of the (red) reinforced and (blue) pristine Zn||MnO_2_ full cells at 5.0 A g^-1^


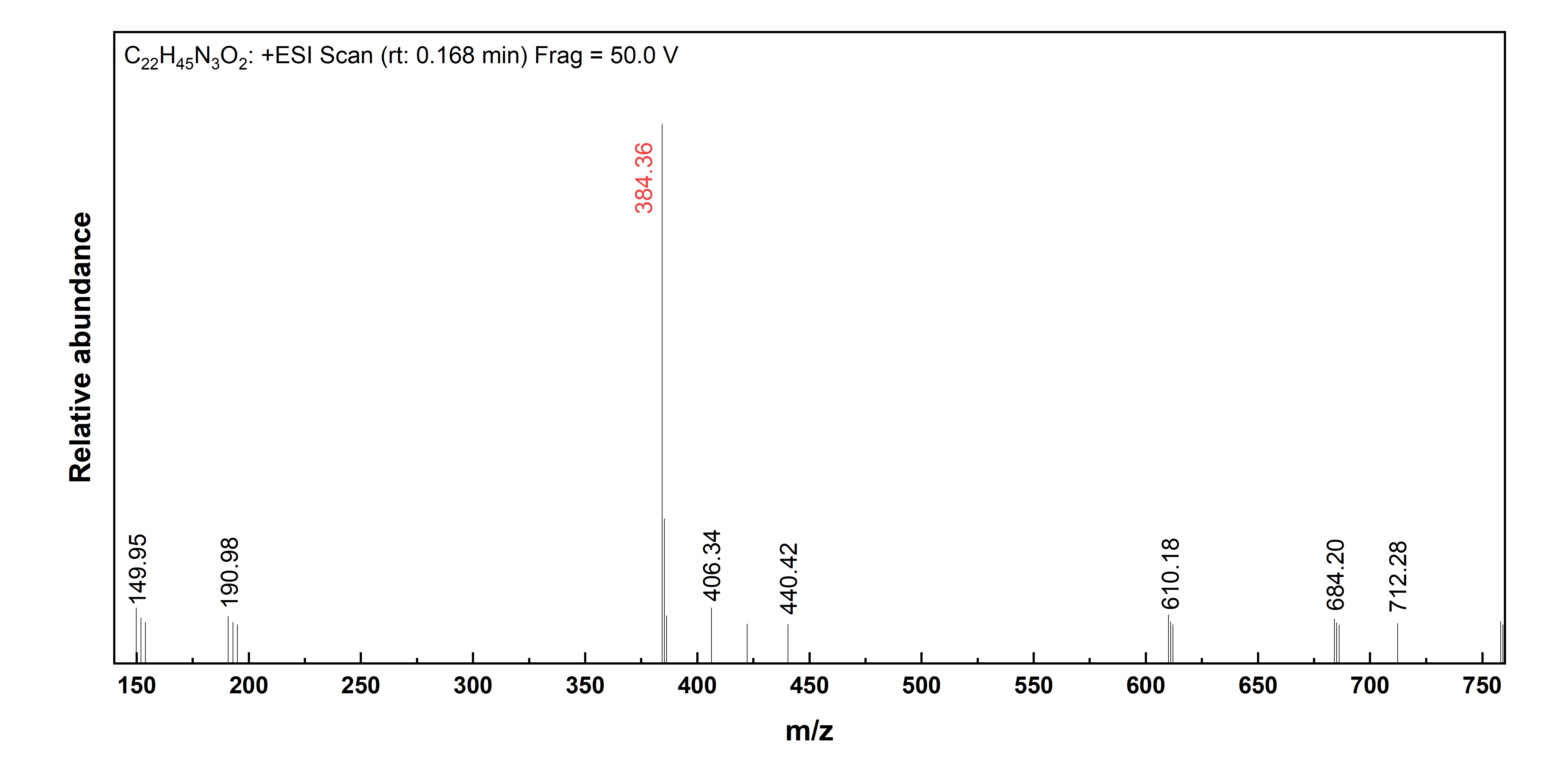


**Fig. S47** MS spectrum of the electrolyte extracted from the reinforced Zn||MnO_2_ cell after charging to 1.8 V, showing a predominant peak at *m/z* 384.36 corresponding to the [M+H]^+^ of C_16_K


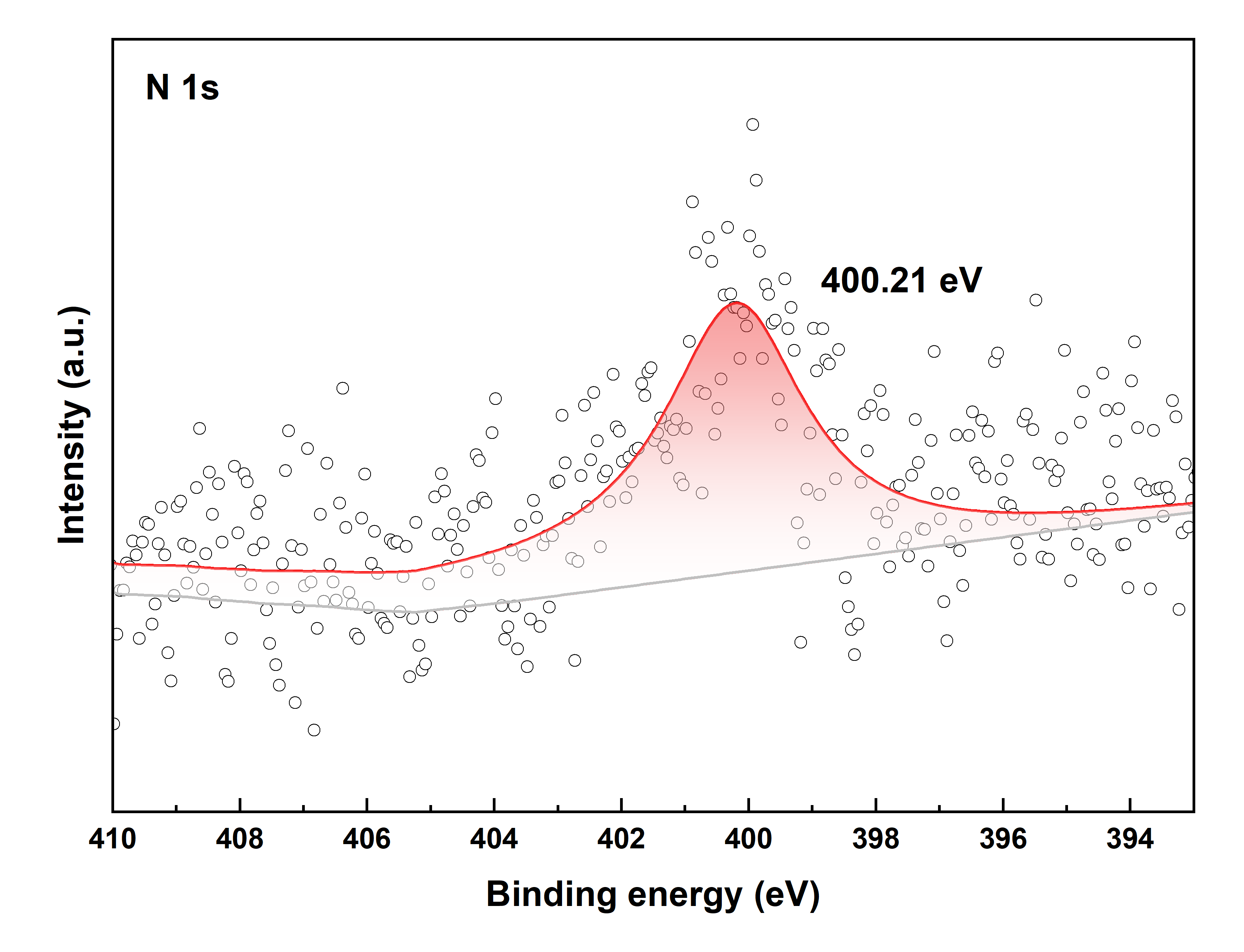


**Fig. S48** High-resolution N1s XPS spectrum of the cathode from the reinforced Zn||MnO_2_ cell after charging to 1.8 V


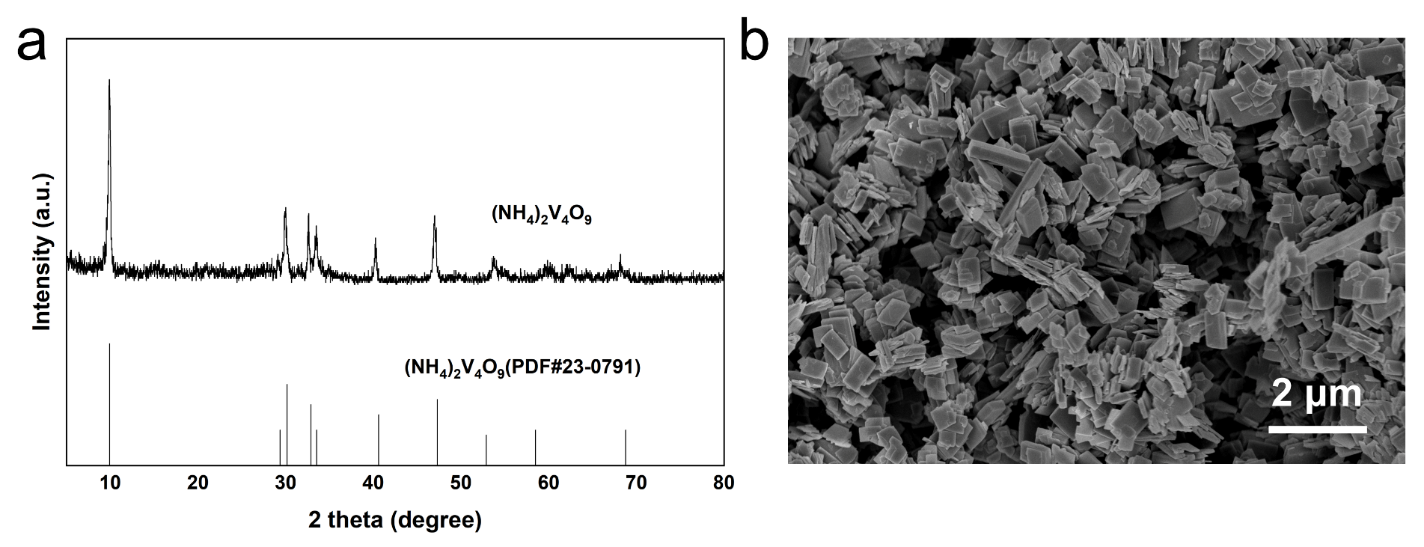


**Fig. S49 a** XRD pattern and **b** SEM image of the (NH_4_)_2_V_4_O_9_ cathode (PDF#23-0791) synthesized by a hydrothermal method. The results demonstrate that the cathode material possessed lamellar morphologies and high crystallinity with the typical peaks of (NH_4_)_2_V_4_O_9_


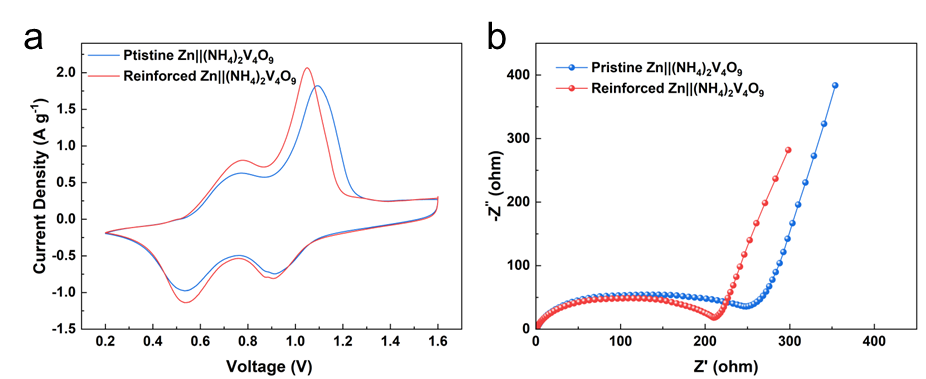


**Fig. S50 a** CV curves and **b** EIS of the (red) C_16_K self-assembly reinforced and (blue) pristine Zn||(NH_4_)_2_V_4_O_9_ full cells at a scan rate of 0.5 mV s^-1^


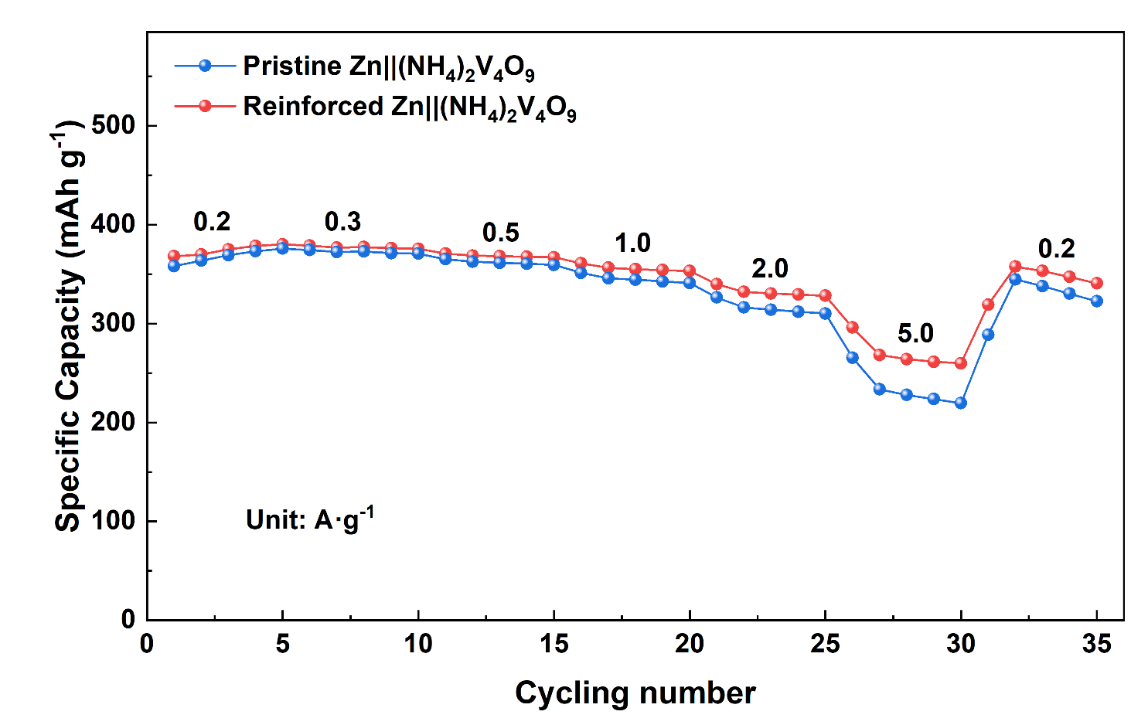


**Fig. S51** Rate performances of the (red) reinforced and (blue) pristine Zn||(NH_4_)_2_V_4_O_9_ full cells


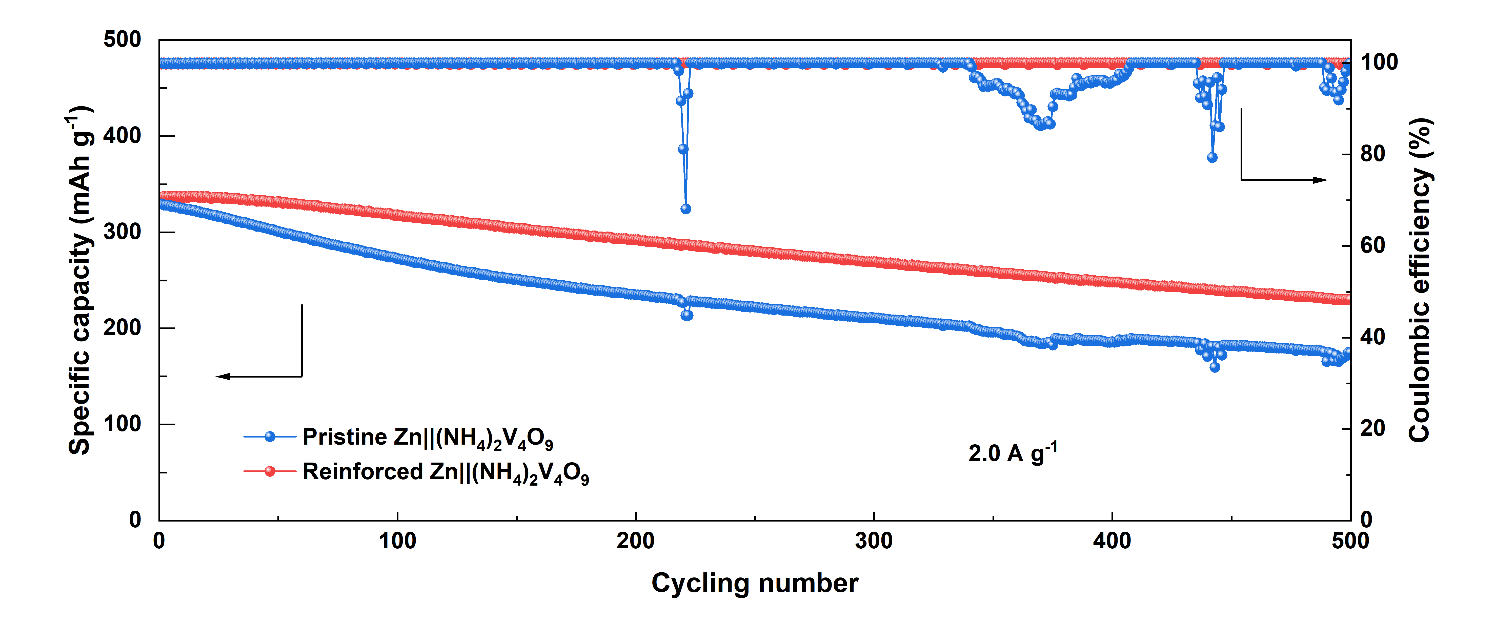


**Fig. S52** Long-term cycling performance of the (red) reinforced and (blue) pristine Zn||(NH_4_)_2_V_4_O_9_ full cells at 2.0 A g^-1^


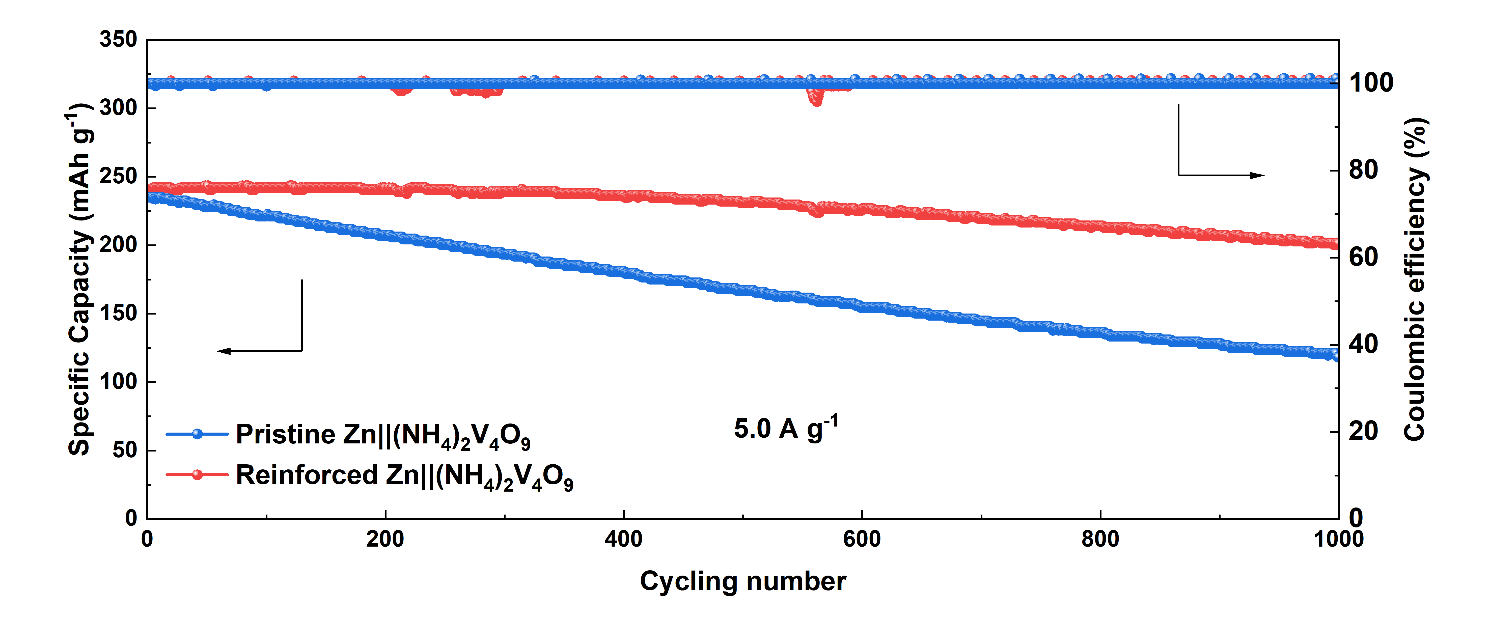


**Fig. S53** Long-term cycling performance of the (red) C_16_K self-assembly reinforced and (blue) pristine Zn||(NH_4_)_2_V_4_O_9_ full cells at 5.0 A g^-1^

**Table S5** Comparison of the cycling performance of full cells employing bio-inspired electrolytes

| Strategy | Cathode  materials | Cycling number | Retention (%) | Rate | Ref. |
| --- | --- | --- | --- | --- | --- |
| Synergistic reinforcement of ion transport and interfacial stability | β-MnO_2_ | 200 | 75% | 1.0 A g^-1^ | This work |
|  |  | 500 | 61% | 3.0 A g^-1^ |  |
|  | (NH_4_)_2_V_4_O_9_ | 500 | 70% | 2.0 A g-1 |  |
|  |  | 1000 | 84% | 5.0 A g^-1^ |  |
| BE/5Cor | NaV_3_O_8_ 1.5H_2_O | 500 | 70.6% | 1.0 A g^-1^ | [S1] |
|  |  | 500 | 70.8% | 5.0 A g^-1^ |  |
| Cll+ZOF | VO_2_ | 1200 | 94% | 2.0 A g^-1^ | [S2] |
| SAM-Zn | NH_4_V_4_O_10_ | 1000 | ~86% | 5.0 A g^-1^ | [S3] |
| NCAP-Glu-ZSO | NH_4_V_4_O_10_ | 200 | ~71.4% | 0.5 A g^-1^ | [S4] |
| SAB-Zn | MnO_2_ | 500 | 64.7% | 1 C | [S5] |
| ZnSO_4_/BICINE-10 | NH_4_V_4_O_10_ | 1000 | 69.8% | 3.0 A g^-1^ | [S6] |
| Val-H | AC@I_2_ | 1000 | 78% | 0.2 A g^-1^ | [S7] |
| ZSO/Phe | LMO | 300 | 77.3% | 1 C | [S8] |
| ZnSO_4_+Glucose | MnO_2_ | 1000 | ~80% | 10 C | [S9] |
| L-C/Zn(OTf)_2_ | NH_4_V_4_O_10_ | 500 | 80.59% | 1.0 A g^-1^ | [S10] |
| 1 M ZnSO_4_+75 mM Ert | δ-MnO_2_ | 500 | 79.5% | 1.0 A g^-1^ | [S11] |
| 2 M ZS+10% Xos | Zn_x_V_2_O_5_ | 300 | ~60% | 2.0 A g^-1^ | [S12] |
| PZS | Zn_0.25_V_2_O_5_ | 1000 | 77.8% | 1.2 A g^-1^ | [S13] |


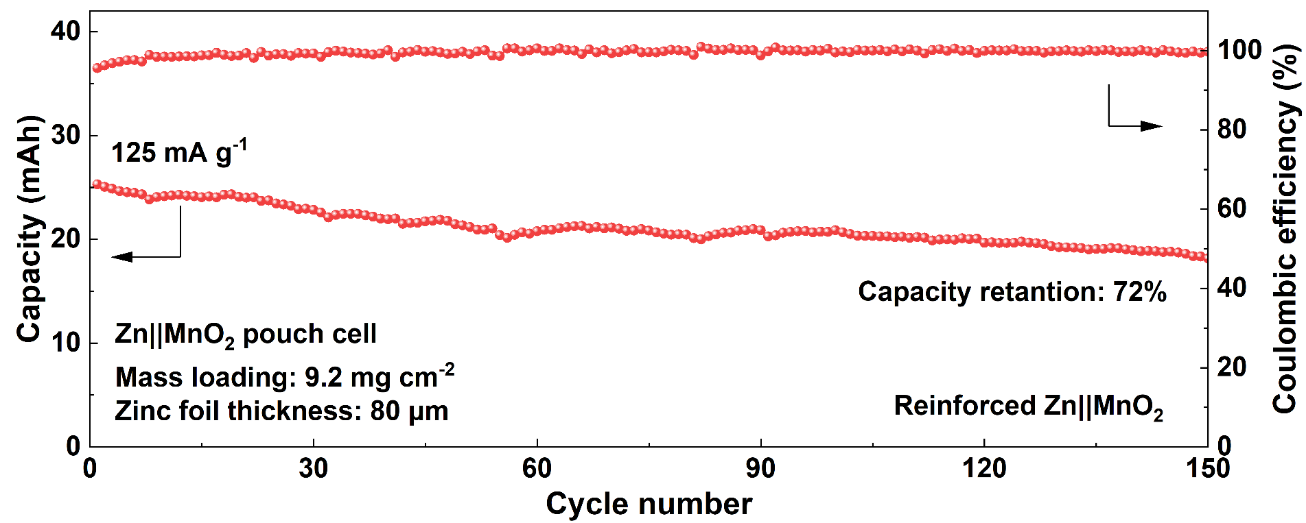


**Fig. S54** Cycling performance of the C_16_K self-assembly reinforced Zn||MnO_2_ pouch cell at 125 mA g^-1^


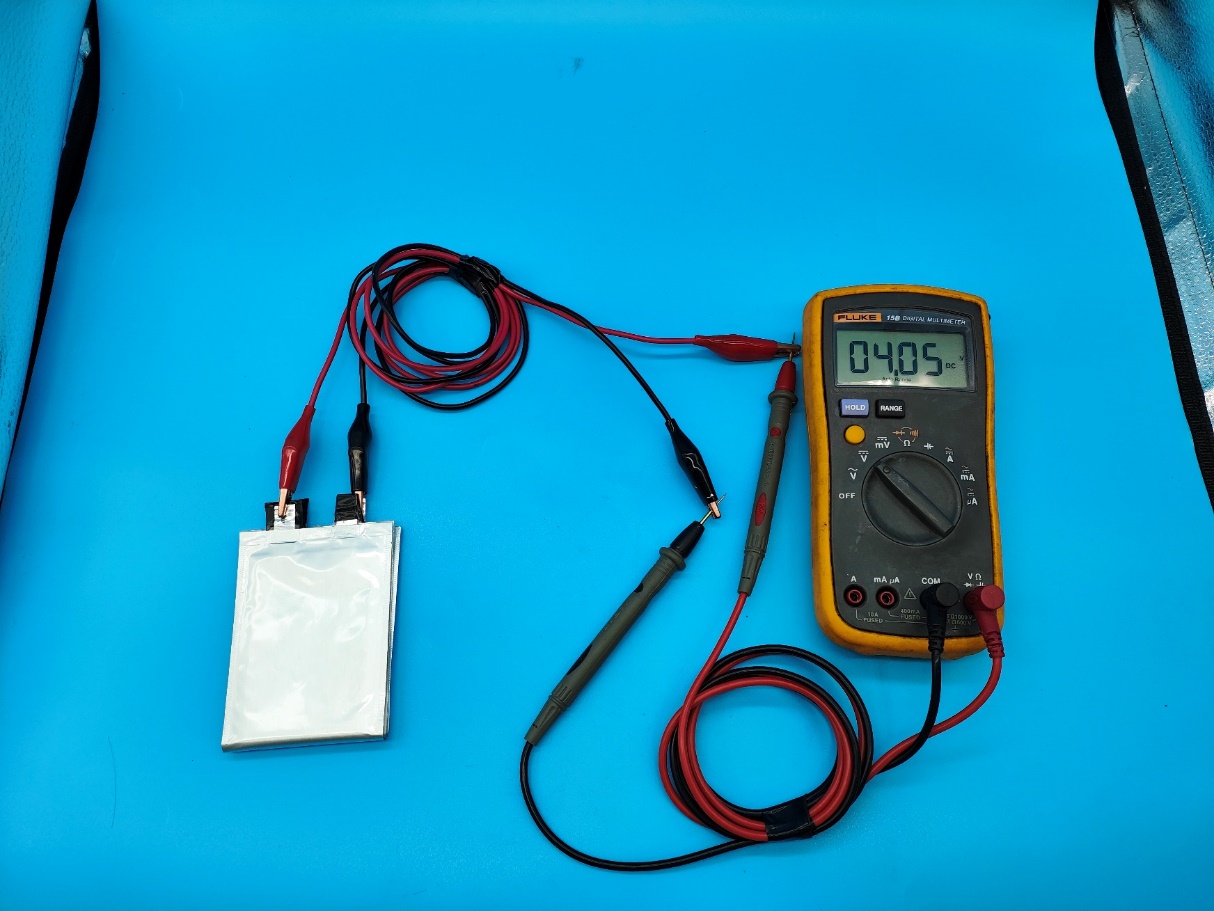


**Fig. S55** Photographic image showing the voltage measurement of three interconnected reinforced Zn||MnO_2_ pouch cells using a voltmeter. The interconnected pouch cells output a stable voltage of up to 4.05 V


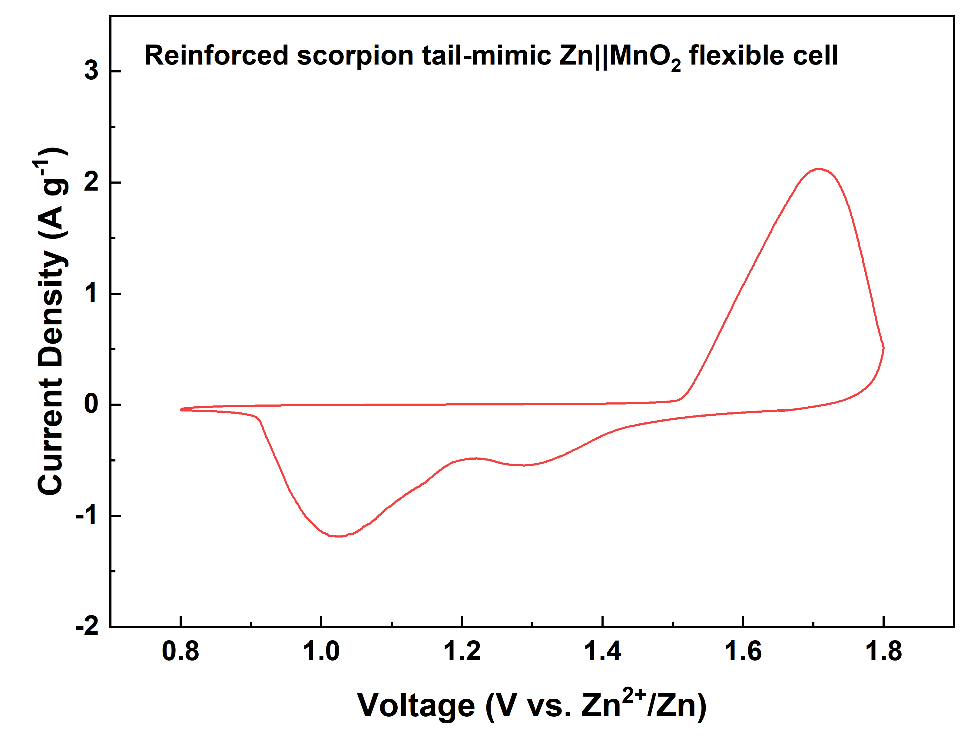


**Fig. S56** CV characterization of the scorpion tail-mimic reinforced Zn||MnO_2_ flexible battery at a scan rate of 0.5 mV s^-1^


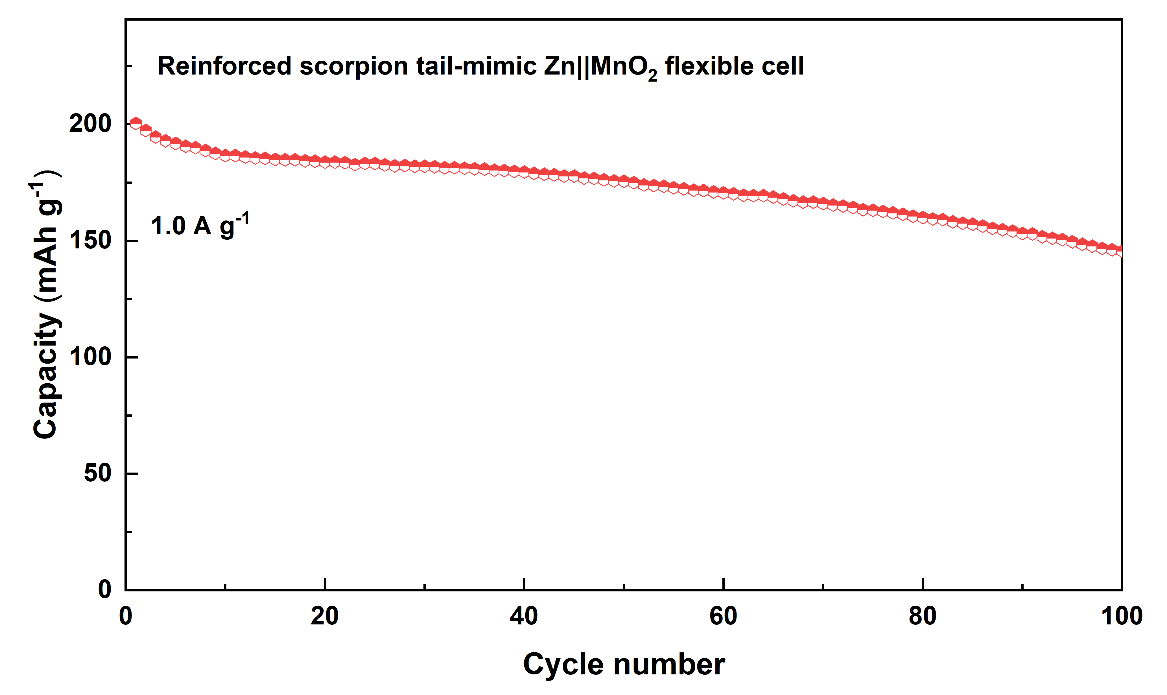


**Fig. S57** Long-term cycling performance of the scorpion tail-mimic reinforced Zn||MnO_2_ flexible battery at 1.0 A g^-1^


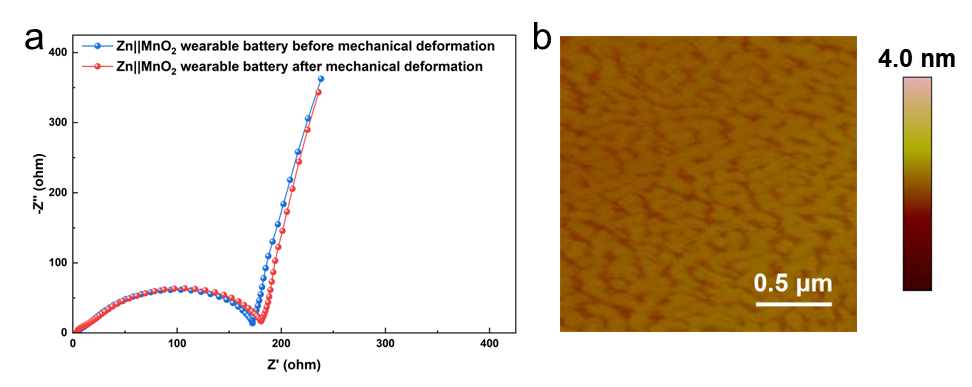


**Fig. S58 a** EIS of the Zn||MnO_2_ wearable battery before and after mechanical deformation. **b** AFM image of the interfacial self-assembly of C_16_K after mechanical deformation

**Supplementary References**

[S1] Z. Luo, Y. Xia, S. Chen, X. Wu, E. Akinlabi et al. A homogeneous plating/stripping mode with fine grains for highly reversible Zn anodes. Energy Environ. Sci. **17**(18), 6787-6798 (2024). <https://doi.org/10.1039/D4EE02264E>

[S2] J. Gao, B. Qiu, J. Huang, J. Wen, M. Yang et al. Collagen-mediated solvent sheathing and derived interfacial manipulation toward ultrahigh-rate Zn anodes. Adv. Funct. Mater. **35**(2), 2412791 (2024). <https://doi.org/10.1002/adfm.202412791>

[S3] D. Li, Y. Tang, S. Liang, B. Lu, G. Chen et al. Self-assembled multilayers direct a buffer interphase for long-life aqueous zinc-ion batteries. Energy Environ. Sci. **16**(8), 3381-3390 (2023). <https://doi.org/10.1039/D3EE01098H>

[S4] X. Fan, L. Chen, Y. Wang, X. Xu, X. Jiao et al. Selection of negative charged acidic polar additives to regulate electric double layer for stable zinc ion battery. Nano-Micro Lett. **16**(1), 270 (2024). <https://doi.org/10.1007/s40820-024-01475-5>

[S5] S. Chen, Y. Xia, R. Zeng, Z. Luo, X. Wu et al. Ordered planar plating/stripping enables deep cycling zinc metal batteries. Sci. Adv. **10**(10), eadn2265 (2024). <https://doi.org/10.1126/sciadv.adn2265>

[S6] Z. Peng, S. Guo, D. Xu, D. Zhao, H. Qi et al. Trace-level green bio-derived amino acid molecular leveling agent enables dendrite-free and long-lifespan zinc metal anodes. Adv. Mater. e11603 (2025). <https://doi.org/10.1002/adma.202511603>

[S7] J. Lin, C. Ji, G. Guo, Y. Luo, P. Huang et al. Interfacial H-bond network/concentration fields/electric fields regulation achieved by d-valine anions realizes the highly efficient aqueous zinc ion batteries. Angew. Chem. Int. Ed. **64**(24), e202501721 (2025). <https://doi.org/10.1002/anie.202501721>

[S8] A. Zhou, H. Wang, F. Zhang, X. Hu, Z. Song et al. Amphipathic phenylalanine-induced nucleophilic–hydrophobic interface toward highly reversible Zn anode. Nano-Micro Lett. **16**(1), 164 (2024). <https://doi.org/10.1007/s40820-024-01380-x>

[S9] P. Sun, L. Ma, W. Zhou, M. Qiu, Z. Wang et al. Simultaneous regulation on solvation shell and electrode interface for dendrite-free Zn ion batteries achieved by a low-cost glucose additive. Angew. Chem. Int. Ed. **60**(33), 18247-18255 (2021). <https://doi.org/10.1002/anie.202105756>

[S10] Z. Chen, Y. Xu, R. Jiang, H. Zhu, Q. Zhang et al. Chelation and interfacial engineering for long-term cycling and self-healing aqueous zinc batteries. Adv. Funct. Mater. e12356 (2025). <https://doi.org/10.1002/adfm.202512356>

[S11] S. Zhang, Q. Gou, W. Chen, H. Luo, R. Yuan et al. Co-regulating solvation structure and hydrogen bond network via bio-inspired additive for highly reversible zinc anode. Adv. Sci. **11**(35), 2404968 (2024). <https://doi.org/10.1002/advs.202404968>

[S12] W. Guo, L. Xu, Y. Su, L. Zhao, Y. Ding, et al. Synchronous modulation of h-bond interaction and steric hindrance via bio-molecular additive screening in Zn batteries. Angew. Chem. Int. Ed. **64**(5), e202417125 (2025). <https://doi.org/10.1002/anie.202417125>

[S13] S. Wang, S. Wang, Z. Wei, Y. Wang, D. Zhang et al. A parts-per-million scale electrolyte additive for durable aqueous zinc batteries. Nat. Comm. **16**(1), 1800 (2025). <https://doi.org/10.1038/s41467-025-56607-1>
